# Dual-energy lattice-tip ablation system for persistent atrial fibrillation: a randomized trial

**DOI:** 10.1038/s41591-024-03022-6

**Published:** 2024-05-17

**Authors:** Elad Anter, Moussa Mansour, Devi G. Nair, Dinesh Sharma, Tyler L. Taigen, Petr Neuzil, Erich L. Kiehl, Josef Kautzner, Jose Osorio, Stavros Mountantonakis, Andrea Natale, John D. Hummel, Anish K. Amin, Usman R. Siddiqui, Doron Harlev, Paul Hultz, Shufeng Liu, Birce Onal, Khaldoun G. Tarakji, Vivek Y. Reddy, SPHERE PER-AF Investigators, Vivek Y. Reddy

**Affiliations:** 1 Shamir Medical Center, Be’er Ya’Akov, Israel; 2 Massachusetts General Hospital, Boston, MA USA; 3 St. Bernards Medical Center & Arrhythmia Research Group, Jonesboro, AR USA; 4 NCH Rooney Heart Institute, Naples, FL USA; 5 Cleveland Clinic, Cleveland, OH USA; 6 Na Homolce Hospital, Prague, Czechia; 7 Sentara, Norfolk, VA USA; 8 IKEM Prague, Prague, Czechia; 9 HCA Florida Miami, Miami, FL USA; 10 Northwell, New Hyde Park, NY USA; 11 Texas Cardiac Arrhythmia Institute, Austin, TX USA; 12 Department of Biomedicine and Prevention, Division of Cardiology, University of Tor Vergata, Rome, Italy; 13 Division of Cardiology, Ohio State University, Columbus, OH USA; 14 Riverside Methodist Hospital, Upper Arlington, OH USA; 15 Florida Cardiology, Orlando, FL USA; 16 Medtronic, Mounds View, MN USA; 17 Helmsley Electrophysiology Center, Mount Sinai Fuster Heart Hospital, New York, NY USA

**Keywords:** Atrial fibrillation, Cardiac device therapy, Randomized controlled trials

## Abstract

Clinical outcomes of catheter ablation for atrial fibrillation (AF) are suboptimal due, in part, to challenges in achieving durable lesions. Although focal point-by-point ablation allows for the creation of any required lesion set, this strategy necessitates the generation of contiguous lesions without gaps. A large-tip catheter, capable of creating wide-footprint ablation lesions, may increase ablation effectiveness and efficiency. In a randomized, single-blind, non-inferiority trial, 420 patients with persistent AF underwent ablation using a large-tip catheter with dual pulsed field and radiofrequency energies versus ablation using a conventional radiofrequency ablation system. The primary composite effectiveness endpoint was evaluated through 1 year and included freedom from acute procedural failure and repeat ablation at any time, plus arrhythmia recurrence, drug initiation or escalation or cardioversion after a 3-month blanking period. The primary safety endpoint was freedom from a composite of serious procedure-related or device-related adverse events. The primary effectiveness endpoint was observed for 73.8% and 65.8% of patients in the investigational and control arms, respectively (*P* < 0.0001 for non-inferiority). Major procedural or device-related complications occurred in three patients in the investigational arm and in two patients in the control arm (*P* < 0.0001 for non-inferiority). In a secondary analysis, procedural times were shorter in the investigational arm as compared to the control arm (*P* < 0.0001). These results demonstrate non-inferior safety and effectiveness of the dual-energy catheter for the treatment of persistent AF. Future large-scale studies are needed to gather real-world evidence on the impact of the focal dual-energy lattice catheter on the broader population of patients with AF. ClinicalTrials.gov identifier: NCT05120193.

## Abstract (web summary)

In a randomized trial testing two types of catheter ablation treatments for persistent atrial fibrillation, a large-tip, dual pulsed field and radiofrequency catheter with a large footprint was non-inferior to a conventional radiofrequency catheter.

## Main

Atrial fibrillation (AF) is the most common type of cardiac arrhythmia and is the leading cardiac cause of stroke^1^. Catheter-based ablation is an effective and safe treatment for patients with AF^1,2^. The cornerstone of this procedure is the electrical isolation of the pulmonary veins (PVs)^3^. In patients with persistent AF, catheter ablation often results in less favorable clinical outcomes compared to those in patients with paroxysmal AF^4^. This disparity has been commonly ascribed to a broader and more complex arrhythmogenic substrate in persistent AF^5–7^.

Conventional catheter ablation technologies are optimized for treating paroxysmal AF but have major shortcomings in treating persistent AF^8^. Specifically, focal radiofrequency catheter-based ablation procedures typically involve the use of a specialized mapping catheter to create a three-dimensional electro-anatomical map of the left atrium. Subsequently, a separate focal ablation catheter with a solid metal tip applies radiofrequency energy in a sequential point-by-point fashion to form a contiguous set of ablation lesions. Major shortcomings of these AF ablation procedures include (1) limited effectiveness due to the technical challenges of placing contiguous lesions, leading to conduction gaps and subsequent arrhythmia recurrence^9,10^; (2) risk of atrio-esophageal fistula, phrenic nerve paralysis and PV narrowing^11^; and (3) the need to use two separate catheters for mapping and ablation, which increases procedural complexity and cost^2^. ‘Single-shot’ ablation catheters are designed to create a circular lesion pattern for pulmonary vein isolation (PVI) using one or a few applications, but they fall short in generating the additional linear ablations often required for persistent AF.

The novel technological platform investigated in this study integrates high-density electro-anatomical mapping of the heart with dual-energy ablation, employing either radiofrequency or pulsed field energies, within a single lattice-tip catheter system. Pulsed field ablation (PFA), a non-thermal energy source, ablates tissue by using microsecond pulses of electric fields to destabilize cell membranes^12,13^. Notably, PFA has a preferential effect on myocardial tissue while minimizing impact on adjacent non-cardiac tissues, such as the esophagus and the phrenic nerve^14–16^. Preclinical and first-in-human studies have demonstrated that this lattice-tip catheter with its large footprint generates full-thickness atrial myocardial lesions and achieves PVI faster, with fewer applications and with greater durability compared to conventional catheters, and does so without causing thermal injury to surrounding structures^17–23^. Accordingly, SPHERE Per-AF was a randomized, single-blind, non-inferiority clinical trial that compared the lattice-tip dual-energy ablation platform with a conventional radiofrequency ablation platform in the treatment of drug-refractory persistent AF.

## Results

### Participants

**Fig. 1:**
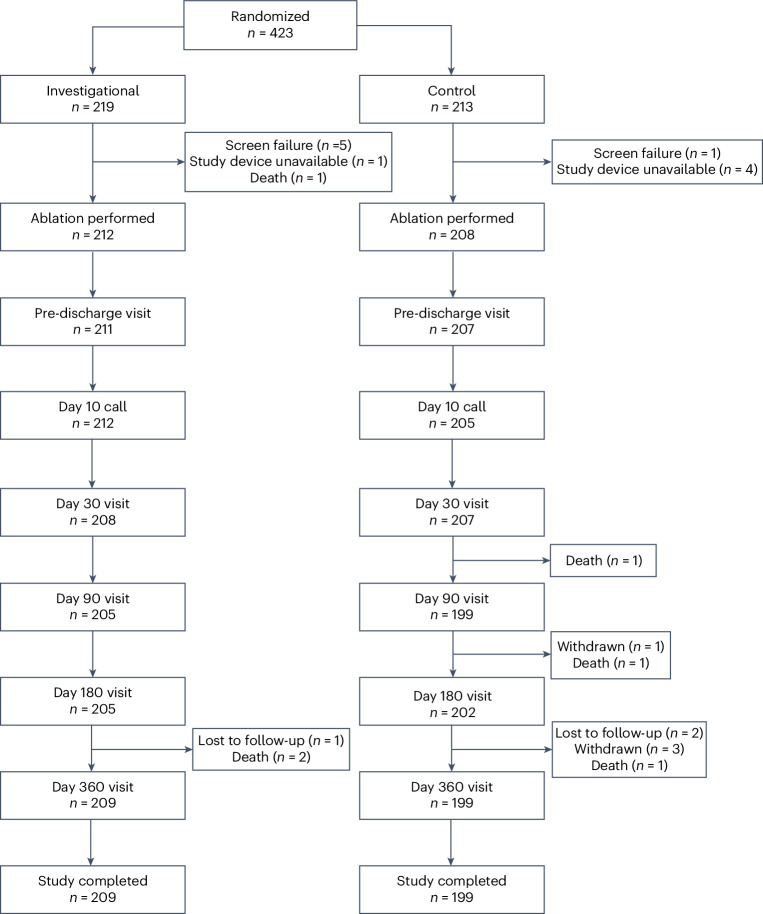
Participant flow diagram. Of the 432 patients randomized to either treatment, 219 were assigned to undergo treatment with the investigational system and 213 were assigned to undergo treatment with the control system. Before the ablation procedure, seven patients in the investigational arm and five patients in the control arm were withdrawn. The primary analysis consisted of 212 patients in the investigational arm and 208 patients in the control arm. Of these 420 patients, 408 (97.1%) completed the trial with 12 months of follow-up.

From December 2021 to December 2022, patients were screened for the trial, and 469 were enrolled. After a roll-in phase that included 37 patients (up to two patients per site to familiarize the operators with the technology), 432 patients were randomly assigned to undergo ablation using either the investigational (219 patients) or the control (213 patients) systems, as shown in the participant flow chart (Fig. 1). After accounting for 12 dropouts (seven investigational and five control), a total of 420 patients (212 investigational and 208 control) received the intended treatment across 20 centers by 40 operators, with each operator treating an average of 6 ± 7 patients using the investigational system. The gap between randomization and procedure was 4.9 ± 5.7 d for the investigational arm and 6.0 ± 10.7 d for the control arm. Premature exit from the study occurred in 12 participants (three investigational and nine control). Details on participant dropouts and premature study exits are provided in Fig. 1 and Extended Data Table 2.

Baseline characteristics are provided in Table 1 and were largely balanced between the two groups, including age, comorbidities, time since AF diagnosis, left atrial dimension, history of cardioversions and usage of class I/III anti-arrhythmic drugs.

**Table 1:** Baseline patient characteristics

Characteristic ^a^	Investigational (*n* = 212)	Control (*n* = 208)
Age (years)	67.8 ± 8.3	66.7 ± 8.8
Sex, male	139 (65.6%)	147 (70.7%)
Race, White or Caucasian	199 (93.9%)	199 (95.7%)
Body mass index (kg m^−^^2^) ^b^	30.0 ± 4.8	30.3 ± 4.9
Left atrial diameter (mm) ^c^	43.0 ± 6.1	44.0 ± 5.4
Left ventricular ejection fraction (%)	57.7 ± 7.2	55.5 ± 8.0
Number of failed class I or class III anti-arrhythmic drugs ^d^	1.2 ± 0.4	1.1 ± 0.4
Prior cardioversion for atrial arrhythmias		
Electrical	146 (68.9%)	140 (67.3%)
Pharmacologic	13 (6.1%)	15 (7.2%)
Time from first diagnosis of persistent AF (years)	1.3 ± 2.6	1.3 ± 2.2
CHA_2_DS_2-_VASc score ^e^	2.4 ± 1.4	2.3 ± 1.4
Medical characteristics		
Congestive heart failure	36 (17.0%)	26 (12.5%)
Coronary artery disease	37 (17.5%)	35 (16.8%)
Diabetes	38 (17.9%)	34 (16.3%)
Hypertension	160 (75.5%)	157 (75.5%)
Myocardial infarction	9 (4.2%)	7 (3.4%)
Obstructive sleep apnea	47 (22.2%)	57 (27.4%)
Renal disease	22 (10.4%)	15 (7.2%)
Stroke/transient ischemic attack	16 (7.5%)	11 (5.3%)
Baseline medications		
Class I anti-arrhythmic drugs	41 (19.3%)	39 (18.8%)
Class II anti-arrhythmic drugs	102 (48.1%)	98 (47.1%)
Class III anti-arrhythmic drugs	103 (48.6%)	96 (46.2%)
Class IV anti-arrhythmic drugs	26 (12.3%)	21 (10.1%)
Direct oral anti-coagulation	209 (98.6%)	203 (97.6%)

^a^Numbers presented are mean ± s.d. or *n* (%).

^b^Data available for 211 patients (investigational) and 207 patients (control).

^c^Data available for 210 patients (investigational).

^d^Data available for 202 patients (investigational) and 192 patients (control).

^e^Data available for 207 patients (control). CHA_2_DS_2_-VASc scores range from 0 to 9, with higher scores indicating a greater risk of stroke.

For both arms, overall adherence to trial follow-up visits was 97% (2,303/2,385). Compliance to Holter and electrocardiogram (ECG) monitoring was 84% (696/828) and 85% (1,062/1,247), respectively. Compliance to trans-telephonic transmissions was 92% (3,417/3,706) and resulted in a rate of close to two transmissions per patient per month for both arms (Extended Data Table 3). Adherence was similar between groups.

### Primary effectiveness and safety endpoints

**Fig. 2:**
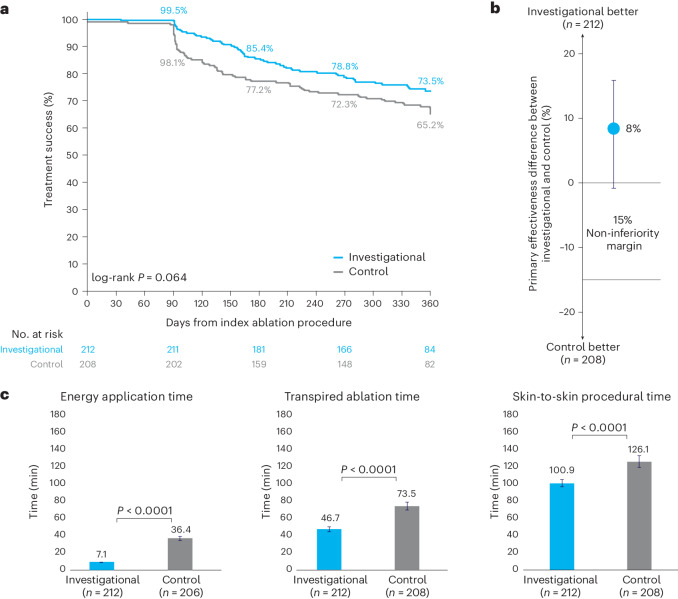
Primary and secondary effectiveness outcomes. **a**, Kaplan–Meier analysis of the primary effectiveness endpoint. Shown are the Kaplan–Meier estimates of freedom from the primary effectiveness endpoint, which is a composite of the freedom from initial procedural failure, repeat ablation at any time and arrhythmia recurrence, anti-arrhythmic drug initiation or escalation or cardioversion after a 3-month blanking period. Comparison of the investigational arm versus control was performed using the two-sided log-rank test. **b**, Farrington–Manning analysis of the primary effectiveness endpoint. Trial success with respect to effectiveness was defined as non-inferiority of the primary effectiveness endpoint based on binomial proportions using the one-sided Farrington–Manning test with a non-inferiority margin of 15% and a one-sided alpha of 0.025. The observed difference in primary effectiveness success was 8.0% in favor of the investigational arm (95% two-sided CI: −0.9% to 16.8%), based on primary effectiveness for 210 investigational and 202 control patients. Visualized here is the pre-specified 15% non-inferiority margin, the point estimate of the difference between treatment and control and the two-sided 95% CI of the difference. **c**, Procedural characteristics. Left, energy application time includes both radiofrequency and PFA for the investigational device and radiofrequency time for the control device. Visualized here is the mean and 95% CI for the investigational arm (7.1 (6.8, 7.4), *n* = 212) and the control arm (36.4 (33.9, 38.8), *n* = 206) (*P* < 0.0001). Middle, transpired ablation time is the time between the first and last application, which includes the elapsed time for both PVI and any additional linear ablation. Visualized here is the mean and 95% CI for the investigational arm (46.7 (44.0, 49.4), *n* = 212) and the control arm (73.5 (68.8, 78.2), *n* = 208) (*P* < 0.0001). Right, skin-to-skin procedure time is the time elapsed from first venous access to last sheath removal. Visualized here is the mean and 95% CI for the investigational arm (100.9 (96.8, 105.1), *n* = 212) and the control arm (126.1 (119.4, 132.8), *n* = 208) (*P* < 0.0001). Contingent upon trial success, sequential testing with an overall one-sided alpha of 0.025 was performed on a pre-specified set of endpoints to further examine superiority of the investigational arm versus control. Adjustments were made for multiple comparisons with the sequential testing method.

The primary effectiveness analysis included 210 investigational patients and 202 control patients in the primary analysis cohort with primary effectiveness outcome data available. The primary effectiveness endpoint success rate was 73.8% for the investigational arm and 65.8% for the control arm. The observed difference in primary effectiveness success was 8.0% in favor of the investigational arm (95% confidence interval (CI): −0.9% to 16.8%), meeting the criteria for non-inferiority (*P* < 0.0001; Table 2 and Fig. 2b). The 1-year Kaplan–Meier estimates were 73.5% for the investigational arm and 65.2% for the control arm (Fig. 2a). A post hoc analysis showed that freedom from atrial arrhythmias for the investigational arm (76.7%, 161/210) was non-inferior to the control arm (72.8%, 147/202) (*P* < 0.0001; Extended Data Table 4). All patients in both study groups underwent PVI. In the investigational arm, acute PVI was successfully achieved using only the assigned study device. In the control arm, one PV could not be isolated using the assigned device and was ultimately treated with adjunctive cryo-balloon ablation. In the investigational arm, acute block was achieved across all ablation lines using only the assigned study device. In the control arm, one mitral line could not be completed using the assigned device and required alcohol ablation of the vein of Marshall.

**Table 2:** Primary effectiveness endpoint summary

Component	Investigational (*n* = 210)^a^	Control (*n* = 202)^a^
Composite primary effectiveness success, *n* (%)	155 (73.8%)	133 (65.8%)
95% CI of success rate	(67.5%, 79.3%)	(59.1%, 72.0%)
Difference (95% CI) Farrington–Manning non-inferiority test *P* value^b^	8.0% (−0.9% to 16.8%) *P* < 0.0001	
Primary effectiveness first failure mode^c^, *n* (%)		
Inability to isolate all targeted pulmonary veins during the index procedure	0 (0.0%)	0 (0.0%)
Any left atrial ablation done with non-assigned study device during the index procedure	0 (0.0%)	2 (1.0%)
Any repeat ablation or surgery for AF/AT/AFL recurrence after the index procedure	2 (1.0%)	5 (2.5%)
Direct current cardioversion for AF/AT/AFL recurrence during the effectiveness evaluation period	2 (1.0%)	1 (0.5%)
Documented AF/AT/AFL recurrence during the effectiveness evaluation period	47 (22.4%)	51 (25.2%)
Initiation of new class I/III anti-arrhythmic drug during the effectiveness evaluation period or class I/III anti-arrhythmic drug initiation increase from the historic maximum ineffective dose	4 (1.9%)	10 (5.0%)

AFL, atrial flutter; AT, atrial tachycardia.

^a^ Two patients in the investigational arm and six patients in the control arm were excluded from the primary effectiveness analysis due to incomplete follow-up without experiencing any failure event.

^b^ For patients with multiple reasons for failure, only the first occurrence is reported.

^c^ Based on a pre-defined non-inferiority margin of 15%.

The primary safety analysis included 212 investigational patients and 208 control patients in the primary analysis cohort. Primary safety events occurred in three (1.4%) patients in the investigational arm and in two (1.0%) patients in the control arm (difference: 0.4%; 90% CI: −2.8% to 3.7%; *P* < 0.0001 for non-inferiority; Table 3). The events in the investigational arm included hospitalizations for pulmonary edema due to hypertensive urgency, exacerbation of chronic obstructive pulmonary disease and hemoptysis (with no signs of active bleeding by endoscopy and no recurrence). In the control arm, two hospitalizations occurred due to pulmonary edema. In both groups, there were no reports of atrial-esophageal fistula, PV stenosis, tamponade or permanent phrenic nerve paralysis. Based on the pre-defined non-inferiority safety endpoint, treatment with the investigational device was non-inferior to the control device.

**Table 3:** Primary safety endpoint summary

Primary safety event	Investigational (*n* = 212)	Control (*n* = 208)
Patients with any primary safety event, *n* (%)	3 (1.4%)	2 (1.0%)
Within 7 d		
Death	0	0
Myocardial infarction	0	0
Phrenic nerve paralysis	0	0
Transient ischemic attack	0	0
Stroke/cerebrovascular accident	0	0
Thromboembolism	0	0
Major vascular access complications/bleeding	0	0
Heart block	0	0
Gastroparesis	0	0
Severe pericarditis	0	0
Hospitalization (initial and prolonged) due to cardiovascular or pulmonary adverse event^a^	3	2
Within 30 d		
Cardiac tamponade/perforation	0	0
Within 90 d		
Atrio-esophageal fistula	0	0
Within 180 d		
PV stenosis	0	0
Myocardial infarction	0	0
Summarized results		
90% CI of event rate	(0.4%, 3.6%)	(0.2%, 3.0%)
Difference (90% CI) Farrington–Manning one-sided non-inferiority test *P* value^b^	0.4% (−2.8%, 3.7%) *P* < 0.0001	

^a^ Excludes hospitalization due to atrial arrhythmia recurrence.

^b^ Based on a pre-defined non-inferiority margin of 8%.

A full list of adverse events that were related or possibly related to the study procedure or device is provided in Extended Data Table 5. After ablation, five deaths occurred in the trial that were not related to the procedure or device. In the investigational arm, one patient died due to monoclonal gammopathy and systemic amyloidosis, and another patient died due to choking while eating followed by cardiopulmonary arrest. In the control arm, one patient died due to ampullary adenocarcinoma, the second due to heart failure and coronary artery disease and the third due to heart failure.

### Secondary superiority analyses

Pre-specified superiority testing showed shorter procedural durations for the investigational device compared to the control device. This included shorter total energy application time (7.1 ± 2.0 min versus 36.4 ± 17.7 min; difference: −29.2 min, 95% CI −31.7 to −26.8, *P* < 0.0001) (Table 4 and Fig. 2c); shorter transpired ablation time, defined as the time elapsing between the first and last ablation application (46.7 ± 20.0 min versus 73.5 ± 34.4 min; difference: −26.8 min, 95% CI: −32.2 to −21.4, *P* < 0.0001) (Table 4 and Fig. 2c); and shorter skin-to-skin procedural time (100.9 ± 30.8 min versus 126.1 ± 49.2 min; difference: −25.1 min, 95% CI −33.0 to −17.3, *P* < 0.0001) (Table 4 and Fig. 2c). Pre-specified superiority testing did not demonstrate superiority of the primary effectiveness endpoint in the investigational arm compared to the control arm (two-sided *P* = 0.078, which was greater than the two-sided alpha of 0.05; Fig. 2b).

**Table 4:** Procedural characteristics

Parameter^a^	Investigational (*n* = 212)	Control (*n* = 208)	One-sided *P* value for investigational device superiority^b^
Skin-to-skin procedural time (min) (95% CI)	100.9 ± 30.8 (96.8, 105.1)	126.1 ± 49.2 (119.4, 132.8)	*P* < 0.0001
Transpired ablation time (min) (95% CI)	46.7 ± 20.0 (44.0, 49.4)	73.5 ± 34.4 (68.8, 78.2)	*P* < 0.0001
Total ablation energy application time (min) (95% CI)^c^	7.1 ± 2.0 (6.8, 7.4)	36.4 ± 17.7 (33.9, 38.8)	*P* < 0.0001
Fluoroscopy time (min)	4.9 ± 6.6	6.3 ± 9.1	
Time from beginning to end of PVI (min)^d^	25.9 ± 10.7	53.6 ± 28.8	
Usage of adenosine	97 (45.8%)	105 (50.5%)	
Usage of isoproterenol	31 (14.6%)	37 (17.8%)	
Total fluid delivered by ablation catheters (ml)^e^	482.0 ± 142.6	727.1 ± 378.7	
Esophageal temperature probe usage	63 (29.7%)	158 (76.0%)	
Esophageal deviation device used	3 (1.4%)	34 (16.3%)	
Number of transseptal accesses			
1	202 (95.3%)	129 (62.0%)	
>1	10 (4.7%)	79 (38.0%)	
Number of mapping and/or ablation catheters used in left atrium^f^			
One catheter	206 (97.2%)	0 (0%)	
Two catheters	6 (2.8%)	200 (96.2%)	
Three catheters	0 (0%)	4 (1.9%)	
Ablation lesion sets beyond PVI^g^	203 (95.8%)	178 (85.6%)	
Cavo-tricuspid isthmus line	115 (54.2%)	98 (47.1%)	
Mitral line	72 (34.0%)	22 (10.6%)	
Left atrial roof, posterior or inferior line	198 (93.4%)	137 (65.9%)	

^a^ Numbers presented are mean ± s.d. or *n* (%)

^b^ Adjustments were made for multiple comparisons based on sequential *t*-tests with an overall one-sided alpha of 0.025.

^c^ Represents *n* = 206 patients for the control arm.

^d^ Represents *n* = 207 patients for the control arm.

^e^ Represents *n* = 208 patients for the investigational arm and *n* = 204 patients for the control arm.

^f^ Represents *n* = 204 patients for the control arm.

^g^ For treatment of documented macro-reentrant tachycardias, including cavo-tricuspid isthmus lines, and, per investigator discretion: left atrial roof, inferior or posterior lines and mitral isthmus lines.

### Treatment characteristics

Procedural characteristics are presented in Table 4. Lower fluoroscopy usage was observed in the investigational arm (4.9 ± 6.6 min) compared to the control arm (6.3 ± 9.1 min). Lower fluid delivery from the ablation catheter was observed with the investigational device compared to the control device (482.0 ± 142.6 ml versus 727.1 ± 378.7 ml, respectively). Furthermore, the use of an esophageal temperature probe was less frequently observed in the investigational arm than in the control arm (29.7%, 63/212 versus 76.0%, 158/208, respectively). Similarly, use of an esophageal deviation device was less frequently observed in the investigational arm than in the control arm (1.4%, 3/212 versus 16.3%, 34/208, respectively). In the investigational group, a single transseptal access approach was used in 95.3% (202/212) of cases compared to 62% (129/208) in the control group. Improved quality of life was observed after ablation in both the investigational and control groups, as indicated by the mental component of the SF-12v2 Health Survey (3.2 ± 8.1 and 4.3 ± 8.8 increase from baseline to 12 months in the investigational and control arms, respectively); the physical component of the SF-12v2 Health Survey (4.7 ± 7.6 and 4.7 ± 8.5 increase from baseline to 12 months in the investigational and control arms, respectively); and the Atrial Fibrillation Effect on Quality-of-Life (AFEQT) survey (22.3 ± 19.5 and 22.2 ± 19.3 increase from baseline to 12 months in the investigational and control arms, respectively) (Extended Data Table 6). At the 1-year follow-up or study exit, 37 out of 212 patients (17.5%) in the investigational arm and 33 out of 208 patients (15.9%) in the control arm were taking class I or class III anti-arrhythmic drugs.

### Additional ablation lesion sets

Most patients in both arms received additional linear ablation beyond PVI (95.8% and 85.6% for the investigational and control groups, respectively). Left atrial posterior wall isolation including roof lines was performed in 93.4% and 65.9% in the investigational and control groups, respectively. A cavo-tricuspid isthmus line was created in 54.2% and 47.1% in the investigational and control groups, respectively. A mitral line was created in 34.0% in the investigational group and in 10.6% in the control group, respectively (Table 4). In post hoc analyses performed to assess the heterogeneity with respect to additional ablation lines on clinical outcomes, a regression approach was employed to compare subgroups with and without mitral or posterior/roof lines. The analysis did not reveal any heterogeneity of treatment effects based on the presence or absence of these linear ablations, as indicated by *P* > 0.1 for both, as shown in Extended Data Table 7.

### PVI durability

During the study period, a total of 26 patients underwent a redo catheter ablation procedure, with 10 in the investigational arm and 16 in the control arm (one additional control patient had redo surgical ablation). At this repeat procedure, PVI durability was 50% per patient and 66.7% per vein in the investigational arm compared to 18.8% per patient and 48.4% per vein in the control arm.

### Neurological substudy analysis

In brain magnetic resonance imaging (MRI) that examined the presence of silent ischemic lesions after ablation, three out of 37 patients in the investigational group and two out of 35 patients in the control group were found to have fluid-attenuated inversion recovery (FLAIR)-hyperintense acute lesions (Extended Data Table 8). Follow-up MRI scans performed 90 d later for these patients with silent ischemic lesions showed that two out of the three patients in the investigational group and one out of the two patients in the control group demonstrated full resolution.

## Discussion

**Extended Data Fig. 1:**
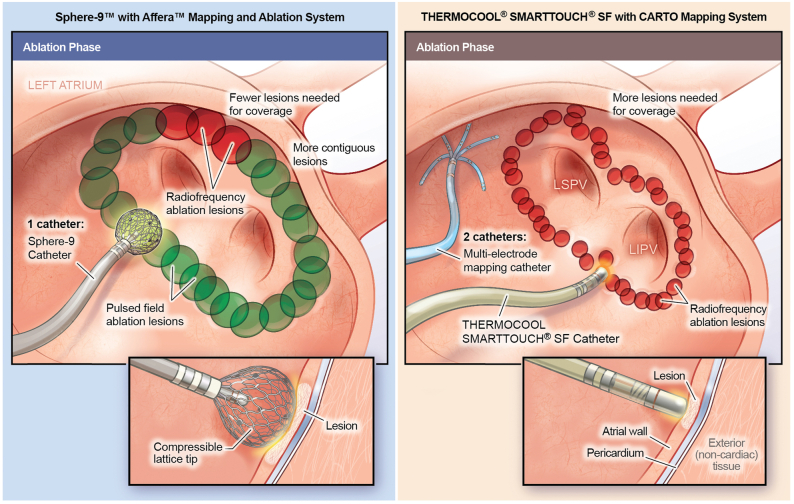
Comparison of catheter ablation systems in the SPHERE Per-AF clinical trial. The investigational Sphere-9TM lattice-tip dual energy catheter with AfferaTM Mapping and Ablation System is shown in the left panel. This system utilizes the Sphere-9TM catheter for both electroanatomical mapping and ablation, employing radiofrequency (red circles) or pulsed field (green circles) energies to create a line of electrical isolation around the pulmonary veins. The control system, depicted in the right panel, consists of a multielectrode mapping catheter for electroanatomical mapping and a separate catheter for radiofrequency ablation (THERMOCOOL® SMARTTOUCH® Surround Flow). As shown in the inserts, the investigational Sphere-9TM catheter has a wider footprint capable for creating wider lesions, resulting in a more contiguous ablation line. LIPV=left inferior pulmonary vein. LSPV=left superior pulmonary vein.

The SPHERE Per-AF trial was a randomized, single-blind, non-inferiority trial of patients with persistent AF comparing an all-in-one mapping and dual-energy (radiofrequency and pulsed field) large-footprint ablation catheter to conventional radiofrequency ablation (Extended Data Fig. 1). The investigational system was non-inferior to the conventional system in both safety and effectiveness. The investigational system was superior to the conventional system in measures of procedural efficiency, with shorter procedural duration, time from the first-to-last application and total energy application time.

Historical outcomes of catheter ablation in patients with persistent AF have been suboptimal, with 1-year success rates ranging between 45% and 62% in different multi-center trials, including the PRECEPT trial, which evaluated the same radiofrequency ablation system used in this trial as the control device^4,5,24–26^.

The present trial confirmed non-inferiority of effectiveness when compared to the standard-of-care mapping and ablation system and represented single-procedure success rate. The observed difference in effectiveness between the two groups was 8.0% (CI: −0.9% to 16.8%). The Kaplan–Meier curves show a visual separation between the two arms that emerged immediately after the 90-d blanking period and remained consistent throughout the follow-up period. However, a pre-defined secondary analysis of effectiveness did not demonstrate superiority, as indicated by a two-sided *P* value of 0.078, which is higher than the two-sided alpha threshold of 0.05.

The observed difference between the investigational and control arms was not driven by lower performance of the control arm. That is, in PRECEPT, which enrolled a similar cohort of patients with persistent AF who underwent catheter ablation using the same radiofrequency control catheter, the primary effectiveness success rate was 59.3% at 15 months, after a 9-month effectiveness evaluation period^27^. Although comparisons cannot be made across different trials given differences in clinical study design and patient baseline characteristics, the effectiveness observed in the control arm is similar to historical studies.

Both treatment arms demonstrated a low rate of primary safety events (1.4% for the investigational arm and 1.0% for the control arm) with no evidence of major complications, such as stroke, tamponade, atrio-esophageal fistula or permanent phrenic nerve paralysis. In comparison, the PRECEPT study reported a 4.7% primary safety event rate, including cardiac tamponade, stroke, phrenic nerve injury, pulmonary edema, pericarditis and major vascular access complications^4^. The lower frequency of major complications in both arms may reflect the extensive experience of the operators with focal ablation and may also attest to the rapid learning curve associated with the investigational device. The rate of FLAIR-hyperintense acute lesions was also relatively low (8%) compared to other technologies, ranging from 0% to 19% (refs. ^28–34^).

The design of the investigational system may have potential benefits that may contribute to safety and effectiveness^17,18,20,35,36^. In terms of safety, the integration of high-density mapping and dual-energy ablation into a single catheter, unlike the current standard that requires at least two separate catheters, reduces the number of transseptal punctures and/or catheter exchanges. This simplifies the procedural workflow and reduces the time involved. Indeed, 95.3% of procedures performed with the investigational system used a single transseptal puncture. In comparison, 62% of control procedures used a single transseptal puncture, with the remaining 38% using a double transseptal access. The wide and compressible lattice tip results in lower tissue pressure compared to a small, solid metal tip, thereby potentially reducing the risk of perforation. The ability to toggle between pulsed field and radiofrequency ablation allows the flexibility of using pulsed field on the posterior wall, an energy source shown to avoid the risk of thermal injury to adjacent organs, such as the esophagus^26^.

In terms of effectiveness, the wide footprint facilitates the creation of contiguous ablation lesions, decreasing the likelihood of gaps in the ablation line. In a previous clinical study, which included a second re-mapping procedure approximately 3 months after the index AF ablation procedure with this investigational device, the durability of PVI was 97% on a per-vein basis, with all four veins remaining isolated in 90% of patients^23^.

Procedural times with the investigational device were favorable also when compared to single-shot PFA technologies, typically ranging between 106 min and 145 min^26,37^. Additionally, the investigational device offers the flexibility to map and treat focal and reentrant atrial tachycardias, which are commonly encountered in this patient population^38^. Furthermore, this efficiency was noted despite the limited experience with the investigational system; before the study, only five out of 40 operators had clinical experience in the first-in-human study^23,35^. On average, these 40 operators treated 6 ± 7 patients each with the investigational system, whereas all operators had extensive experience with the conventional system.

Our trial has several limitations. There is a potential for under-detection of asymptomatic atrial tachyarrhythmias due to the absence of continuous invasive monitoring. Nevertheless, the randomized nature of the study suggests that any missed asymptomatic events would likely have impacted both groups equally. Furthermore, adherence to follow-up visits, as well as to Holter and trans-telephonic monitoring, was consistently high across both study groups. Although the ablation protocol, uniformly applied to both groups, mandated PVI and permitted the treatment of documented macro-reentrant tachycardias, it generally discouraged empiric ablation. Nevertheless, there was a relatively high rate of additional ablation lines in both groups, particularly in the interventional arm. Although this study was not designed or powered to assess the value of additional empiric ablation lines, a post hoc analysis indicated that this heterogeneity in treatment did not affect the primary clinical outcome. This finding aligns with the cumulative evidence from randomized clinical studies, suggesting limited clinical value in additional ablation beyond PVI^5,39,40^. One potential explanation for the higher rate of ablation lines in the investigational arm could be the ease of use of the investigational catheter, which may facilitate physicians’ ablation strategies by making it easier to deliver the ablation. However, this hypothesis warrants further investigation in dedicated clinical studies specifically designed and powered to compare different ablation strategies for treating persistent AF using lattice-tip technology.

The SPHERE Per-AF trial demonstrated that, for patients with persistent AF resistant to anti-arrhythmic drugs, using an all-in-one high-density mapping and ablation catheter with a dual-energy ablation system is non-inferior to the conventional standard of care.

## Methods

### Trial design

SPHERE Per-AF (NCT05120193) was a pivotal, multicenter, randomized, single-blind, non-inferiority trial. The trial protocol is available in the Supplementary Information. The trial was funded by the manufacturer of the investigational mapping and ablation device, Affera, Inc. (later acquired by Medtronic). The trial received approval from the US Food and Drug Administration (FDA) and from the institutional review board at each participating center and was conducted in accordance with the principles of the Declaration of Helsinki.

The study’s design was developed by the sponsor, incorporating suggestions from several of the authors as well as the FDA. An autonomous board responsible for data and safety monitoring supervised participant safety and the execution of the trial, and an independent clinical events committee that was blinded to the randomization evaluated all outcomes of clinical significance. For evaluations of rhythm monitoring and brain MRI, independent core laboratories, blinded to the mapping and ablation platform, were used.

Data collection and monitoring for the trial were carried out by the sponsor, which also conducted the outcome analyses in line with the statistical methods. All study measurements were taken from distinct samples, with each trial participant as an independent sample. The Statistical Analysis Plan is available in the Supplementary Information. The authors were granted complete access to all data and analyses. The first draft of this manuscript was written by the first author, with subsequent reviews and edits by the other authors. Although the sponsor contributed suggestions, the final decision on the content of the manuscript rested with the first author. The authors collectively vouch for the data’s accuracy and completeness as well as the trial’s adherence to the established protocol.

### Study participants

All study participants provided written informed consent. Adults aged 18–80 years experiencing symptomatic persistent AF and who were refractory or intolerant to at least one class I or class III anti-arrhythmic drug were eligible for enrollment. Major inclusion and exclusion criteria are available in Extended Data Table 1; for a full list of inclusion and exclusion criteria, refer to the protocol. To ensure operator familiarity with the investigational mapping and ablation technology, each participating center was permitted to treat up to two roll-in patients. Subsequent patients were randomly assigned in a 1:1 ratio to undergo catheter-based ablation using either the lattice-tip or conventional radiofrequency technology. Randomization was completed via an electronic data capture system, where randomization was blocked and stratified by site and by enrollment in a neurological substudy. Randomized patients were blinded to their procedural assignment. An exploratory neurological substudy examined the effects of each treatment group on silent neurological events, as assessed by brain MRI (including diffusion-weighted imaging (DWI) and FLAIR sequences) and cognitive tests. Imaging targeted 24–48 h after the ablation procedure. Follow-up imaging was performed on day 90 in patients with post-procedure acute ischemia. A total of 23 centers across three countries (United States, Czech Republic and Israel) participated in the trial. The list of participating centers and investigators is provided in Supplementary Table 1 of the Supplementary Information.

### Interventions

The investigational technology includes a lattice-tip catheter (Sphere-9 catheter, Medtronic) with a compatible proprietary electro-anatomical mapping system (Affera Mapping and Ablation System, Medtronic) as previously described^22,23,21^. After creating a high-density electro-anatomical map of the left atrium, the same lattice-tip catheter was used for ablation, using either radiofrequency or pulsed field energies. Radiofrequency ablation applications were delivered in a temperature-controlled mode with an application duration of 5 s, with a target surface temperature of 73 °C and a current limit varying between 80% and 90%. The PFA applications consisted of a train of microsecond-scale pulses delivered for 4 s^22,23,21^. Operators were instructed to use pulsed field energy on the posterior wall, around the left inferior pulmonary vein and near the phrenic nerve but had discretion to use either type of energy in other areas.

In the control arm, operators employed a commercially available technology comprising an electro-anatomical mapping system (Carto 3, Biosense Webster), a multi-electrode mapping catheter and a contact force-sensing ablation catheter (THERMOCOOL SMARTTOUCH, Biosense Webster)^41^. Proprietary mapping catheters, including the Lasso, Pentaray and Octaray mapping catheters, were used for high-density mapping.

All procedures required high-density mapping performed with either the investigational or control mapping system. The ablation protocol was similar for both arms, requiring a wide-area circumferential PVI, with a procedural endpoint being a documented acute entrance block in each vein after a minimum 20-min observation period or infusion of adenosine or isoproterenol. Cavo-tricuspid isthmus linear ablation was required in cases with documented typical right atrial flutter either before or during the procedure. Additional linear ablation was permitted for treating documented macro-reentrant tachycardias. Although empiric linear lesion sets were generally discouraged, operators retained the freedom to adhere to their standard of care for treating persistent AF. Assessing block across an ablation line was conducted using differential pacing maneuvers and activation mapping, in line with the operator’s standard approach. This method was consistently applied across both study groups. Operators pursued standard of care per their medical discretion for procedural strategies, such as transseptal puncture, esophageal management, use of fluoroscopy and intra-cardiac echocardiography.

### Follow-up

Patients were discharged on oral anti-coagulation according to standard guidelines. The use of class I or class III anti-arrhythmic drugs was allowed but recommended to be discontinued before the end of a 90-d blanking period. Patients were followed for 1 year with office visits at 1 month, 3 months, 6 months and 12 months. After a 90-d blanking period, trans-telephonic ECG monitoring was required at least monthly with additional transmissions triggered by symptoms. A 24-h Holter monitor was performed at 6 months and 12 months, and 12-lead ECGs were performed at 3 months, 6 months and 12 months. Quality of life was evaluated at baseline and 12 months using the SF-12v2 Health Survey and the AFEQT survey^42–44^. In a neurological assessment substudy to assess for silent cerebral lesions, brain MRI (including DWI and FLAIR sequences) was performed within 72 h after the ablation procedure.

### Endpoints

The pre-specified primary effectiveness endpoint was freedom from a composite of multiple failure modes, including failure to acutely isolate all targeted PVs and complete all left atrial ablation with the assigned study device during the index procedure; repeat ablation at any time after the index procedure; and, after a 3-month blanking period, documented occurrence of atrial tachyarrhythmia, escalation or initiation of class I or class III anti-arrhythmic drugs or cardioversion. Documented recurrence of AF, atrial tachycardia or atrial flutter was based on either (1) an episode ≥30 s in duration documented by ECG, trans-telephonic monitor or Holter monitor or (2) an episode covering an entire 12-lead ECG recording lasting at least 10 s. The study protocol included additional superiority testing contingent upon non-inferiority of the primary endpoints. Energy application time, elapsed treatment time, total procedure time and primary effectiveness were sequentially tested for superiority of the investigational device compared to the control device.

The pre-specified primary safety endpoint was a composite of pre-specified device-related or procedure-related serious adverse events, including death, atrio-esophageal fistula, stroke, myocardial infarction, cardiac tamponade/perforation, PV stenosis, phrenic nerve paralysis, transient ischemic attack, thromboembolism, major vascular access complications/bleeding, heart block, gastroparesis, severe pericarditis or new or extended hospitalization for a cardiovascular or pulmonary adverse event. Adverse events were determined as serious if they (1) led to death; (2) led to serious deterioration in the health of the patient (including life-threatening illness or injury, permanent impairment of a body structure or function, >24-h hospitalization, chronic disease or medical or surgical intervention to prevent injury or permanent impairment of a body structure or function); and (3) led to fetal distress/death or a congenital abnormality or birth defect. Hospitalizations for pre-existing conditions or procedures without serious deterioration in health were not defined as serious. All primary adverse events were pre-specified, and their severity and association with the device or procedure were adjudicated by an independent clinical events committee. Pre-specified secondary effectiveness and performance endpoints included assessment of changes in quality of life, use of anti-arrhythmic drugs during the effectiveness evaluation period, procedure times, fluoroscopy time and ablation lesion sets delivered. All other endpoints were based on post hoc analyses.

### Statistical analysis

This trial aimed to assess for non-inferiority of the investigational device safety and effectiveness compared to the control device. To achieve power greater than 80% for testing each primary endpoint using the Farrington–Manning method, a sample size of 350 evaluable patients (175 per arm) was required for the primary analysis cohort (that is, randomized and treated patients), with assumed underlying rate of 8%, non-inferiority margin of 8% and one-sided alpha of 0.05 for the primary safety endpoint and assumed underlying rate of 60%, non-inferiority margin of 15% and one-sided alpha of 0.025 for the primary effectiveness endpoint. A total of 410 randomized patients was planned based on a conservative 15% attrition estimate. Trial success was defined by demonstrating both non-inferiority of the primary safety endpoint and non-inferiority of the primary effectiveness endpoint based on binomial proportions using the Farrington–Manning method. Contingent upon trial success, secondary sequential testing with an overall one-sided alpha of 0.025 was performed on a pre-specified set of endpoints to further examine superiority of the investigational device compared to the control device.

Quantitative variables were summarized using standard descriptive statistics, including number of non-missing observations, mean and s.d. Categorical variables were summarized using classical frequency statistics: number of non-missing observations, frequency and percentage by category. Kaplan–Meier analysis of the freedom from primary effectiveness failure events (including atrial tachyarrhythmia recurrence) was performed along with the log-rank test. Statistical analyses were performed using the SAS version 9.4 software package (SAS Institute).

### Reporting summary

Further information on research design is available in the Nature Portfolio Reporting Summary linked to this article.

## Online content

Any methods, additional references, Nature Portfolio reporting summaries, source data, extended data, supplementary information, acknowledgements, peer review information; details of author contributions and competing interests; and statements of data and code availability are available at 10.1038/s41591-024-03022-6.

### Supplementary information

Supplementary Information Supplementary Tables 1 and 2, Clinical Investigation Plan and Statistical Analysis Plan.

Reporting Summary

## Data Availability

All supporting data are available within the article and the Supplementary Information. Source data will not be shared due to patient privacy and informed consent, including the potential for release of protected health information.

## Extended data

**Extended Data Table 1:** Major inclusion and exclusion criteria

**Inclusion criteria** ^**a**^ 1. Symptomatic Per-AF documented by • (1) a physician’s note indicating symptoms consistent with AF sustained longer than 7 d but shorter than 12 months; AND either • (2a) a 24-h Holter documenting continuous AF within the past year OR • (2b) two ECGs (from any form of rhythm monitoring, including consumer devices) taken at least 7 d apart within the past year, each showing continuous AF. 2. Failure or intolerance of at least one class I or class III anti-arrhythmic drug. 3. Suitable candidate for catheter ablation. 4. Adults aged 18–80 years.
**Exclusion criteria** ^**a**^ 1. Continuous AF lasting for 12 months or longer. 2. AF secondary to electrolyte imbalance, thyroid disease, acute alcohol intoxication or other reversible or non-cardiac cause. 3. Previous left atrial ablation or surgical procedure. 4. Valvular cardiac surgical/percutaneous procedure. 5. Any cardiac procedure within the 90 d before the initial procedure. 6. Coronary artery bypass graft procedure within the 6 months before the initial procedure. 7. Presence of a permanent pacemaker or implantable cardiac defibrillator. 8. Documented thromboembolic event within 6 months before the initial ablation procedure. 9. Documented left atrial thrombus on imaging. 10. Body mass index > 40 kg m^−2^. 11. Left atrial diameter > 55 mm (anterioposterior). 12. Left ventricular ejection fraction < 35%. 13. Uncontrolled heart failure or New York Heart Association class III or class IV heart failure. 14. Moderate to severe mitral valve stenosis or severe mitral regurgitation. 15. Renal failure requiring dialysis.

^a^ For a full list of inclusion and exclusion criteria, refer to the full protocol.

**Extended Data Table 2:** Reasons for premature study exit of investigational and control patients before index ablation

Randomization	Reason for early exit	Reason details
Investigational	Screening failure after assignment (did not meet eligibility criteria)	Exclusion no. 31. Significant congenital anomaly or medical problem that, in the opinion of the investigator, would preclude enrollment in this study or compliance with follow-up requirements or would impact the scientific soundness of the clinical trial results
Investigational	Screening failure after assignment (did not meet eligibility criteria)	Inclusion no. 5. Willing and able to comply with all baseline and follow-up evaluations for the full length of the study
Investigational	Screening failure after assignment (did not meet eligibility criteria)	Inclusion no. 5. Willing and able to comply with all baseline and follow-up evaluations for the full length of the study
Investigational	Screening failure after assignment (did not meet eligibility criteria)	Exclusion no. 18. Left ventricular ejection fraction < 35%
Investigational	Screening failure after assignment (did not meet eligibility criteria)	Inclusion no. 5. Willing and able to comply with all baseline and follow-up evaluations for the full length of the study
Investigational	Excluded (no mapping/ablation procedure with study device)	Excluded due to lack of study device/catheter
Investigational	Subject death	Hemorrhage intracranial
Control	Screening failure after assignment (did not meet eligibility criteria)	Inclusion no. 5. Willing and able to comply with all baseline and follow-up evaluations for the full length of the study
Control	Excluded (no mapping/ablation procedure with study device)	Excluded due to lack of study device
Control	Excluded (no mapping/ablation procedure with study device)	Excluded due to lack of study device
Control	Excluded (no mapping/ablation procedure with study device)	Excluded due to lack of study device
Control	Excluded (no mapping/ablation procedure with study device)	Excluded due to lack of study device

**Extended Data Table 3:** Compliance rates

Compliance category	Investigational arm (*n* = 212)	Control arm (*n* = 208)	Total cohort (*n* = 420)
Follow-up visits completed	1,170/1,211 (96.7%)	1,133/1,174 (96.5%)	2,303/2,385 (96.6%)
Holter monitoring	352/422 (83.4%)	344/406 (84.7%)	696/828 (84.1%)
12-lead ECG ^a^	547/634 (86.3%)	515/613 (84.0%)	1,062/1,247 (85.2%)
Trans-telephonic monitoring			
Compliance rate	1,753/1,875 (93.5%)	1,664/1,831 (90.9%)	3,417/3,706 (92.2%)
Monthly rate	2.2 ± 2.6	1.9 ± 1.6	2.1 ± 2.1

^a^ If the visit was a telehealth visit, trans-telephonic monitoring was performed in lieu of a 12-lead ECG and was included in the count.

**Extended Data Table 4:** Primary effectiveness endpoint failure modes

Parameter	Investigational arm (*n* = 210)	Control arm (*n* = 202)
Primary effectiveness endpoint success	155 (73.8%)	133 (65.8%)
Primary effectiveness endpoint failure^a^	55 (26.2%)	69 (34.2%)
Inability to isolate all targeted PVs during the index procedure	0 (0.0%)	0 (0.0%)
Any left atrial ablation done with non-assigned study device during the index procedure	0 (0.0%)	2 (1.0%)
Any repeat ablation or surgery for AF/AT/AFL recurrence after the index procedure	10 (4.8%)	17 (8.4%)
Direct current cardioversion for AF/AT/AFL recurrence during the effectiveness evaluation period	13 (6.2%)	13 (6.4%)
Documented AF/AT/AFL recurrence during the effectiveness evaluation period	49 (23.3%)	55 (27.2%)
Class I/III anti-arrhythmic drug dose increase from the historic maximum ineffective dose or initiation of new class I/III anti-arrhythmic drug during the effectiveness evaluation period	8 (3.8%)	15 (7.4%)

^a^ Failure modes are not mutually exclusive because the same patient can experience multiple failure modes. All failure modes are presented in this table.

**Extended Data Table 5:** All adverse events related or possibly related to procedure or device

Adverse event preferred term^a^	No. of events (no. of patients, % patients)	
	Investigational arm (*n* = 212)	Control arm (*n* = 208)
Total	31 (29, 13.7%)	43 (32, 15.4%)
Abdominal pain	1 (1, 0.5%)	0 (0, 0.0%)
Anesthetic complication	0 (0, 0.0%)	1 (1, 0.5%)
Arteriovenous fistula	0 (0, 0.0%)	1 (1, 0.5%)
Atrial fibrillation	0 (0, 0.0%)	1 (1, 0.5%)
Body temperature increased	0 (0, 0.0%)	2 (2, 1.0%)
Bradycardia	2 (2, 0.9%)	0 (0, 0.0%)
Chest discomfort	0 (0, 0.0%)	1 (1, 0.5%)
Chest pain	0 (0, 0.0%)	1 (1, 0.5%)
Chronic obstructive pulmonary disease	1 (1, 0.5%)	0 (0, 0.0%)
Cough	0 (0, 0.0%)	1 (1, 0.5%)
Cyst	0 (0, 0.0%)	1 (1, 0.5%)
Esophageal mucosa erosion	0 (0, 0.0%)	1 (1, 0.5%)
Fatigue	2 (2, 0.9%)	0 (0, 0.0%)
Hypertensive urgency	1 (1, 0.5%)	0 (0, 0.0%)
Hematuria	0 (0, 0.0%)	1 (1, 0.5%)
Hemoptysis	1 (1, 0.5%)	0 (0, 0.0%)
Hiatal hernia with gastritis	1 (1, 0.5%)	0 (0, 0.0%)
Hypervolemia	4 (4, 1.9%)	8 (8, 3.8%)
Hypotension	0 (0, 0.0%)	1 (1, 0.5%)
Hypoxia	0 (0, 0.0%)	1 (1, 0.5%)
Lip injury	1 (1, 0.5%)	0 (0, 0.0%)
Myocardial infarction	0 (0, 0.0%)	1 (1, 0.5%)
Non-cardiac chest pain	1 (1, 0.5%)	0 (0, 0.0%)
Ocular discomfort	0 (0, 0.0%)	1 (1, 0.5%)
Pericardial effusion/constriction^b^	1 (1, 0.5%)	0 (0, 0.0%)
Pericarditis	0 (0, 0.0%)	3 (3, 1.4%)
Pharyngitis	1 (1, 0.5%)	0 (0, 0.0%)
Phlebitis	1 (1, 0.5%)	0 (0, 0.0%)
Phrenic nerve injury^c^	2 (2, 0.9%)	0 (0, 0.0%)
Pleuritic pain	0 (0, 0.0%)	1 (1, 0.5%)
Pneumonia	0 (0, 0.0%)	1 (1, 0.5%)
Productive cough	0 (0, 0.0%)	1 (1, 0.5%)
Rash	2 (2, 0.9%)	0 (0, 0.0%)
Raynaud’s phenomenon	0 (0, 0.0%)	1 (1, 0.5%)
Sepsis	2 (2, 0.9%)	1 (1, 0.5%)
Skin irritation	0 (0, 0.0%)	1 (1, 0.5%)
Vascular access site hematoma	3 (3, 1.4%)	3 (3, 1.4%)
Vascular access site hemorrhage	2 (2, 0.9%)	3 (3, 1.4%)
Vascular access site irritation	0 (0, 0.0%)	1 (1, 0.5%)
Vascular access site laceration	0 (0, 0.0%)	1 (1, 0.5%)
Vascular access site mass	0 (0, 0.0%)	1 (1, 0.5%)
Visual impairment	0 (0, 0.0%)	2 (2, 1.0%)
Weight increased	2 (2, 0.9%)	0 (0, 0.0%)

^a^ Results of the neurological substudy are reported separately in Extended Data Table 8.

^b^ A delayed effusion occurred at day 264 after index procedure in a patient who had a history of large pericardial effusion before the ablation.

^c^ Two transient phrenic nerve injuries occurred in the investigational arm, and both patients documented full resolution based on a sniff test.

**Extended Data Table 6:** Quality of life

Parameter^a^	Visit	Investigational arm	Control arm
AFEQT^42,43^^b^	Baseline	66.7 ± 21.5	68.7 ± 20.2
	12-month visit	89.0 ± 13.7	90.9 ± 13.3
	Change	22.3 ± 19.5	22.2 ± 19.3
SF-12v2 Physical Component Score^c^	Baseline	45.5 ± 8.5	44.9 ± 8.9
	12-month visit	50.2 ± 8.4	49.6 ± 8.9
	Change	4.7 ± 7.6	4.7 ± 8.5
SF-12v2 Mental Component Score^44–47^^c^	Baseline	51.1 ± 9.3	51.0 ± 9.1
	12-month visit	54.3 ± 8.0	55.3 ± 7.3
	Change	3.2 ± 8.1	4.3 ± 8.8

^a^ Numbers presented are mean ± s.d.

^b^ Data available for 207 patients (investigational) and 194 patients (control).

^c^ Data available for 196 patients (investigational) and 184 patients (control).

**Extended Data Table 7:** Primary effectiveness endpoint (PEE) assessment based on presence or absence of linear lesions

Parameter	Treatment arm	PEE no. of successes / no. of patients (%)		Heterogeneity test *P* value
		Yes	No	
Mitral lines	Control	14/22 (63.6%)	119/180 (66.1%)	0.79
	Investigational	50/71 (70.4%)	105/139 (75.5%)	
Left atrial posterior/floor/roof	Control	86/133 (64.7%)	47/69 (68.1%)	0.14
	Investigational	147/196 (75.0%)	8/14 (57.1%)	

**Extended Data Table 8:** Neurological assessment substudy: summary of cerebral ischemia

Cohort	Investigational arm^a^	Control arm^b^
Total number of patients	37	35
Acute ischemia with FLAIR hyperintensity (%)^c^	3 (8.1%)	2 (5.7%)
Acute ischemia without FLAIR hyperintensity (%)^c^	3 (8.1%)	2 (5.7%)

^a^ Represents patients enrolled across 12 centers.

^b^ Represents patients enrolled across 10 centers.

^c^ Events are mutually exclusive.

## Code availability

No custom code was used.
