# Supplementary material for: Dual-energy lattice-tip ablation system for persistent atrial fibrillation: a randomized trial
Source: Nat Med. 2024 May 17;30(8):2303–10. doi: 10.1038/s41591-024-03022-6 (PMC11333282; doi:10.1038/s41591-024-03022-6)
Supplement: Supplementary file 1 — Supplementary Tables 1 and 2, Clinical Investigation Plan and Statistical Analysis Plan. [file 41591_2024_3022_MOESM1_ESM.pdf]

nature medicine

Article https://doi.org/10.1038/s41591-024-03022-6

Dual-energy lattice-tip ablation system for persistent atrial fibrillation: a randomized trial

In the format provided by the
authors and unedited

Supplementary Table 1: SPHERE Per-AF Consortium Investigators

The authors would like to thank the investigators in the following table, which is arranged alphabetically by site name.

Site Name and Address	Principal Investigator*	Sub-Investigators
AdventHealth Cardiovascular Research Institute<br>601 E Rollins St Mailbox 99<br>Orlando, FL 32803; USA	Usman Siddiqui, MD	George Monir, MD
Arrythmia Institute at Grandview<br>3686 Grandview Pkwy, Ste 720<br>Birmingham, AL 35243; USA	Anil Rajendra, MD<br>Jose Osorio, MD	Gustavo Morales, MD<br>Judson Colley, MD, MPH
Arrhythmia Research Group<br>201 East Oak Avenue<br>Jonesboro, AR 72401; USA	Devi Nair, MD	(none)
Banner – University Medical Center Phoenix<br>1111 E McDowell Rd Fl 1 RM 0100825<br>Phoenix, AZ 85006; USA	Roderick Tung, MD	Wilber Su, MD
Cleveland Clinic<br>9500 Euclid Ave<br>Cleveland, OH 44195; USA	Tyler Taigen, MD	Ayman Hussein, MD<br>Bryan Baranowksi, MD<br>Dan Cantillon, MD<br>Mohamed Kanj, MD<br>Oussama Wazni, MD, MBA<br>Roy Chung, MD<br>Walid Saliba, MD
Doylestown Hospital<br>599 W State St Ste 200<br>Doylestown, PA 18901; USA	Robert Sangrigoli, MD	John Harding, MD
Institut Klinicke a Experimentální Medicíny<br>Vídeňská 1958<br>140 21 Praha 4<br>Czech Republic	Josef Kautzner, MD, PhD	Dan Wichterle, MD, PhD<br>Predrag Stojadinovic, MD<br>Petr Peichl, MD, PhD
Maimonides Medical Center<br>4802 10th Avenue<br>Brooklyn, NY 11219; USA	Yisachar Greenberg, MD<br>Felix Yang, MD	Rohan Patel, MD
Massachusetts General Hospital<br>55 Fruit St<br>Boston, MA 02114; USA	Moussa Mansour, MD	Chee Yuan Ng, MD<br>Nathan Van Houzen, MD
MedStar Washington Hospital Center<br>110 Irving St NW Ste 5A-12<br>Washington, DC 20010; USA	Sung Lee, MD	Manish Shah, MD<br>Sarfraz Durrani, MD

Site Name and Address	Principal Investigator*	Sub-Investigators
Mount Sinai Medical Center<br>5 E 98th St<br>New York, NY 10029; USA	Srinivas Dukkipati, MD	Jacob Koruth, MD<br>Vivek Reddy, MD<br>William Whang, MD<br>Mohit Turagam, MD<br>Marc Miller, MD<br>Abhishek Maan, MD
NCH Heart Institute<br>700 2ND Avenue North<br>Suite 301<br>Naples, FL 34102; USA	Dinesh Sharma, MD, MPH	David Axline, MD
Nemocnice Ceske Budejovice<br>B. Němcové 585/54<br>370 01 Ceske Budejovice<br>Czech Republic	Alan Bulava, MD, PhD	Jiri Hanis, MD
Nemocnice Na Homolce<br>Roentgenova 37<br>150 00 Praha 5<br>Czech Republic	Petr Neuzil, MD, PhD	Jan Petru, MD<br>Jan Skoda, MD<br>Libor Dujka, MD<br>Tereza Stastna, MD<br>Veronika Svecova, MD<br>Branislav Chudiak, MD<br>Martin Tousek, MD<br>Pavel Hala, MD
Northwell Health Lenox Hill Hospital<br>100 East 77th St<br>New York, NY 10075; USA	Stavros Mountantonakis, MD, MBA	Haisam Ismail, MD<br>Mark Abrams, MD<br>Moussa Saleh, MD
Pacific Heart Institute<br>2001 Santa Monica Blvd Ste 280W<br>Santa Monica, CA 90404; USA	Shephal Doshi, MD	Sarina van der Zee, MD<br>Stephen Tang, MD
Sentara Norfolk General Hospital<br>600 Gresham Drive<br>Norfolk, VA 23507; USA	Erich Kiehl, MD	Jon Grammes, DO<br>Olusegun Olusesi, MD<br>Philip Gentlesk, MD
Shamir Medical Center Israel<br>Zrifin 6093000<br>Israel	Aharon Medina, MD	Elad Anter, MD<br>Michael Barkagan, MD<br>Vladimir Vasilenko, MD
Texas Cardiac Arrhythmia Research Foundation<br>3000 N. IH-35<br>Ste 705<br>Austin, TX 78705; USA	Andrea Natale, MD	David Burkhardt, MD<br>Joe Gallinghouse, MD

Site Name and Address	Principal Investigator*	Sub-Investigators
The OhioHealth Research Institute<br>3545 Olentangy River Rd. Suite 301<br>Columbus, OH 43214; USA	Anish Amin, MD	Auroa Badin, MD
The Ohio State University Wexner Medical Center<br>473 W. 12th Ave Suite 155<br>Columbus, OH 43210; USA	John Hummel, MD<br>Toshimasa Okabe, MD, MS	Emile Daoud, MD<br>Mahmoud Houmsse, MD
UPMC Pinnacle Harrisburg Campus<br>1000 North Front Street<br>Wormleysburg, PA 17043; USA	Chinmay Patel, MD	Heather Metzger, MD<br>Jessica Root, MD
Vanderbilt Medical Center<br>1215 21ST Avenue South<br>MCE 5th Floor, South Tower<br>Nashville, TN 27232; USA	Travis Richardson, MD	Christopher Ellis, MD<br>Sharon Shen, MD

*Multiple principal investigators are listed if an investigator left an institution during the trial and another investigator at that site became the principal investigator.

Supplementary Table 2: SPHERE Per-AF Committees

**Steering/ Publication Committee**
Elad Anter, MD - Chair
Devi Nair, MD
Dinesh Sharma, MD
Moussa Mansour, MD
Tyler Taigen, MD
Petr Neuzil, MD
Erich Kiehl, MD
Josef Kautzner, MD, Ph.D.
Jose Osorio, MD
Stavros Mountantonakis, MD
Andrea Natale, MD
Anish Amin, MD
Usman Siddiqui, MD
Vivek Reddy, MD
**Clinical Events Committee**
James Daubert, MD - Chair
Ferdinand Hui, MD
Harry Hixson, MD
Matthew Reynolds, MD, MSc
Steven Furer, MD
**Data Monitoring Committee**
Bruce Koplan, MD, M.P.H. - Chair
Helen Parise, MS, ScD
Jared Bunch, MD
William Gray, MD

TREATMENT OF PERSISTENT ATRIAL FIBRILLATION WITH THE SPHERE-9 MAPPING AND ABLATION CATHETER AND THE AFFERA MAPPING AND ABLATION SYSTEM

CLINICAL INVESTIGATION PLAN

Clinical Investigation Plan	CP-00009
Version and Date	Revision G<br>03 June, 2022
Investigational Device	Sphere-9™ Mapping and Ablation Catheter with the Affera Mapping and Ablation System
Description	Prospective, multicenter, randomized interventional study to evaluate the safety and effectiveness of the Sphere-9 Mapping and Ablation Catheter with the Affera Mapping and Ablation System for the treatment of symptomatic persistent atrial fibrillation (PerAF) refractory or intolerant to drugs using radiofrequency (RF) and pulsed field (PF) ablation
Sponsor and Investigational Device Manufacturer	Affera, Inc.<br>320 Nevada St, Suite 401<br>Newton, MA 02460<br>USA

DISCLOSURE STATEMENT

This document contains information that is confidential and proprietary to Affera, Inc. This information is being provided to you solely for the purpose of evaluating or conducting a clinical study for Affera. You may disclose the contents of this document only to study personnel under your supervision who need to know the contents for this purpose and to your Institutional Review Board (IRB) or Ethics Committee (EC). Otherwise, the contents of this document may not be disclosed without the prior authorization from Affera. The foregoing shall not apply to disclosure required by governmental regulations or laws. Any supplemental information that may be added to this document also is confidential and proprietary to Affera and must be kept in confidence in the same manner as the contents of this document.

INVESTIGATOR AGREEMENT

I have read this Clinical Investigation Plan and agree to adhere to the requirements. I will provide copies of this Clinical Investigation Plan and all pertinent information to all site personnel involved in this study. I will discuss this material with them and ensure they are fully informed regarding the investigational device and the conduct of the study.

I agree to conduct the study as outlined in the Clinical Investigation Plan, in accordance with the signed clinical study agreement, and according to the ethical principles stated in the latest version of the Declaration of Helsinki (2013), the applicable guidelines for good clinical practices, 21 CFR parts 11, 50, 54, 56, and 812, ISO 14155:2011 (Clinical Investigation of Medical Devices for Human Subjects – Good Clinical Practice), or the applicable local and international regulations, whichever provides the greater protection of the individual.

I agree to actively enroll subjects into the study. I agree to provide all information requested in the Case Report Forms provided to me by the Sponsor in a manner to assure completeness, legibility, and accuracy.

I agree that all information provided to me by the Sponsor, including pre-clinical data, protocols, Case Report Forms, and any verbal and written information, will be kept strictly confidential and confined to the clinical personnel involved in conducting the study. It is recognized that this information may be relayed in confidence to the Institutional Review Board (IRB) or Ethics Committee (EC).

I will not provide protocols, reports, or information about the study or its progress to anyone who is not involved in the study other than the Sponsor, the IRB/EC, and relevant regulatory authorities. Any such submission will indicate that the material is confidential.

______________________________ __________________

Investigator Signature Date

____

Investigator's Name (print)

TABLE OF CONTENTS

1 CLINICAL INVESTIGATION SUMMARY 8
2 ABBREVIATIONS 15
3 INTRODUCTION 16
3.1 Background 16
3.1.1 Atrial Fibrillation 16
3.1.2 Demographic Profile of Atrial Fibrillation Patients 16
3.1.3 Ablation of Atrial Fibrillation 16
3.1.4 Safety of AF Ablation 17
3.1.5 Clinical Outcomes of AF Ablation 18
3.2 Rationale for Clinical Investigation 18
4 INVESTIGATIONAL DEVICE INFORMATION 20
4.1 Intended Use 20
4.2 Device Description 20
5 CONTROL DEVICE INFORMATION 24
5.1 Indications for Use 24
6 STUDY OBJECTIVES 25
6.1 Primary Study Objectives 25
6.2 Neurological Assessment Sub-Study Objective 25
7 STUDY ENDPOINTS 26
7.1 Primary Safety Endpoint 26
7.2 Primary Effectiveness Endpoint 26
7.3 Secondary Endpoints 28
7.4 Neurological Assessment Sub-Study 29
8 STUDY DESIGN 30
8.1 Population 30
8.2 Clinical Sites 30
8.3 Study Duration 30
8.4 Inclusion Criteria 30
8.5 Exclusion Criteria 30
8.6 Roll-In Phase 32
8.7 Neurological Assessment Sub-Study 32
8.8 Subject Accountability 32
8.8.1 Screening Failure 32
8.8.2 Excluded Subjects 32
8.8.3 Discontinued Subjects 33
8.8.4 Treated Subjects 33
8.8.5 Withdrawal and Lost to Follow-Up 33
8.8.6 Completed Subjects 33
9 STUDY PROCEDURES 34

9.1 Patient Screening 34
9.2 Informed Consent 34
9.3 Roll-In 34
9.4 Randomization 35
9.5 Pre-Procedure/Baseline Assessments 35
9.6 Anticoagulation 36
9.6.1 Systemic Anticoagulation Prior to Ablation 36
9.6.2 Intraprocedural Anticoagulation 36
9.6.3 Early Postprocedural Anticoagulation 36
9.7 Thrombus Screening 36
9.8 Ablation Procedure 37
9.8.1 Pulmonary Vein Isolation 38
9.8.2 Additional Ablation 38
9.8.3 Data Collection 39
9.8.4 Investigational Device Use 39
9.8.5 Control Device Use 40
9.9 Neurological Assessment Sub-Study 41
9.10 Repeat Ablation 43
9.11 Antiarrhythmic Drugs (AADs) 43
9.12 Transtelephonic Monitoring (TTM) 43
9.13 Holter Monitoring (HM) 44
9.14 12-Lead ECG Recordings 44
9.15 Follow-Up Visits 44
9.15.1 Pre-Discharge Visit 44
9.15.2 Day 10 Telephone Call (7-13 days) 45
9.15.3 Day 30 Office/Hybrid Visit (14-42 days) 45
9.15.4 Day 75 Telephone Call (60-90 days) 46
9.15.5 Day 90 Office/Telehealth Visit (76-104 days) 46
9.15.6 Day 180 Office/Telehealth Visit (150-210 days) 46
9.15.7 Day 360 Office/Telehealth Visit (330-390 days) 47
9.15.8 Unscheduled Visits 47
10 ADVERSE EVENTS AND DEVICE DEFICIENCIES 51
10.1 Definitions 51
10.1.1 Adverse Events 51
10.1.2 Serious Adverse Events 51
10.1.3 Adverse Device Effects 51
10.1.4 Device Deficiency 52
10.2 Assessment of Adverse Events 52
10.3 Reporting Requirements 54
11 STATISTICAL METHODS 60
11.1 Primary Analyses 60
11.1.1 Poolability across Sites 61
11.1.2 Sensitivity Analysis 61

11.1.3 Subgroup Analyses 62
11.1.4 Sample Size 62
11.2 Secondary Analyses 62
11.3 Additional Analyses 63
11.4 Neurological Assessment Sub-Study 63
12 RISK ANALYSIS 65
12.1 Potential Benefits 65
12.2 Potential Risks 65
12.3 Minimization of Risks 68
13 STUDY CONDUCT 69
13.1 Ethics 69
13.2 Institutional Review Board / Ethics Committee 69
13.3 US FDA and Local Competent Authority 69
13.4 Informed Consent 69
13.5 Confidentiality and Data Protection 70
13.6 Insurance 70
13.7 Data Quality Assurance 71
13.7.1 Site Selection 71
13.7.2 Training 71
13.7.3 Site Initiation 71
13.7.4 Case Report Forms 71
13.7.5 Documentation 72
13.7.6 Data Monitoring 72
13.7.7 Device Accountability 73
13.7.8 Data Management 73
13.7.9 Core Labs 73
13.8 Protocol Deviations 74
13.9 Study Audits 74
13.10 Record Keeping 75
13.10.1 Data Retention 76
13.11 Reports 76
13.11.1 Investigator Reports 76
13.11.2 Publication 77
14 STUDY OVERSIGHT 78
14.1 Study Advisory Committee 78
14.2 Investigator Responsibilities 78
14.3 Sponsor Responsibilities 78
14.4 Data and Safety Monitoring Board 79
14.5 Clinical Events Committee 79
14.6 Protocol Amendments 80
14.7 Suspension or Discontinuation of the Study 80
15 REFERENCES 81

TABLE OF FIGURES

Figure 1. Sphere-9 Mapping and Ablation Catheter. 21
Figure 2. Affera Ablation System 22
Figure 3. Study procedures flowchart. 50

TABLE OF TABLES

Table 1. Affera Mapping System components. 21
Table 2. Affera Ablation System components. 22
Table 3. Recommended RF ablation settings for Sphere-9 Catheter. 40
Table 4. Recommended PF ablation settings for Sphere-9 Catheter. 40
Table 5. Recommended RF ablation settings for THERMOCOOL SMARTTOUCH SF® catheter. 41
Table 6. Schedule of evaluations and visits. 48
Table 7. Neurological Assessment Sub-Study additional/modified follow-up evaluations. 49
Table 8. Adverse event severity definitions. 53
Table 9. Adverse event expectedness definitions. 53
Table 10. Adverse event relationship to the device or procedure definitions. 54
Table 11. Adverse event reporting timelines. 54
Table 12. Anticipated adverse events. 56
Table 13. Primary adverse event definitions. 58
Table 14. Subject cohorts for statistical analysis. 60

1 Clinical Investigation Summary

**Study Title**	Treatment of Persistent Atrial Fibrillation with the Sphere-9 Mapping and Ablation Catheter with the Affera Mapping and Ablation System
**Investigational Device**	Sphere-9™ Mapping and Ablation Catheter<br>Affera Mapping System<br>Affera Ablation System
**Sponsor and Manufacturer**	Affera, Inc.<br>320 Nevada St, Suite 401<br>Newton, MA 02460<br>USA
**Control Device**	THERMOCOOL SMARTTOUCH® SF Catheter<br>SMARTABLATE® System<br>CARTO® 3 System
**Study Design**	This is a prospective, multicenter, randomized clinical evaluation of the Sphere-9 Mapping and Ablation Catheter with the Affera Mapping and Ablation System. Subjects will be randomly assigned 1:1 to receive treatment with either the Sphere-9 Mapping and Ablation Catheter and the Affera Mapping and Ablation System (investigational device) or the THERMOCOOL SMARTTOUCH® SF radiofrequency ablation catheter (control device). Subjects will be blinded to treatment assignment.
**Objectives**	The objectives of this study are to evaluate the safety and effectiveness of the Sphere-9 Mapping and Ablation Catheter with the Affera Mapping and Ablation System for the treatment of symptomatic persistent atrial fibrillation (PerAF) refractory or intolerant to drugs using radiofrequency (RF) and pulsed field (PF) ablation. This study aims to demonstrate safety and effectiveness non-inferiority compared to the control device.
**Clinical Sites**	Subjects will be enrolled at up to 35 sites in the United States (US), Canada, Europe, Australia, and/or Israel, with up to approximately 50% of subjects enrolled outside the US.

**Population**	Patients with symptomatic persistent atrial fibrillation (PerAF) refractory or intolerant to drugs and planned for catheter ablation
**Enrollment**	An overall maximum of 480 subjects will be enrolled, including a planned total of 410 randomized subjects and up to 70 Roll-In subjects as described below.<br><br>To ensure that investigators have adequate training with the investigational device, each site will be permitted to use the investigational device in up to two enrolled non-randomized Roll-In subjects. Subjects in the Roll-In Cohort will be analyzed separately from the Primary Analysis Cohort and the Secondary Effectiveness Cohort.
**Primary Safety Endpoint**	The primary safety endpoint is the incidence of the following device- or procedure-related serious adverse events (SAEs) following the index ablation procedure:<br>Within 7 days:<br>• Death<br>• Myocardial infarction<br>• Phrenic nerve paralysis<br>• Transient ischemic attack (TIA)<br>• Stroke/cerebrovascular accident (CVA)<br>• Thromboembolism<br>• Major vascular access complications / bleeding<br>• Heart block<br>• Gastroparesis<br>• Severe pericarditis<br>• Hospitalization (initial and prolonged) due to cardiovascular or pulmonary AE‡<br>Within 30 days:<br>• Cardiac tamponade / perforation<br>Within 90 days:<br>• Atrio-esophageal fistula<br>Within 180 days:<br>• Pulmonary vein stenosis<br>‡ Excludes hospitalization due to AF/AFL/AT recurrence
**Primary Effectiveness Endpoint**	The primary effectiveness endpoint is freedom from documented recurrence of AF, atrial tachycardia (AT), or atrial flutter (AFL) based on electrocardiographic data through 12-month follow-up and excluding

	a 90-day blanking period. The following are considered primary effectiveness endpoint failures:<br>• Inability to isolate all targeted pulmonary veins during the index procedure.<br>• Ablation using devices other than the assigned study device for any left atrial ablation during the index procedure.<br>• Any repeat ablation, surgical ablation, or arrhythmia surgery for treatment of recurrent AF/AT/AFL after the index procedure.<br>• Documented AF/AT/AFL recurrence after the 90-day blanking period.<br>• Class I or III AAD dose increase from the historic maximum ineffective dose (prior to the index procedure) or initiation of a new Class I or III AAD for treatment of AF/AT/AFL after the 90-day blanking period.<br>• DC cardioversion for AF/AT/AFL after the 90-day blanking period.
**Secondary Endpoints**	**Secondary Safety Endpoints**<br>• Incidence of early onset (within 7 days of the index ablation procedure) serious adverse events (SAEs)<br>• Incidence of peri-procedural (>7 and ≤30 days) SAEs<br>• Incidence of late onset (>30 days) SAEs<br><br>**Secondary Effectiveness Endpoints**<br>• Changes in Quality of Life (QOL) using the Atrial Fibrillation Effect on Quality of Life (AFEQT) Questionnaire and the SF-12v2 health survey<br>• Rate of acute procedural success, defined as confirmation of entrance block in all targeted pulmonary veins<br>• Rate of pulmonary vein isolation on a per-vein basis<br>• Rate of acute block across linear ablations<br>• Use of antiarrhythmic drugs (AADs) during the effectiveness evaluation period<br>• Freedom from documented recurrence of symptomatic AF/AFL/AT through 12-month follow-up and excluding the 90-day blanking period<br>• Freedom from documented recurrence of symptomatic AF/AFL/AT off antiarrhythmic drug therapy through 12-month follow-up and excluding the 90-day blanking period<br><br>**Secondary Performance Endpoints**<br>• Investigator scoring of electroanatomical mapping

	• Total fluoroscopy time during the ablation procedure<br>• Procedure time, defined as the time elapsed from first venous access to last sheath removal<br>• Treatment time, defined as the time from the start of the first ablation delivery to the end of the last ablation delivery<br>• Total energy application time during the ablation procedure<br>• Ablation lesion sets delivered (ablation strategy)<br>• Ablation parameters per application<br>• Ablation energy type (RF or PF) used for each ablation lesion set
**Inclusion Criteria**	1. Symptomatic PerAF documented by<br>• (1) a physician's note indicating symptoms consistent with AF sustained longer than 7 days but less than 12 months;<br>AND either<br>• (2a) a 24-hour Holter documenting continuous AF within the past year<br>OR<br>• (2b) two electrocardiograms (from any form of rhythm monitoring, including consumer devices) taken at least 7 days apart within the past year, each showing continuous AF.<br>2. Failure or intolerance of at least one Class I or III anti-arrhythmic drug (AAD).<br>3. Suitable candidate for catheter ablation.<br>4. Adults aged 18 – 80 years.<br>5. Willing and able to comply with all baseline and follow-up evaluations for the full length of the study.<br>6. Willing and able to provide informed consent.
**Exclusion Criteria**	1. Continuous AF lasting for 12 months or longer.<br>2. AF secondary to electrolyte imbalance, thyroid disease, acute alcohol intoxication, or other reversible or non-cardiac cause.<br>3. Previous left atrial ablation or surgical procedure (including septal closure or left atrial appendage closure).<br>4. Valvular cardiac surgical/percutaneous procedure (e.g., ventriculotomy, atriotomy, and valve repair or replacement and presence of a prosthetic valve).<br>5. Any carotid stenting or endarterectomy.<br>6. Any cardiac procedure (surgical or percutaneous) or percutaneous coronary intervention within the 90 days prior to the initial procedure.<br>7. Coronary artery bypass graft (CABG) procedure within the 6 months prior to the initial procedure.

8. Awaiting cardiac transplantation or other cardiac surgery within the 12 months following the initial ablation procedure.
9. Presence of a permanent pacemaker, biventricular pacemaker, or any type of implantable cardiac defibrillator (with or without biventricular pacing function).
10. Documented thromboembolic event (stroke or transient ischemic attack) within 6 months (180 days) prior to the initial ablation procedure.
11. Documented left atrial thrombus on imaging.
12. History of blood clotting or bleeding abnormalities.
13. Any condition contraindicating chronic anticoagulation.
14. Myocardial infarction (MI) within the 3 months (90 days) prior to the initial procedure.
15. Body mass index >40 kg/m^2.
16. Left atrial diameter >55 mm (anterioposterior).
17. Diagnosed atrial myxoma.
18. Left ventricular ejection fraction (EF) < 35%.
19. Uncontrolled heart failure or NYHA Class III or IV heart failure.
20. Rheumatic heart disease.
21. Hypertrophic cardiomyopathy.
22. Unstable angina.
23. Moderate to severe mitral valve stenosis.
24. Severe mitral regurgitation (regurgitant volume $\geq$ 60 mL/beat, regurgitant fraction $\geq$ 50%, and/or effective regurgitant orifice area $\geq$ 0.40cm^2).
25. Primary pulmonary hypertension.
26. Significant restrictive or obstructive pulmonary disease or chronic respiratory condition.
27. Renal failure requiring dialysis.
28. History of severe Gastroesophageal Reflux Disease (GERD) requiring surgical and/or mechanical intervention.
29. Acute illness, active systemic infection, or sepsis.
30. Contraindication to both computed tomography and magnetic resonance angiography.
31. Significant congenital anomaly or medical problem that, in the opinion of the investigator, would preclude enrollment in this study or compliance with follow-up requirements or would impact the scientific soundness of the clinical study results.
32. Any woman known to be pregnant or breastfeeding, or any woman of childbearing potential who is not on a reliable form of birth regulation method or abstinence.
33. Current or anticipated participation in any other clinical study of a drug, device, or biologic during the duration of the study not pre-approved by the Sponsor.

	34. Presence of intramural thrombus, tumor, or other abnormality that precludes vascular access, catheter introduction, or manipulation.<br>35. Known drug or alcohol dependency.<br>36. Life expectancy less than 12 months.<br>37. Vulnerable subject (such as a prisoner or handicapped or mentally disabled person).
**Study Procedures**	**Index Ablation Procedure**<br>Wide antral circumferential ablation (WACA, i.e. adjacent ablation lesions encircling ipsilateral veins 1 – 2 cm outside of the vein ostium) must be used to isolate all targeted pulmonary veins. Entrance block will be confirmed in all targeted pulmonary veins after a 20-minute waiting period or provocative testing with adenosine or isoproterenol.<br><br>The following linear ablations are only required in order to treat documented macroreentrant tachycardias:<br>• Linear lesions between the tricuspid annulus and the inferior vena cava (cavotricuspid isthmus line)<br>• Linear lesions between the left and right pulmonary veins (roof line and/or posterior line), optionally including homogenization of the posterior wall of the left atrium<br>• Linear lesions between the mitral annulus and the left pulmonary veins (mitral isthmus line), with ablation permitted in the apposing coronary sinus.<br>Cavotricuspid isthmus linear ablation is required in cases with documented typical right atrial flutter either prior to or during the procedure. Empirical atrial linear ablation is discouraged.<br><br>Additional ablation beyond these lesion sets (e.g., ablation at sites of fractionated electrograms) is not permitted unless the target is shown to be a critical isthmus or focal source in an organized atrial tachycardia (e.g., atypical atrial flutter). Delivery of ablation lesions that are not part of routine clinical practice is not recommended. Bidirectional conduction block across each ablation line will be confirmed with mapping and/or pacing as appropriate.<br><br>**Follow-Up**<br>A Pre-Discharge Visit will be conducted prior to hospital discharge after ablation. Telephone follow-up will be conducted at Day 10 (7-13 days) to assess medications and adverse events. Follow-up visits are at 1 month (Day 30, 14-42 days), 3 months (Day 90, 76-104 days), 6 months (Day 180, 150-210 days), and 12 months (Day 360, 330-390 days). Additional telephone follow-up will be conducted at 75 days (Day 75,

	60-90 days) unless the Day 90 office visit occurs within 90 days of the ablation procedure.<br><br>A transtelephonic monitor (TTM) will be provided to each subject prior to the end of the 90-day blanking period. Subjects will be instructed to transmit recordings at least monthly and when AF-related symptoms occur.<br><br>The Day 180 and Day 360 visits will include a 24-hour Holter monitor and QOL assessment.
**Neurological Assessment Sub-Study**	The objective of the Neurological Assessment Sub-Study is to evaluate the comparative incidence of acute cerebral ischemia (symptomatic and asymptomatic) as well as any associated neurological deficits. An initial cohort of at least 60 randomized subjects (at least 30 from each arm) will undergo neurological assessments before and after the ablation procedure and cerebral MRI after the ablation procedure.
**Data Safety and Monitoring Board**	The study will utilize a Data and Safety Monitoring Board (DSMB) including a statistician and three (3) individuals with relevant experience who are not participating investigators in this study. The DSMB will meet at regular intervals to review data from this clinical investigation and advise the Sponsor regarding the safety and well-being of subjects, any changes to the risk-benefit analysis, and the scientific validity of the study results.
**Clinical Events Committee**	The study will utilize an independent Clinical Events Committee (CEC) including three (3) physicians who are not participating investigators in this study. The CEC will meet at frequent intervals to adjudicate endpoint-related adverse events, classify adverse events according to severity and association with the device or procedure, and adjudicate death classifications (including primary cause and cardiac classification). The CEC may request additional information and assessments from the investigator to support adjudication.

Revision History

Revision	Change Order	Description
G	CO-002554	Record of approval and change history is in the EDCS

2 ABBREVIATIONS

**AAD**	Antiarrhythmic Drug
**ADE**	Adverse Device Effect
**AE**	Adverse Event
**AF**	Atrial Fibrillation
**CA**	Competent Authority
**CHF**	Congestive Heart Failure
**CPK**	Creatinine Phosphokinase
**CRF**	Case Report Form
**CS**	Clinically Significant
**CT**	Computed Tomography
**CVA**	Cerebrovascular Accident
**EC**	Ethics Committee
**ECG**	Electrocardiogram
**EF**	Ejection Fraction
**GCP**	Good Clinical Practices
**HM**	Holter Monitor
**ICD**	Implantable Cardioverter-Defibrillator
**ICE**	Intracardiac Echocardiography
**IRB**	Institutional Review Board
**LV**	Left Ventricular
**MI**	Myocardial Infarction
**MoCA**	Montreal Cognitive Assessment
**NCS**	Not Clinically Significant
**NIM**	Non-Inferiority Margin
**NOAC**	Non-Vitamin K Oral Anticoagulant
**PerAF**	Persistent Atrial Fibrillation
**PF**	Pulsed Field
**PHI**	Protected Health Information
**PRBCs**	Packed Red Blood Cells
**PV**	Pulmonary Vein
**PVI**	Pulmonary Vein Isolation
**QOL**	Quality of Life
**RF**	Radiofrequency
**SADE**	Serious Adverse Device Effect
**SAE**	Serious Adverse Event
**SCE**	Silent Cerebral Event
**SCL**	Silent Cerebral Lesion
**SOP**	Standard Operating Procedure
**TEE**	Transesophageal Echocardiogram
**TIA**	Transient Ischemic Attack
**TTE**	Transthoracic Echocardiogram
**TTM**	Transtelephonic Monitoring
**UADE**	Unanticipated Adverse Device Effect

3 Introduction

3.1 Background

3.1.1 Atrial Fibrillation

Atrial fibrillation (AF) is a supraventricular tachycardia that manifests as a rapid, irregular atrial rhythm with no clearly defined P-wave on the electrocardiogram. AF that terminates within seven days is defined as paroxysmal, while AF lasting longer than seven days is defined as persistent. Long-standing persistent AF is defined as continuous AF persisting for longer than 12 months [1].

AF carries significant risks. AF leads to dramatically increased risk of stroke and mortality, particularly among patients with persistent forms of AF. Furthermore, AF is often symptomatic, leading to fatigue and reduced quality of life (QOL); however, it carries similar risks with or without symptoms [1].

3.1.2 Demographic Profile of Atrial Fibrillation Patients

AF affects an estimated 37 million people worldwide [2]. The lifetime risk of developing AF after age 40 was 26% and 23% for men and women, respectively, in the Framingham Heart Study [3]. AF incidence rises rapidly with advancing age, and approximately one-fourth of adults diagnosed with AF are at least 80 years old [3, 4]. AF incidence is higher among whites than among African Americans [5].

Awareness of atrial fibrillation is poor [6], and disparities in awareness and treatment of AF have been identified. Factors identified as being associated with lack of awareness of AF have included race, lower education, rural location, lower number of general practitioner visits, and lower cognition [7, 8].

In the PRECEPT and STOP Persistent AF studies for catheter ablation to treat persistent AF (PerAF), the mean age was approximately 65 years, with a standard deviation of 9 years. Approximately 70% of treated subjects in each study were male. Less than 10% of subjects treated in each study were non-white, and less than 2% were Black or African American [9, 10].

3.1.3 Ablation of Atrial Fibrillation

Thermal ablation using radiofrequency (RF) energy has become a widely accepted treatment for many tachyarrhythmias and is considered first-line therapy in some cases [11, 1]. The success rate of RF catheter ablation for treating atrial fibrillation has been found to be superior to that of antiarrhythmic drugs. For example, the RAAFT-2 trial found that catheter ablation led to a 55% recurrence rate for atrial fibrillation or atrial tachycardia at 2 years follow-up compared to 72% recurrence using antiarrhythmic drugs [1]. Similarly, recurrence of AF, atrial flutter, or atrial tachycardia in the CABANA trial favored catheter ablation over drug therapy with a hazard ratio of 0.53, and analysis based on treatment received suggests a benefit from ablation in terms of death and hospitalization [12].

Catheter ablation is considered a reasonable treatment (Class IIa) for the treatment of symptomatic persistent AF refractory or intolerant to antiarrhythmic drugs. Two ablation catheters have been approved for treatment of drug-refractory persistent AF: the THERMOCOOL SMARTTOUCH® SF Catheter, which is a focal RF ablation catheter; and the Arctic Front Advance™ Cardiac Cryoablation Catheter. Electrical isolation of all pulmonary veins (PVs) is recommended (Class I) as part of all AF ablation procedures. However, PV reconnection is common, and repeat ablation is often necessary [1, 13].

Catheter ablation of AF is often accomplished by delivering focal RF energy in a point-by-point fashion to create circles (e.g. to isolate PVs) and/or lines (e.g. to block an arrhythmogenic channel). RF energy delivery through an electrode at the tip of an ablation catheter causes resistive heating in tissue, which, along with conductive heating, leads to thermal ablation of cardiac tissue. Features such as saline irrigation and temperature feedback can help to avoid the formation of thrombus and char due to blood heating during RF delivery [1].

Pulsed field (PF) ablation, also known as irreversible electroporation, relies on the application of non-thermal electrical pulses to form pores in the cell membranes of target tissue resulting in tissue apoptosis or necrosis. Recently published preclinical and clinical research suggests that PF ablation may be as effective as RF ablation while reducing risks associated with RF ablation such as damage to collateral structures [14, 15, 16, 17].

In the recent PRECEPT pivotal IDE study of the THERMOCOOL SMARTTOUCH® SF catheter for treatment of persistent AF (NCT02817776), only PV isolation was required; linear ablation lines including left atrial roof line, mitral isthmus line, left atrial floor line, and cavotricuspid isthmus line were only required to treat documented macro-reentrant atrial tachycardias. Results of the PRECEPT study showed that a left atrial roof line was delivered in just under half (48.6%) of the per-protocol group, and a cavotricuspid isthmus line was delivered in just over one-third (35.2%) of the per protocol group. A left inferior PV mitral line and other linear lesions were each delivered in less than 10% of patients in the per-protocol group (7.8% and 8.9%, respectively) [9].

3.1.4 Safety of AF Ablation

A review of data from the Nationwide Inpatient Sample between 2000 and 2010 found an overall incidence of complications of 6.3%, with a trend of increasing complications over that period [18]. A 2010 questionnaire-based survey of centers performing catheter ablation for atrial fibrillation reported a major complication rate of 4.5% [19]. A 2017 analysis of prospective data from the ESC-EHRA Atrial Fibrillation Ablation Long-Term Registry reported procedure-related complications in 7.8% [20]. In the recent PRECEPT pivotal IDE study of the THERMOCOOL SMARTTOUCH® SF catheter for treatment of persistent AF (NCT02817776), which used a similar primary safety endpoint to this protocol, the rate of the primary safety endpoint was 4.7% [21]. The PRECEPT study used an objective performance criterion (OPC) of 16%, based on an expected safety event rate of 8% and an 8% region of indifference, for analyzing the primary safety endpoint [22].

Pivotal IDE studies of focal catheter ablation for paroxysmal AF have also used similar primary safety endpoints. The DIAMOND-AF pivotal IDE study of the DiamondTemp ablation system for treatment of paroxysmal AF (NCT03334630) reported a primary safety event in 3.3% of subjects treated with the investigational device and in 6.6% of subjects treated with the control device (TactiCath™ Quartz) [23]. The SMART-SF study (NCT02359890), which evaluated the THERMOCOOL SMARTTOUCH® SF catheter for the treatment of paroxysmal AF, reported a primary adverse event in 2.6% of subjects (95% CI 0.7-6.5%). However, the SMART-SF study only evaluated early safety and acute effectiveness, so the chronic effectiveness of the treatments was not reported [24]. An OPC of 14% was used for analysis of the primary safety endpoint in SMART-SF [25]. The SMART-AF (NCT01385202) study was a pivotal IDE evaluating the THERMOCOOL SMARTTOUCH® catheter for treatment of paroxysmal AF, with primary safety and effectiveness endpoints similar to this protocol. The incidence of primary adverse events in SMART-AF was 9.9% (95% CI 5.8-15.6%) [26]. The OPC used for SMART-AF was not reported [27] but was presumably higher than the upper confidence bound of 15.6% based on the study's success.

3.1.5 Clinical Outcomes of AF Ablation

In a 2018 systematic literature review, single-procedure clinical success in treating persistent AF was estimated at 47% (95% CI 40-54%) across 18 cohorts between 2010 and 2015. Single-procedure clinical success in treating any AF with pulmonary vein isolation (PVI) plus linear ablation was estimated at 44% (95% CI 36-53%) across 10 cohorts over the same time period. Overall, this review found declining clinical success for AF ablation between 2001 and 2015 [28, 28]. The 2017 HRS consensus on AF ablation recommended a minimum acceptable success rate for treatment of persistent AF of 40% [1]. Subsequent single-arm pivotal IDE studies, including the PRECEPT study, used this minimum rate as an objective performance criterion for primary effectiveness. In the recent PRECEPT pivotal IDE study of the THERMOCOOL SMARTTOUCH® SF catheter for treatment of persistent AF (NCT02817776), primary effectiveness success was 59.3% through the end of the nine-month effectiveness evaluation period [21]. However, in contrast to this protocol, PRECEPT used a 6-month blanking and therapy consolidation period, and repeat ablation was allowed during that period (which occurred in 5.7%) [29].

3.2 Rationale for Clinical Investigation

Catheter ablation is the most widely used curative treatment for cardiac arrhythmia. However, the reliable application of durable ablation lesions remains an unmet need [1, 13, 30]. Despite recent advances in catheter ablation technology, conduction gaps remain in a substantial minority of ablation lines in the left atrium [31]. Gaps between lesions may lead to electrical reconnection in the heart and the recurrence of the treated arrhythmia, requiring repeated treatment, or may create new arrhythmia circuits that may be more symptomatic [11].

Contiguity of linear lesions depends on factors such as lesion size and placement [1]. Delivery of focal ablation lesions with a wider surface area may reduce the likelihood of gaps by increasing the probability of overlap between adjacent ablations in point-by-point ablation. An ablation electrode with stable tissue engagement may improve the predictability and reliability of lesion

placement. Temperature feedback from the tissue surface may provide improved feedback for RF energy titration and for delivery of contiguous sets of lesions. For these reasons, ablation through a large, focal ablation electrode may improve lesion contiguity. Furthermore, given research suggesting similar or better performance and reduced risks associated with damage to collateral structures, PF may prove to be an appropriate adjunct to RF for cardiac ablation, particularly in regions of the heart in which the risk of collateral damage with RF ablation is high.

4 Investigational Device Information

4.1 Intended Use

The **Sphere-9 Mapping and Ablation Catheter** and related accessories are intended to be used for:

- Cardiac electrophysiology mapping
- Delivering diagnostic pacing stimuli
- Creating endocardial lesions during cardiac ablation procedures for the treatment of persistent atrial fibrillation, when used with the Affera Mapping System and the Affera Ablation System to deliver radiofrequency (RF) or pulsed field (PF) energy

The **Affera Ablation System** is intended to be used with the Sphere-9 Mapping and Ablation Catheter in cardiac ablation procedures.

The **Affera Mapping System and accessories** are intended to be used for catheter-based atrial and ventricular mapping. The mapping system allows real-time visualization of intracardiac catheters as well as display of cardiac maps in multiple formats. The acquired patient signals, including intracardiac electrograms, may also be recorded and displayed on the system's display screen.

4.2 Device Description

The Sphere-9 Catheter and the Affera Mapping and Ablation System are designed to diagnose and treat cardiac arrhythmia using the same procedure workflow and the same or similar materials as currently marketed devices. The Sphere-9 Catheter and the Affera Ablation System are designed to deliver both radiofrequency (RF) ablation and pulsed field (PF) ablation [32, 33, 34].

The Sphere-9 Mapping and Ablation Catheter is a sterile, single-use 8F deflectable catheter that can be used for cardiac mapping and ablation. The Sphere-9 Mapping and Ablation Catheter (AFR-00001, shown in Figure 1) includes a handle to control bidirectional deflection of a 7Fr steerable shaft. The ablation electrode on the tip of the catheter is compressed to pass through a standard 8Fr introducer sheath or an 8.5Fr steerable guiding sheath and expands to 9mm in diameter when advanced beyond the sheath. The electrodes in the catheter tip are radiopaque for visualization via standard x-ray fluoroscopy.

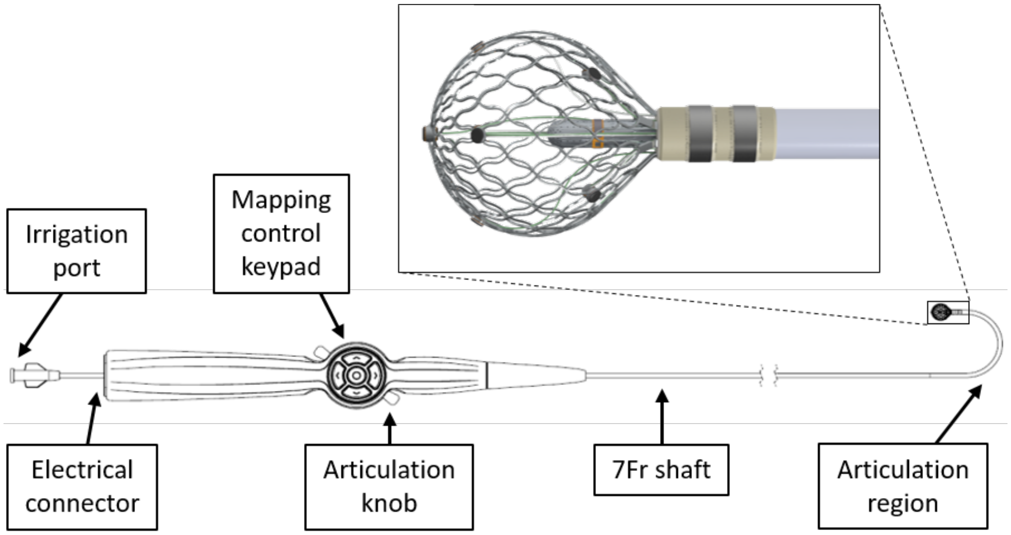

Figure 1. Sphere-9 Mapping and Ablation Catheter.

The Sphere-9 Mapping and Ablation Catheter contains an irrigation nozzle at the center of the expandable electrode. The nozzle is flushed with heparinized saline at a low flow rate (4mL/min) during mapping and is irrigated at a higher flow rate (15 – 30mL/min) during ablation. The irrigation nozzle is fed by a lumen that connects via a standard Luer connector to a custom tubing set and irrigation pump. The catheter contains passive electromagnetic sensors to allow display of the tip location and orientation by the Affera Mapping System.

The Affera Mapping and Ablation System comprises several individual components designed to operate together and listed in Table 1 and Table 2. The Affera Ablation System and the HexaMAP Catheter Interface Unit are shown in Figure 2.

Table 1. Affera Mapping System components.

Model Number	Component
AFR-00003	HexaMAP™ Catheter Interface Unit
	Prism-1 Software and Workstation
	Field Generator
	System Cart (optional)
AFR-00007	Location Reference Patch Kit

Table 2. Affera Ablation System components.

Model Number	System Component
AFR-00004	HexaGEN™ RF Generator
AFR-00008	HexaPULSE™ PF Generator Module
AFR-00005	HexaFLOW™ Irrigation Pump
AFR-00002	HexaFLOW™ Tubing Set
AFR-00006	Catheter Extension Cable

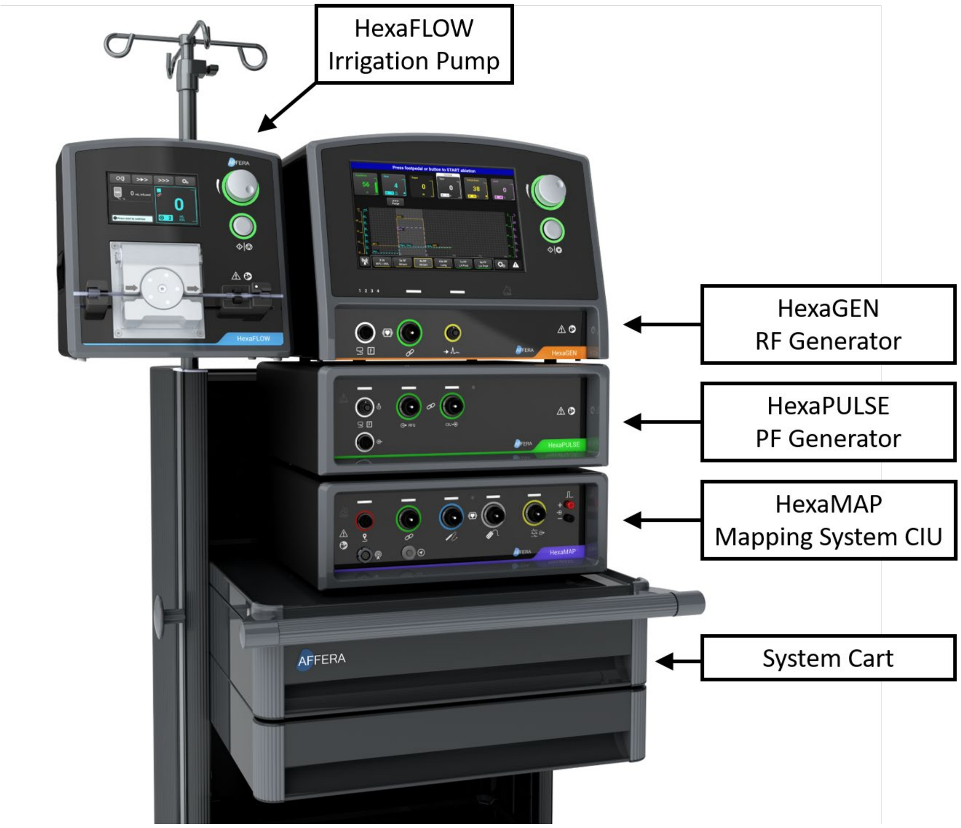

Figure 2. Affera Ablation System and HexaMAP Catheter Interface Unit.

The Affera Mapping System is a computerized storage and display system with embedded software which is designed to present the user with information regarding the state and location of one or more cardiac catheters.

The system can determine and present the three-dimensional location of a compatible catheter within the heart using one or more compatible electromagnetic sensors in the catheter. The location information can be used to generate a 3D geometric shell or electroanatomical map. A virtual representation of a compatible catheter can be displayed within the geometric shell in real

time in order to aid navigation of the catheter. The system can also tag location points of interest such as locations of energy delivery for reference relative to a geometric shell.

The Affera Mapping System has the capability to generate and route stimuli for pacing, allowing delivery of stimuli from the Sphere-9 Catheter as well as other catheters connected to the system. The Affera Mapping System also displays ablation settings and telemetry from the Affera Ablation System as well as ablation locations (tags) relative to the geometric shell.

The Affera Ablation System is capable of delivering either RF energy or PF energy to ablate cardiac tissue through the Sphere-9 Mapping and Ablation Catheter. The Affera Ablation System delivers ablative energy to the catheter, monitors ablation telemetry including catheter temperature and patient impedance, and controls irrigation to the catheter tip.

The Affera Ablation System is composed of:

- HexaGEN RF Generator (AFR-00004): Acts as the user interface and control unit of the Ablation System and delivers RF energy to the ablation catheter.
- HexaPULSE PF Generator module (AFR-00008): Delivers PF energy to the ablation catheter.
- HexaFLOW Irrigation Pump (AFR-00005): Provides controlled irrigation to cool the ablation electrode.

Electrical connection is made between the Affera Ablation System and the Sphere-9 Mapping and Ablation Catheter via a disposable Catheter Extension Cable (AFR-00006). The disposable HexaFLOW Tubing Set (AFR-00002) connects to the Sphere-9 Mapping and Ablation Catheter to provide saline irrigation.

The HexaGEN RF Generator delivers radiofrequency energy in the same manner as conventional RF generators used for cardiac ablation. However, the HexaGEN RF Generator is specifically designed for use with the Sphere-9 Mapping and Ablation Catheter, providing sufficient energy to deliver a therapeutic level of current density from the large surface area of the expandable ablation electrode. The HexaPULSE PF Generator module delivers electrical pulses to the Sphere-9 Mapping and Ablation Catheter ablation electrode.

To complete the electrical circuit for RF or PF energy delivery, the Ablation System requires the use of up to four disposable return electrodes. The system is compatible with return electrodes that are commercially available on the market and used for this purpose.

The HexaFLOW Irrigation Pump and custom HexaFLOW Tubing Set deliver irrigation solution to the Sphere-9 Catheter. The HexaFLOW Tubing Set is provided sterile for single-patient use.

5 Control Device Information

5.1 Indications for Use

The THERMOCOOL SMARTTOUCH® SF Catheter is indicated for the treatment of drug refractory recurrent symptomatic paroxysmal atrial fibrillation (AF) and for drug refractory recurrent symptomatic persistent AF (continuous AF > 7 days but < 1 year), refractory or intolerant to at least 1 Class I or III AAD, when used with the CARTO® 3 System.

The intended use of the CARTO® 3 System is catheter-based cardiac electrophysiological (EP) procedures. The CARTO® 3 System provides information about the electrical activity of the heart and about catheter location during the procedure. The system can be used on patients who are eligible for a conventional electrophysiological procedure. The system has no special contraindications.

The use of the SMARTABLATE™ RF Generator and all accessories is indicated in combination with compatible therapeutic catheters for use in conventional intracardiac RF ablation procedures.

6 Study Objectives

6.1 Primary Study Objectives

The objectives of this study are to evaluate the safety and effectiveness of the Sphere-9 Mapping and Ablation Catheter with the Affera Mapping and Ablation System for the treatment of symptomatic persistent atrial fibrillation (PerAF) refractory or intolerant to drugs using radiofrequency (RF) and pulsed field (PF) ablation. This study aims to demonstrate safety and effectiveness non-inferiority compared to the control device.

6.2 Neurological Assessment Sub-Study Objective

The objective of the Neurological Assessment Sub-Study is to evaluate the comparative incidence of acute cerebral ischemia (symptomatic and asymptomatic) as well as any associated neurological deficits.

7 Study Endpoints

7.1 Primary Safety Endpoint

The primary safety endpoint is the incidence of the following device- or procedure-related serious adverse events (SAEs) following the index ablation procedure:

Within 7 days:

- Death
- Myocardial infarction
- Phrenic nerve paralysis
- Transient ischemic attack (TIA)
- Stroke/cerebrovascular accident (CVA)
- Thromboembolism
- Major vascular access complications / bleeding
- Heart block
- Gastroparesis
- Severe pericarditis
- Hospitalization (initial and prolonged) due to cardiovascular or pulmonary AE‡

Within 30 days:

- Cardiac tamponade / perforation

Within 90 days:

- Atrio-esophageal fistula

Within 180 days:

- Pulmonary vein stenosis

‡ Excludes hospitalization due to AF/AFL/AT recurrence

Definitions related to Primary Aes are provided in Table 13. Adverse events occurring during or after a repeat ablation procedure will not be considered Primary Aes (see Section 9.10).

As discussed in Section 3.1.4, an objective performance criterion (OPC) of 16% was used for the primary safety endpoint in the PRECEPT study of the THERMOCOOL SMARTTOUCH® SF catheter for treatment of persistent AF (NCT02817776) based on an expected rate of 8% with an 8% region of indifference [22]. The reported rate of the primary safety endpoint in that study was 4.7% [21]. Other recent studies of RF ablation for the treatment of AF (paroxysmal or persistent) have reported primary adverse event rates as high as 9.9% [26]. For this study, a non-inferiority margin (NIM) of 8% between the investigational group and the control group will be used for the primary analysis of the Primary Safety Endpoint.

7.2 Primary Effectiveness Endpoint

The primary effectiveness endpoint is freedom from documented recurrence of AF, atrial tachycardia (AT), or atrial flutter (AFL) based on electrocardiographic data through 12-month follow-up and excluding a 90-day blanking period. The following are considered primary effectiveness endpoint failures:

- Inability to isolate all targeted pulmonary veins during the index procedure.

- Ablation using devices other than the assigned study device for any left atrial ablation during the index procedure. (The assigned study device is the Sphere-9 Catheter for the investigational arm and the THERMOCOOL SMARTTOUCH SF for the control arm.)
- Any repeat ablation, surgical ablation, or arrhythmia surgery for treatment of recurrent AF/AT/AFL after the index procedure.
- Documented AF/AT/AFL recurrence after the 90-day blanking period.
- Class I or III AAD dose increase from the historic maximum ineffective dose (prior to the index procedure) or initiation of a new Class I or III AAD for treatment of AF/AT/AFL after the 90-day blanking period.
- DC cardioversion for AF/AT/AFL after the 90-day blanking period.

A documented recurrence of AF/AT/AFL based on electrocardiographic data is (a) an episode ≥ 30 seconds in duration documented by ECG, TTM, or Holter monitor or (b) an episode covering an entire 12-lead ECG recording lasting at least 10 seconds.

The blanking period is the first 90 days after the index ablation procedure. The effectiveness evaluation period will be from Day 91 through Day 360.

Failure to complete the ablation procedure due to failure of non-study equipment (e.g., fluoroscopy, intracardiac echocardiography, or electrophysiology recording system) during the index procedure will not be considered a primary effectiveness endpoint failure. If the procedure cannot be completed due to failure of non-study equipment, the subject will be discontinued as described in Section 8.8.

Use of multiple devices of the assigned type during the ablation procedure (e.g., due to device deficiency) is not a primary effectiveness endpoint failure. Failure to isolate a pulmonary vein that was not targeted for isolation (e.g., due to unusual anatomy) is not a primary effectiveness endpoint failure.

The historical maximum ineffective dose for an AAD is the highest dose administered for AF that was ineffective in controlling AF or produced intolerable side effects prior to the ablation procedure. For amiodarone only, the maintenance dose is considered as the historic maximum ineffective dose. A new AAD is an AAD that the subject has never taken for AF prior to the ablation procedure.

During the blanking period, use of a new or previously failed AAD is not a primary effectiveness endpoint failure, regardless of dose.

Use of any new Class I or III AAD (as defined above) for treatment of AF/AT/AFL recurrence during the effectiveness evaluation period will be considered a primary effectiveness endpoint failure. Use of any previously failed Class I or III AAD at a dose greater than the historic maximum ineffective dose (as defined above) for treatment of AF/AT/AFL recurrence during the effectiveness evaluation period will be considered a primary effectiveness endpoint failure.

AAD dose changes made at the Day 90 visit, within the follow-up window, will be considered to have occurred within the 90-day blanking period.

As discussed in Section 3.1.5, an objective performance criterion (OPC) of 40% was used for the primary effectiveness endpoint in the PRECEPT study of the THERMOCOOL SMARTTOUCH® SF catheter for treatment of persistent AF (NCT02817776) [22]. An OPC of 40% was proposed as the minimum chronic acceptable success rate in the 2017 HRS consensus but is somewhat conservative compared to the range of success rates reported in a more recent meta-analysis [1, 28]. The reported rate of the primary effectiveness endpoint in the PRECEPT study was 59.3%, nearly 20% greater than an OPC of 40% [21]. For this study, a non-inferiority margin (NIM) of 15% between the investigational group and the control group will be used for the primary analysis of the Primary Effectiveness Endpoint.

7.3 Secondary Endpoints

Secondary Safety Endpoints:

- Incidence of early onset (within 7 days of the index ablation procedure) serious adverse events (SAEs)
- Incidence of peri-procedural (>7 and ≤30 days) SAEs
- Incidence of late onset (>30 days) SAEs

Secondary Effectiveness Endpoints:

- Changes in Quality of Life (QOL) using the Atrial Fibrillation Effect on Quality of Life (AFEQT) Questionnaire and the SF-12v2 Health Survey
- Rate of acute procedural success, defined as confirmation of entrance block in all targeted pulmonary veins
- Rate of pulmonary vein isolation on a per-vein basis
- Rate of acute block across linear ablations
- Use of antiarrhythmic drugs (AADs) during the effectiveness evaluation period
- Freedom from documented recurrence of symptomatic AF/AFL/AT through 12-month follow-up and excluding the 90-day blanking period
- Freedom from documented recurrence of symptomatic AF/AFL/AT off antiarrhythmic drug therapy through 12-month follow-up and excluding the 90-day blanking period

A documented recurrence of symptomatic AF/AT/AFL is (a) an episode ≥ 30 seconds in duration documented by ECG, TTM, or Holter monitor or (b) an episode covering an entire 12-lead ECG recording lasting at least 10 seconds. AF/AT/AFL is symptomatic if (a) the subject experiences arrhythmia-related symptoms that cause the subject to seek medical attention concurrent with documented recurrence or (b) the subject reports AF-related symptoms as part of a TTM recording that includes a documented recurrence.

Secondary Performance Endpoints:

- Investigator scoring of electroanatomical mapping
- Total fluoroscopy time during the ablation procedure

- Procedure time, defined as the time from elapsed from first venous access to last sheath removal
- Treatment time, defined as the time from the start of the first ablation delivery to the end of the last ablation delivery
- Total energy application time during the ablation procedure
- Ablation lesion sets delivered (ablation strategy)
- Ablation parameters per application
- Ablation energy type (RF or PF) used for each ablation lesion set

7.4 Neurological Assessment Sub-Study

The endpoints for the Neurological Assessment Sub-Study will be as follows:

- Incidence of silent cerebral events (SCE) and silent cerebral lesions (SCL) observed post-ablation
- Number, location, and size of observed SCE and SCL post-ablation, and during follow-up
- Change from baseline NIH Stroke Scale post-ablation and during follow-up
- Change from baseline Montreal Cognitive Assessment (MoCA) post-ablation and during follow-up

No acceptance criteria will be specified for the Neurological Assessment Sub-Study endpoints.

8 Study Design

This is a prospective, multicenter, randomized clinical evaluation of the Sphere-9 Mapping and Ablation Catheter with the Affera Mapping and Ablation System. Subjects will be randomly assigned 1:1 to receive treatment with either the Sphere-9 Mapping and Ablation Catheter and the Affera Mapping and Ablation System (investigational device) or the THERMOCOOL SMARTTOUCH® SF Catheter (control device). Subjects will be blinded to treatment assignment.

8.1 Population

The study population is patients with symptomatic persistent atrial fibrillation (PerAF) refractory or intolerant to drugs and planned for catheter ablation.

8.2 Clinical Sites

Subjects will be enrolled at up to 35 sites in the United States (US), Canada, Europe, Australia, and/or Israel, with up to approximately 50% of subjects enrolled outside the US.

8.3 Study Duration

The total duration of the clinical investigation is anticipated to be between 24 and 42 months. The enrollment phase is expected to take between 12 and 30 months from enrollment of the first subject.

8.4 Inclusion Criteria

Subjects must meet all of the following criteria to be included in the study:

1. Symptomatic PerAF documented by
   - (1) a physician's note indicating symptoms consistent with AF sustained longer than 7 days but less than 12 months;
     AND either
   - (2a) a 24-hour Holter documenting continuous AF within the past year
     OR
   - (2b) two electrocardiograms (from any form of rhythm monitoring, including consumer devices) taken at least 7 days apart within the past year, each showing continuous AF.
2. Failure or intolerance of at least one Class I or III anti-arrhythmic drug (AAD).
3. Suitable candidate for catheter ablation.
4. Adults aged 18 – 80 years.
5. Willing and able to comply with all baseline and follow-up evaluations for the full length of the study.
6. Willing and able to provide informed consent.

8.5 Exclusion Criteria

Subjects will not be eligible for the study if any of the following criteria are met:

1. Continuous AF lasting for 12 months or longer.
2. AF secondary to electrolyte imbalance, thyroid disease, acute alcohol intoxication, or other reversible or non-cardiac cause.

3. Previous left atrial ablation or surgical procedure (including septal closure or left atrial appendage closure).
4. Valvular cardiac surgical/percutaneous procedure (e.g., ventriculotomy, atriotomy, and valve repair or replacement and presence of a prosthetic valve).
5. Any carotid stenting or endarterectomy.
6. Any cardiac procedure (surgical or percutaneous) or percutaneous coronary intervention within the 90 days prior to the initial procedure.
7. Coronary artery bypass graft (CABG) procedure within the 6 months prior to the initial procedure.
8. Awaiting cardiac transplantation or other cardiac surgery within the 12 months following the initial ablation procedure.
9. Presence of a permanent pacemaker, biventricular pacemaker, or any type of implantable cardiac defibrillator (with or without biventricular pacing function).
10. Documented thromboembolic event (stroke or transient ischemic attack) within 6 months (180 days) prior to the initial ablation procedure.
11. Documented left atrial thrombus on imaging.
12. History of blood clotting or bleeding abnormalities.
13. Any condition contraindicating chronic anticoagulation.
14. Myocardial infarction (MI) within the 3 months (90 days) prior to the initial procedure.
15. Body mass index >40 kg/m^2.
16. Left atrial diameter >55 mm (anterioposterior).
17. Diagnosed atrial myxoma.
18. Left ventricular ejection fraction (EF) < 35%.
19. Uncontrolled heart failure or NYHA Class III or IV heart failure.
20. Rheumatic heart disease.
21. Hypertrophic cardiomyopathy.
22. Unstable angina.
23. Moderate to severe mitral valve stenosis.
24. Severe mitral regurgitation (regurgitant volume ≥ 60 mL/beat, regurgitant fraction ≥ 50%, and/or effective regurgitant orifice area ≥ 0.40cm^2).
25. Primary pulmonary hypertension.
26. Significant restrictive or obstructive pulmonary disease or chronic respiratory condition.
27. Renal failure requiring dialysis.
28. History of severe Gastroesophageal Reflux Disease (GERD) requiring surgical and/or mechanical intervention.
29. Acute illness, active systemic infection, or sepsis.
30. Contraindication to both computed tomography and magnetic resonance angiography
31. Significant congenital anomaly or medical problem that, in the opinion of the investigator, would preclude enrollment in this study or compliance with follow-up requirements or would impact the scientific soundness of the clinical study results.
32. Any woman known to be pregnant or breastfeeding, or any woman of childbearing potential who is not on a reliable form of birth regulation method or abstinence.
33. Current or anticipated participation in any other clinical study of a drug, device, or biologic during the duration of the study not pre-approved by the Sponsor.

34. Presence of intramural thrombus, tumor, or other abnormality that precludes vascular access, catheter introduction, or manipulation.
35. Known drug or alcohol dependency.
36. Life expectancy less than 12 months.
37. Vulnerable subject (such as a prisoner or handicapped or mentally disabled person).

8.6 Roll-In Phase

To ensure that investigators have adequate training with the investigational device, each site will be permitted to use the investigational device in up to two enrolled non-randomized Roll-In subjects. Subjects in the Roll-In Cohort will be analyzed separately from the Safety and Effectiveness Cohorts. Roll-In subjects in whom PVI cannot be completed (e.g., due to a device failure) will not count toward the limit of two Roll-In subjects per site.

8.7 Neurological Assessment Sub-Study

An initial cohort of at least 60 randomized subjects (at least 30 from each arm) will undergo neurological assessment before and after the ablation procedure and cerebral MRI after the ablation procedure. Roll-In Subjects will not participate in the Neurological Assessment Sub-Study. The first two subjects treated by an investigator with the Sphere-9 Catheter and the Affera Mapping and Ablation System will not participate in the Neurological Assessment Sub-Study.

Subjects will be provided with an explanation of the required procedures before signing the informed consent. Subjects will not be eligible for the Neurological Assessment Sub-Study if any of the following criteria are met:

1. Contraindication to MRI or to contrast agents used for MRI.
2. Unresolved pre-existing neurological deficit.

8.8 Subject Accountability

Subjects will be considered enrolled in the study when informed consent has been obtained. Subjects in the study will be classified as described below.

8.8.1 Screening Failure

A subject who has provided informed consent but does not meet eligibility criteria will be considered a screening failure. Minimum documentation of screening failure will include subject identification number, informed consent, eligibility criteria, and study exit. Screening failures do not count against the maximum study enrollment. There are no follow-up requirements for screening failures. Screening failures may not be re-enrolled in the study.

8.8.2 Excluded Subjects

Excluded subjects are those who are enrolled in the study and meet all eligibility criteria but do not undergo a mapping and/or ablation procedure involving the assigned study device (investigational or control). Minimum documentation of excluded subjects will include subject identification number, informed consent, eligibility criteria, and study exit. Excluded subjects do not count against the maximum study enrollment. Excluded subjects will not be included in evaluation of the study endpoints. There are no follow-up requirements for excluded subjects.

8.8.3 Discontinued Subjects

Discontinued subjects are those who are enrolled in the study, meet all eligibility criteria, and undergo a procedure in which the assigned study device is introduced into the heart, but no ablation energy is delivered. Subjects may be discontinued due to non-investigational equipment failure or based on intra-operative findings (e.g., if only non-study arrhythmias are present or if PVI is contraindicated due to a finding during the procedure). Discontinued subjects will count against the maximum study enrollment. Discontinued subjects will be followed through the Day 30 visit to evaluate safety and will be included in evaluation of study endpoints. Discontinued subjects may not be re-enrolled.

8.8.4 Treated Subjects

Treated subjects are those who are enrolled in the study, undergo the index ablation procedure, and are treated with a study device (i.e., ablation energy is delivered). Treated subjects will count toward the maximum study enrollment. Treated subjects will be followed through the Day 360 visit and will be included in evaluation of study endpoints.

8.8.5 Withdrawal and Lost to Follow-Up

While study withdrawal is discouraged, any subject may voluntarily withdraw from the study at any time, for any reason, without prejudice. If a subject withdraws from the study, the reason for withdrawal will be documented. The Sponsor must be notified of subject withdrawal within five (5) working days.

Subjects will be considered lost to follow-up if contact with the subject is lost after three (3) documented attempts to contact the subject including two (2) documented phone calls and one (1) written attempt by registered mail.

The investigator will make every effort to collect information about subjects who withdraw or are lost to follow-up. Withdrawal should be treated as an unscheduled office visit as described in Section 9.15.8. No additional data may be collected after a subject has been withdrawn. However, data collected up to the point of subject withdrawal may be used.

8.8.6 Completed Subjects

Completed subjects are enrolled subjects who have not been excluded, discontinued, withdrawn, or lost to follow-up and have completed the final study visit.

9 Study Procedures

The schedule of study evaluations and visits is summarized in Table 6. Study procedures are illustrated in the flowchart in Figure 3. Details are provided below.

9.1 Patient Screening

Patients ≥18 years with symptomatic PerAF refractory or intolerant to drugs who are planned for catheter ablation should be screened for study eligibility. Subjects who have provided informed consent but do not satisfy eligibility criteria will conclude their participation in the study and will not undergo treatment with the investigational device but may receive standard of care treatment outside of this study.

The Sponsor intends to enroll patients reflecting, as well as possible, the demographics of the study population. Similar to the recent PRECEPT and STOP Persistent AF trials for catheter ablation to treat PerAF, the Sponsor expects to enroll roughly 70% males and 30% females [9, 10].

9.2 Informed Consent

The investigator or a designated member of the research team will obtain written informed consent prior to any procedures that are specific to the investigation and fall outside of the standard of care, including any testing required to confirm eligibility for enrollment. The informed consent form must have approval from the site's IRB/EC prior to consenting patients for the study.

The investigator or designee will document the process of obtaining informed consent in the patient's source documents. The potential benefits and risks of participation will be explained to the patient. The patient will be provided a copy of the informed consent for review and will be given ample opportunity to review and ask questions. If the patient agrees to participate, both the patient and the investigator will sign the informed consent, and the patient will be recorded on an enrollment log. Failure to obtain informed consent renders the patient ineligible for participation.

Adverse events will be collected from the time informed consent is provided until the subject exits the study.

9.3 Roll-In

Each site will be permitted to use the investigational device in up to two non-randomized Roll-In subjects to allow investigators to become acquainted with the features of the investigational device. All Roll-In subjects will be treated with the investigational device. Subjects must be prospectively identified as Roll-In subjects during enrollment. Designation of a subject as a Roll-In subject is not permitted after randomization has been completed for the subject.

Roll-In procedures are not required prior to randomizing subjects at a site. Roll-In procedures are recommended to allow investigators not familiar with the investigational device to gain experience with the investigational device. Until the site has completed two Roll-In procedures,

additional Roll-In subjects may be designated at any time. Discontinued and excluded subjects do not count toward the maximum of two Roll-In subjects.

9.4 Randomization

Randomization must take place after informed consent and initial screening but may precede final determination of eligibility. For example, eligibility criteria that may be evaluated after randomization include those that are evaluated just prior to or during the index ablation procedure such left atrial thrombus (Exclusion Criterion 11), pregnancy (Exclusion Criterion 32), or abnormality precluding access/manipulation (Exclusion Criterion 34). However, eligibility criteria should be evaluated as fully as possible prior to randomization. Any eligibility failure after randomization should be carefully documented in order to minimize the potential for bias. If the subject is participating in the Neurological Assessment Sub-Study (see Section 9.9), enrollment in the Neurological Assessment Sub-Study must occur prior to randomization.

Subjects not designated as Roll-In will be randomized with a fixed 1:1 allocation to treatment with the investigational device or the control device. Subjects randomized to the investigational device will be treated with the Sphere-9 Catheter and the Affera Mapping and Ablation System. Subjects randomized to the control device will be treated with the THERMOCOOL SMARTTOUCH® SF radiofrequency ablation catheter. Subjects will be blinded to treatment assignment. Randomization will be blocked and stratified by site and by enrollment in the Neurological Assessment Sub-Study.

Randomized subjects will be blinded to treatment assignment during the study but may be informed of their treatment assignment after study exit upon request. The index ablation procedure must occur within 30 days after randomization.

9.5 Pre-Procedure/Baseline Assessments

Prior to the ablation procedure, the following assessments will be performed, unless previously performed as part of routine clinical evaluations within the specified window:

Within six (6) months prior to the ablation procedure:

- Transthoracic echocardiography (TTE) or transesophageal echocardiography (TEE) with measurements including atrial size and left ventricular ejection fraction (LVEF)

Within 30 days prior to the ablation procedure:

- Evaluation of inclusion/exclusion criteria
- Demographics including age, gender, race, and ethnicity
- Medical history including:
  - Arrhythmia history including type, duration, frequency, symptoms, and prior treatments (e.g., medication, cardioversion, and/or previous ablation)
  - Heart disease
  - Thromboembolic events
  - CHA_2DS_2-VASc score
  - NYHA class
- Baseline medications including cardiac medications and anticoagulation
- Physical exam, including cardiovascular/pulmonary examination

- 12-lead ECG
- Baseline Quality of Life (QOL) using the Atrial Fibrillation Effect on Quality of Life (AFEQT) Questionnaire and the SF-12v2 Health Survey

Within seven (7) days prior to the ablation procedure:

- Women of childbearing potential will have a pregnancy test

Within three (3) days prior to the ablation procedure:

- NIH Stroke Scale performed by trained staff

Either the day before or the day of the ablation procedure:

- Thrombus screening as described in 9.7

Any adverse events will be recorded. Any abnormalities will be categorized as clinically significant (CS) or not clinically significant (NCS) and recorded.

See Section 9.9 for additional assessments required for subjects in the Neurological Assessment Sub-Study.

9.6 Anticoagulation

9.6.1 Systemic Anticoagulation Prior to Ablation

Uninterrupted therapeutic anticoagulation is required for at least three weeks prior to an ablation procedure. For subjects on warfarin, INR should be measured weekly for at least three weeks prior to the ablation procedure. INR $\geq$ 2.0 is considered therapeutic. Oral anticoagulants must be continued through the ablation procedure, without any doses held [1, 35, 36].

9.6.2 Intraprocedural Anticoagulation

Heparin will be administered during an ablation procedure to achieve a target activated clotting time (ACT) of at least 300 seconds. An initial heparin bolus will be administered prior to transseptal puncture. Heparinized saline will be infused through all long sheaths with access to the left atrium. ACT will be checked within 15 minutes until the target ACT is achieved and minimally at 30 minute intervals thereafter [1]. The target ACT should be maintained while the study catheter is in the body. If the ACT drops below 300 seconds during the ablation procedure, the ablation procedure may continue while additional heparin is administered.

9.6.3 Early Postprocedural Anticoagulation

Following an ablation procedure, systemic anticoagulation therapy is required for at least two months [1]. Decisions regarding continuation of systemic anticoagulation more than 2 months post ablation should be based on the patient's stroke risk profile and not on the perceived success or failure of the ablation procedure.

9.7 Thrombus Screening

Left atrial thrombus screening will be performed the day before or the day of an ablation procedure using either transesophageal echocardiography (TEE) or intracardiac echocardiography (ICE). If thrombus is observed prior to or during the index ablation procedure,

the subject does not meet the study eligibility criteria and may not undergo the index ablation procedure.

9.8 ABLATION PROCEDURE

The goal of the index ablation procedure is isolation of all PVs as demonstrated by entrance block. Starting with transseptal puncture, the ablation procedure may only be performed by authorized investigators.

Photos, video, and/or audio recordings of the procedure may be taken during the ablation procedure for future training purposes.

The following sequence is recommended for the ablation procedure:

- Esophagus temperature probe placement
- Diagnostic catheter placement (e.g., in the coronary sinus)
- Thrombus screening (if ICE is used)
- Initial heparin bolus
- Confirmation of target ACT, with monitoring minimally at 30-minute intervals thereafter
- Transseptal puncture
- Aspiration and flushing of transseptal sheath(s)
- Introduction of catheter(s) into the left atrium
- High-density mapping of the left atrium
- Phrenic nerve pacing
- PV isolation
- Mapping and ablation of additional atrial tachycardias
- Confirmation of PV isolation after waiting period
- Confirmation of block across linear lesions
- Screening for phrenic nerve injury

Additional details are provided below.

A single or double transseptal puncture may be performed at the discretion of the investigator. A constant heparinized drip is recommended for all long sheaths with access to the left atrium.

If an esophagus temperature probe is used, it is recommended that the probe be placed prior to administration of a heparin bolus. Thrombus screening is required prior to introduction of the ablation catheter and prior to any cardioversion. The target ACT should be maintained while the ablation catheter is in the body. If the ACT drops below the target during the ablation procedure, the ablation procedure may continue while additional heparin is administered.

Cardioversion may be used to restore sinus rhythm at the discretion of the investigator.

At the end of the procedure, phrenic nerve function must be assessed by either (a) direct pacing of the phrenic nerve or (b) observation of diaphragm motion on fluoroscopy during spontaneous breathing. If new phrenic nerve injury is observed or suspected based on assessment at the end of

the ablation procedure, a sniff test or inhalation-exhalation fluoroscopy will be performed prior to discharge.

9.8.1 Pulmonary Vein Isolation

See Sections 9.8.4 and 9.8.5 for specific requirements and recommendations regarding ablation with the investigational device and the control device.

High-density mapping of the left atrium with the assigned mapping system (i.e., the Affera Mapping System or the CARTO® 3 System) is required prior to pulmonary vein isolation. Prior to any RF delivery near the right superior PV (RSPV) ostium, pacing maneuvers must be used to evaluate phrenic nerve location.

Wide antral circumferential ablation (WACA, i.e., adjacent ablation lesions encircling ipsilateral veins 1 – 2 cm outside of the vein ostium) must be used to encircle and isolate all targeted PVs.

Entrance block will be confirmed in each targeted PV (a) after a 20-minute waiting period from the last ablation delivery for that PV or, as part of routine practice at experienced centers with prior approval from the Sponsor, (b) during provocative testing with adenosine or isoproterenol. It is recommended that entrance block be confirmed using a circular or multi-spline catheter or, for subjects treated with the investigational device, the Sphere-9 Catheter (see section 9.8.4). According to the 2017 HRS consensus on AF ablation, "Entrance block of the left PVs should be assessed during distal coronary sinus or left atrial appendage pacing in order to distinguish far-field atrial potentials from PV potentials." [1]

Failure to isolate all targeted PVs will be considered a primary effectiveness endpoint failure. Ablation using devices other than the assigned study device to isolate a targeted PV will also be considered a primary effectiveness endpoint failure. Failure to isolate a PV that was not targeted for isolation (e.g., due to unusual anatomy) is not a primary effectiveness endpoint failure.

9.8.2 Additional Ablation

See Section 9.8.4 and 9.8.5 for specific requirements and recommendations regarding ablation with the investigational device and the control device.

Any ablation beyond PVI for treating AF, if required, should be performed with the assigned study catheter if possible. Any AF ablation not completed with the assigned study catheter must be documented. Ablation using devices other than the assigned study device for any left atrial ablation during the index procedure will be considered a Primary Effectiveness Endpoint failure.

The following linear ablations are only required in order to treat documented macroreentrant tachycardias:

- Linear lesions between the tricuspid annulus and the inferior vena cava (cavotricuspid isthmus line)
- Linear lesions between the left and right PVs (roof line and/or posterior line), optionally including homogenization of the posterior wall of the left atrium

- Linear lesions between the mitral annulus and the left PVs (mitral isthmus line), with ablation permitted in the apposing coronary sinus.

Cavotricuspid isthmus linear ablation is required in cases with documented typical right atrial flutter either prior to or during the procedure. Empirical atrial linear ablation is discouraged.

Additional ablation beyond these lesion sets (e.g., ablation at sites of fractionated electrograms) is not permitted unless the target is shown to be a critical isthmus or focal source in an organized atrial tachycardia (e.g., atypical atrial flutter).

Delivery of ablation lesions that are not part of routine clinical practice is not recommended. Bidirectional conduction block across each ablation line must be confirmed with mapping and/or pacing as appropriate.

9.8.3 Data Collection

Data collected during the procedure will include the following:

- Adverse events
- Cardiac medications
- Device deficiencies
- Rhythm(s) prior to and during the ablation procedure
- Rhythm at the end of the ablation procedure
- Devices used for mapping and/or verification
- Lesion sets applied
- Method(s) of confirming each lesion set
- Use of RF and/or PF for each lesion set/group
- Device(s) used for each lesion set/group
- Total number of RF and PF deliveries
- Total RF and PF delivery time
- Total fluid infused from mapping and ablation catheters
- Total fluoroscopy time
- Time(s) of catheter introduction and removal
- Ablation data (electronic or paper records) captured by the mapping system, ablation system, and/or EP recording system

After the ablation procedure, an investigator involved in performing the procedure will evaluate electroanatomical mapping performance. Scoring of electroanatomical mapping will be on a scale from 1 – 5, with 1 representing a strongly negative response, 5 representing a strongly positive response, and 3 being neutral or just acceptable.

9.8.4 Investigational Device Use

For subjects randomized to receive treatment with the investigational device, the following devices are required to be used in the ablation procedure for mapping and ablation:

- Sphere-9 Mapping and Ablation Catheter
- Affera Mapping System, including the HexaMAP Catheter Interface Unit (CIU)

- Affera Ablation System, including the HexaGEN RF Generator, HexaPULSE PF Generator Module, and HexaFLOW Irrigation Pump

The lot or serial number of each of these devices will be recorded. The investigational device must be used according to the instructions for use.

Recommended settings for RF ablation with the Sphere-9 Catheter are shown in Table 3, and recommended settings for PF ablation with the Sphere-9 Catheter are shown in Table 4.

Table 3. Recommended RF ablation settings for Sphere-9 Catheter.

Setting	Duration	Current Limit	Temp. Target	Irrigation Rate
RF-ANT	5s	80%	73°C	30mL/min
RF-MI	5s	90%	73°C	30mL/min

Table 4. Recommended PF ablation settings for Sphere-9 Catheter.

Setting	Duration	Current Limit	Irrigation Rate
PULSE3	4s	95%	15mL/min

RF and PF energy will be used as follows:

- Only PF energy will be delivered on or near the posterior wall of the left atrium.
- Only PF energy will be delivered around the antrum of the left inferior PV (LIPV).
- Only PF energy will be used when ablating near a location where pacing captures the phrenic nerve.
- PF energy may also be delivered in other areas where PF may have safety benefits (e.g., near coronary arteries, or in the coronary sinus).
- Unless otherwise specified, RF energy will be used for all other ablation.

The RF-ANT ablation setting is recommended for most RF ablation. The RF-MI ablation setting is recommended only for endocardial ablation along the mitral isthmus.

For subjects treated with the investigational device, it is recommended that the Sphere-9 Catheter be used for the required confirmation of entrance block in each targeted PV. The Sphere-9 Catheter can be used to assess PV entrance block by moving the Sphere-9 Catheter tip to multiple positions encircling the PV antrum and using the electrograms measured by the mini surface electrodes to detect PV potentials. Absence or dissociation of PV potentials within the PV antrum is indicative of entrance block.

9.8.5 Control Device Use

For subjects randomized to receive treatment with the control device, the following devices are required to be used in the ablation procedure for mapping and ablation:

- THERMOCOOL SMARTTOUCH® SF Catheter

- SMARTABLATE® System
- CARTO® 3 System

These devices should be prepared and deployed according to the instructions for use, user manuals, and physician training. Recommended settings for RF ablation with the THERMOCOOL SMARTTOUCH SF® Catheter are shown in Table 5.

Table 5. Recommended RF ablation settings for THERMOCOOL SMARTTOUCH SF® catheter.

**Power Range**	**Temperature Monitoring**	**Irrigation Flow Rate during RF Application**	**Application Time**
15 W to 30 W*	< 40°C**	8 mL/min	30 to 120 seconds

* Power levels exceeding 30 watts may be used when transmural lesion cannot be achieved at lower energy levels. For power settings > 30 watts, the recommended irrigation flow rate is 15 mL/min.

** The temperature displayed on the SMARTABLATE system does not represent tissue temperature or electrode tissue interface temperature.

For isthmus dependent flutter ablation, power applications exceeding 30 watts and up to 50 watts should only be used if conduction block cannot be achieved at lower power levels.

Based on contact force values recorded in the PRECEPT study [9], contact force recommendations are as follows:

- It is recommended that the working range of contact force in the real-time rolling graph be approximately 5 to 40 g, as this was the most frequently selected contact force working range reported in the PRECEPT study.
- It is recommended that the overall average contact force applied during the ablation procedure (session) target roughly 12 to 18 g, as this range represents roughly the first and third quartile of the average contact force during ablation procedures reported in the PRECEPT study.

For subjects treated with the control device, it is recommended that a circular or multi-spline catheter be used for the required confirmation of entrance block in each targeted PV.

9.9 Neurological Assessment Sub-Study

For subjects in the Neurological Assessment Sub-Study, cerebral magnetic resonance imaging (MRI) will be conducted after the ablation procedure. Diffusion-weighted imaging (DWI) and fluid-attenuated inversion recovery (FLAIR) sequences will be used to identify ischemia as described below. It is recommended that cerebral MRI be conducted within 24 – 72 hours after the index ablation procedure, targeting 24 – 48 hours.

To allow for adequate detection of ischemia and facilitate interpretation of study results, the standardized MRI protocol described below will be used [37, 38, 39, 40, 41]. Imaging will be performed with a 1.5T scanner. An anatomical localizer image will be collected to facilitate subject positioning if repeat imaging is performed. FLAIR and DWI sequences will be collected as follows:

- T2-weighted axial FLAIR sequence:
    - Slice thickness: 5 mm
    - Field of view: set to encompass the entire brain (typically 230mm)
    - Matrix: 256
    - Repetition time (TR) may vary but is typically 8500 – 9000
    - Echo time (TE) may vary but is typically 100 – 120ms
    - Inversion Time (TI): 2500ms
- Axial DWI sequence:
    - Slice thickness: 5 mm
    - Field of view: set to encompass the entire brain (typically 230mm)
    - Matrix: 128
    - Repetition time (TR) may vary but is typically 3200 – 3500ms
    - Echo time (TE) may vary but is typically between 80 and 100ms
    - Two distinct *b* values, 0 and 1000s/mm^2, in three diffusion directions
    - For each DWI sequence, an apparent diffusion coefficient (ADC) map will be obtained.

The slices and orientation of the DWI and FLAIR sequences should be matched.

Image analysis will be performed by a core lab. Post-procedure images will be analyzed for the following ischemic findings:

- Silent cerebral events (SCE), defined as acute ischemia identified based on focal hyperintensity on DWI with corresponding focal reduction of apparent diffusion coefficient (ADC) at the same location
- Silent cerebral lesions (SCL), defined as SCE with corresponding focal hyperintensity on FLAIR.

The number, location, and approximate dimensions of any SCE and SCL will be evaluated.

If SCE or SCL are identified in post-procedure imaging, cerebral MRI will be repeated at the Day 90 follow-up visit. Repeat imaging will be performed with the same sequence parameters to allow comparison. The subject should be repositioned carefully in the scanner with reference to anatomical localizer images from the previous assessment. Unresolved lesions in repeat MRI will be identified based on focal hyperintensity on the FLAIR image at sites of previously identified SCE/SCL.

NIH Stroke Scale is performed for all subjects in the study. (See sections 9.5 and 9.15.1.) NIH Stroke Scale will be performed by trained staff within 72 hours both before and after the ablation procedure. Montreal Cognitive Assessment (MoCA) will be performed by trained staff within 72 hours before the ablation procedure and again between 24 and 72 hours after the ablation procedure (i.e., no less than 24 hours and no more than 72 hours after the ablation procedure). NIH Stroke Scale and MoCA will be performed again at the Day 30 visit. If new neurologic symptoms/deficits identified after the ablation procedure are unresolved at the Day 30 visit, NIH Stroke Scale and MoCA will be performed again at the Day 90 follow-up visit. Any subject with symptoms of stroke or transient ischemic attack (TIA) will be evaluated by a neurologist.

Subjects in the Neurological Assessment Sub-Study will undergo ablation as randomized.

9.10 Repeat Ablation

Repeat ablation for treatment of recurrent AF/AT/AFL at any time after the index ablation procedure will be considered a primary effectiveness failure. Any repeat ablation must be more than 30 days after the index ablation procedure. It is strongly recommended that any repeat ablation be more than 90 days after the index procedure. Repeat ablation using the investigational device is not permitted.

If a repeat ablation procedure is performed:

- Diagnostic evaluation related to adverse events (e.g., chest CT or MRI for subjects with symptoms suggestive of PV stenosis) should be completed prior to a repeat ablation procedure.
- See Section 9.6 for anticoagulation requirements.
- Thrombus screening must be repeated according to Section 9.7.
- A pregnancy test must be repeated for women of childbearing potential.
- Assessments will include medical history as well as cardiac medications and anticoagulation
- The schedule of follow-up visits will not reset.

9.11 Antiarrhythmic Drugs (AADs)

See Section 7.2 for definitions of AAD use related to the Primary Effectiveness Endpoint.

During the 90-day blanking period, subjects may receive new AADs or previously failed Class I or III AADs without affecting their primary effectiveness classification. It is recommended that a Class I or III AAD be selected according to the ACC/AHA/ESC 2014 Guidelines for the Management of Patients with AF [42]. Initiation, re-initiation, or up-titration of amiodarone is not recommended post ablation. In addition, it is recommended to discontinue use of Class I or III AADs used for treating AF/AT/AFL by the end of the 90-day blanking period.

During the effectiveness evaluation period (Day 91 through Day 360):

- Continuation or initiation of any new Class I or III AAD for treating AF/AT/AFL recurrence will be considered a primary effectiveness endpoint failure.
- Use of any previously failed Class I or III AAD at a dose greater than the historic maximum ineffective dose for treating AF/AT/AFL recurrence will be considered a primary effectiveness endpoint failure.

Use of a previously failed AAD at or below the historical maximum ineffective dose during the effectiveness evaluation period is not a primary effectiveness endpoint failure.

9.12 Transtelephonic Monitoring (TTM)

A transtelephonic monitor (TTM, or other event monitoring device) will be provided to each subject prior to the end of the 90-day blanking period. Subjects will be instructed to transmit recordings at least monthly and when AF-related symptoms occur. Subjects will use the

monitoring device to report symptoms at the time of transmission. Missed TTM recordings will not be considered a protocol deviation.

TTM recordings will be analyzed by a core laboratory. Episodes of atrial tachyarrhythmia (AF/AFL/AT) recorded on TTM within the effectiveness evaluation period and lasting longer than 30 seconds will be reviewed by the investigator at the Day 180 and Day 360 visits. Symptomatic and asymptomatic recurrences will be identified by the core laboratory. The number of TTM recordings will be documented.

9.13 Holter Monitoring (HM)

Prior to the Day 180 and Day 360 visits, each subject will have a 24-hour Holter Monitor (HM). HM recordings will be analyzed by a core laboratory. Episodes of atrial tachyarrhythmia (AF/AFL/AT) recorded on HM within the effectiveness evaluation period and lasting longer than 30 seconds will be reviewed by the investigator at the Day 180 and Day 360 visits.

9.14 12-Lead ECG Recordings

12-lead ECG will be recorded as part of any in-person visits at Day 30, Day 90, Day 180, and/or Day 360, as well as any unscheduled visit (if possible). 12-lead ECG recordings during the effectiveness evaluation period will be analyzed by a core laboratory to identify episodes of atrial tachyarrhythmia (AF/AFL/AT) (a) lasting longer than 30 seconds or (b) covering an entire recording lasting at least 10 seconds.

9.15 Follow-Up Visits

A Pre-Discharge Visit will be conducted prior to hospital discharge after ablation. Telephone follow-up will be conducted at Day 10 (7-13 days) to assess medications and adverse events. Follow-up visits are at 1 month (Day 30, 14-42 days), 3 months (Day 90, 76-104 days), 6 months (Day 180, 150-210 days), and 12 months (Day 360, 330-390 days). The Day 30 visit may be either an in-person visit or a “hybrid” visit as described in section 9.15.3. Unless an office visit is needed to assess adverse events or as part of the Neurological Assessment Sub-Study, office visits at Day 90, Day 180, and Day 360 may be conducted as telehealth visits, with transmission of TTM recordings in lieu of 12-lead ECG. Additional telephone follow-up will be conducted at 75 days (Day 75, 60-90 days) unless the Day 90 office visit occurs within 90 days of the ablation procedure.

A post-approval study with subject follow-up for up to 5 years may be requested by the U.S. Food and Drug Administration (FDA) or other regulatory body as a condition of approval of the investigational device. As a result, follow-up visits including medical history, arrhythmia history, and adverse events may extend for up to 5 years.

The follow-up schedule will not reset if the subject undergoes a repeat ablation procedure.

9.15.1 Pre-Discharge Visit

Prior to hospital discharge after ablation, assessments will include the following:

- Physical exam, including cardiovascular/pulmonary examination

- 12-lead ECG
- TTE is required if the subject has symptoms suggestive of pericardial effusion and/or pericarditis.
- Cardiac medications and anticoagulation
- Occurrence of arrhythmias
- Adverse events
- NIH Stroke Scale performed by trained staff. If the NIH Stroke Scale demonstrates new abnormal findings as compared to baseline, the subject should have a formal neurology consult and formal neurological exam with appropriate imaging (e.g., cerebral MRI including DWI) to confirm any suspected diagnosis of stroke. See Table 13 for definitions of stroke and cerebrovascular accident.
- If new phrenic nerve injury was observed or suspected based on assessment at the end of the ablation procedure, a sniff test or inhalation-exhalation fluoroscopy is required prior to discharge. If the phrenic nerve injury is still unresolved, the assessment will be repeated at the Day 30 visit.
- See Section 9.9 for additional assessments for subjects in the Neurological Assessment Sub-Study.

9.15.2 DAY 10 TELEPHONE CALL (7-13 DAYS)

The Day 10 telephone call will include the following assessments:

- Medical history
- Cardiac medications and anticoagulation
- Adverse events

9.15.3 DAY 30 OFFICE/HYBRID VISIT (14-42 DAYS)

The Day 30 visit may be either an in-person office visit or, under specific circumstances as described below, a “hybrid” visit including an office visit with the referring cardiologist and a telephone call with the investigator.

- If the subject has an in-person office visit with the referring cardiologist prior to the Day 30 Visit, and if the investigator is able to review the chart from the referring cardiologist, then the Day 30 Visit may be performed by telephone rather than in person. The visit with the referring cardiologist (and the chart) must include a physical exam, minimally including a cardiovascular/pulmonary examination and 12-lead ECG.
- If the subject does not have an in-person visit with the referring cardiologist prior to the Day 30 Visit, or if the chart from the referring cardiologist does not allow the investigator to review the results of the physical exam, then the Day 30 Visit must be an in-person office visit at the study site.

The Day 30 visit will include at least the following assessments:

- Physical exam, including cardiovascular/pulmonary exam and 12-lead ECG (performed by the study site or by the referring cardiologist as described above)
- Medical history
- Cardiac medications and anticoagulation

- Adverse events
- PV anatomy imaging (chest CT or MRI) is required if the subject has symptoms suggestive of PV stenosis.
- If there was new phrenic nerve injury during the index ablation procedure that was unresolved at the Pre-Discharge Visit, a sniff test or inhalation-exhalation fluoroscopy is required at the Day 30 visit. If the phrenic nerve injury is still unresolved, the assessment will be repeated at the Day 360 visit.
- See Section 9.9 for additional assessments and requirements for subjects in the Neurological Assessment Sub-Study.

9.15.4 Day 75 Telephone Call (60-90 days)

If the Day 90 visit does not occur within 90 days of the procedure (on or before day 90), additional telephone follow-up will be conducted at 75 days to assess cardiac medications. See Section 9.11 regarding AAD use during and after the blanking period. Any adverse events will be documented.

9.15.5 Day 90 Office/Telehealth Visit (76-104 days)

The Day 90 office visit will include the following assessments:

- 12-lead ECG (if office visit) or TTM transmission (if telehealth visit)
- Medical history
- Arrhythmia history
- Cardiac medications and anticoagulation
- Adverse events
- PV anatomy imaging (chest CT or MRI) is required if the subject has symptoms suggestive of PV stenosis.
- See Section 9.9 for additional assessments and requirements for subjects in the Neurological Assessment Sub-Study.

See Section 9.11 regarding AAD use during and after the blanking period.

9.15.6 Day 180 Office/Telehealth Visit (150-210 days)

Subjects will be provided with a Holter Monitor prior to the Day 180 visit. Results of Holter Monitor and TTM will be reviewed at the Day 180 visit.

Unless an office visit is needed to assess adverse events, the Day 180 visit may optionally be conducted as a telehealth visit with transmission of TTM recordings during the visit. The Day 180 visit will include the following assessments:

- QOL assessment using the Atrial Fibrillation Effect on Quality of Life (AFEQT) Questionnaire and the SF-12v2 Health Survey. These assessments should be completed before the other assessments during this visit.
- 12-lead ECG (if office visit) or TTM transmission (if telehealth visit)
- Medical history
- Arrhythmia history (TTM, HM, etc.) and symptoms
- Cardiac medications and anticoagulation

- Adverse events
- PV anatomy imaging (Chest CT or MRI) is required if the subject has symptoms suggestive of PV stenosis.

9.15.7 DAY 360 OFFICE/TELEHEALTH VISIT (330-390 DAYS)

Subjects will be provided with a Holter Monitor prior to the Day 360 visit. Results of Holter Monitor and TTM will be reviewed at the Day 360 visit.

Unless an office visit is needed to assess adverse events, the Day 360 visit may optionally be conducted as a telehealth visit with transmission of TTM recordings during the visit. The Day 360 visit will include the following assessments:

- QOL assessment using the Atrial Fibrillation Effect on Quality of Life (AFEQT) Questionnaire and the SF-12v2 Health Survey. These assessments should be completed before the other assessments during this visit.
- 12-lead ECG (if office visit) or TTM transmission (if telehealth visit)
- Medical history
- Arrhythmia history (TTM, HM, etc.) and symptoms
- Cardiac medications and anticoagulation
- Adverse events
- PV anatomy imaging (chest CT or MRI) is required if the subject has symptoms suggestive of PV stenosis.
- If there was new phrenic nerve injury during the index ablation procedure that was unresolved at the Pre-Discharge Visit and the Day 30 Visit, a sniff test or inhalation-exhalation fluoroscopy is required at the Day 360 visit.

Prior to unblinding, the subject will be asked to guess which study arm he/she was assigned to.

9.15.8 UNSCHEDULED VISITS

An unscheduled visit is any unplanned cardiovascular-related visit with the study site that occurs outside of protocol-driven visits, including early study exit. Unscheduled visits will include the following assessments:

- Medical history
- Arrhythmia history
- Cardiac medications and anticoagulation
- Adverse events
- PV anatomy imaging (chest CT or MRI) is required if the subject has symptoms suggestive of PV stenosis.

The following assessments should be performed if possible:

- Physical exam
- 12-lead ECG

Table 6. Schedule of evaluations and visits.

	**Enrollment / Baseline**	**Ablation**	**Pre-Discharge**	**Day 10 Call**	**Day 30 Visit**	**Day 75 Call[1]**	**Day 90 Visit**	**Day 180 Visit**	**Day 360 Visit**	**Repeat Ablation**	**Unsched.**
Window (days)		*0*		*7-13*	*14-42*	*60-90*	*76-104*	*150-210*	*330-390*		
**Informed consent**	✓										
**Adverse events**	✓	✓	✓	✓	✓	✓	✓	✓	✓	✓	✓
**Eligibility criteria**	✓										
**Demographics**	✓										
**Medical history / hospitalization**	✓			✓	✓		✓	✓	✓	✓	✓
**Cardiac med.**	✓	✓	✓	✓	✓	✓	✓	✓	✓	✓	✓
**Physical exam**	✓		✓		✓						✓
**12-Lead ECG**	✓		✓		✓[2]		✓[3]	✓[3]	✓[3]		✓
**TTE / TEE**	✓[4]		✓[5]								
**Thrombus screening[6]**	✓[6]									✓[6]	
**Pregnancy test[7]**	✓[7]									✓[7]	
**Randomization**	✓										
**Ablation**		✓								✓	
**Phrenic nerve**		✓	✓[8]		✓[8]				✓[8]		
**NIH Stroke Scale**	✓		✓								
**PV anatomy[9]**					✓[9]		✓[9]	✓[9]	✓[9]		✓[9]
**TTM transmission**							✓	✓	✓		
**Holter Monitor**								✓	✓		
**TTM/Holter review**								✓	✓		
**Quality of Life**	✓							✓	✓		

[1] Required if the Day 90 visit is not performed within the blanking period (on or before day 90)
[2] May be performed by the referring cardiologist as part of a "hybrid" visit under circumstances described in Section 9.15.3
[3] If the visit is a telehealth visit, transmission of TTM recordings will be performed in lieu of 12-lead ECG
[4] Within 6 months prior to the ablation procedure (including prior to enrollment as part of routine clinical evaluations)
[5] TTE is required prior to discharge if the subject has symptoms suggestive of pericardial effusion and/or pericarditis
[6] Thrombus screening is required the day before or the day of the ablation procedure (see Section 9.7)
[7] Pregnancy test only required for women of childbearing potential
[8] Phrenic nerve evaluation is required if new phrenic nerve injury was observed/suspected in the ablation procedure and unresolved at previous assessment(s)
[9] Chest CT or MRI required if the subject experiences symptoms associated with PV stenosis

Table 7. Neurological Assessment Sub-Study additional/modified follow-up evaluations.

	Enrollment / Baseline	Ablation	Pre-Discharge	Day 10 Call	Day 30 Visit	Day 75 Call	Day 90 Visit	Day 180 Visit	Day 360 Visit	Repeat Ablation	Unsched.
**Cerebral MRI**			✓[10]				✓[11]				
**NIH Stroke Scale**	✓[12]		✓[13]		✓		✓[14]				
**MoCA**	✓[12]		✓[15]		✓		✓[14]				

[10] Post-procedure MRI performed within a recommended window 24 – 72 hours after the index ablation procedure, targeting 24 – 48 hours
[11] If SCE or SCL identified in post-procedure cerebral MRI
[12] Pre-procedure neurological assessments must be performed within 72 hours prior to the index ablation procedure
[13] Post-procedure NIH Stroke Scale must be performed within 72 hours after the index ablation procedure
[14] If new neurological symptoms/deficit identified in post-procedure evaluation were unresolved at Day 30
[15] Post-procedure Montreal Cognitive Assessment (MoCA) at least 24 hours and no more than 72 hours after the ablation procedure

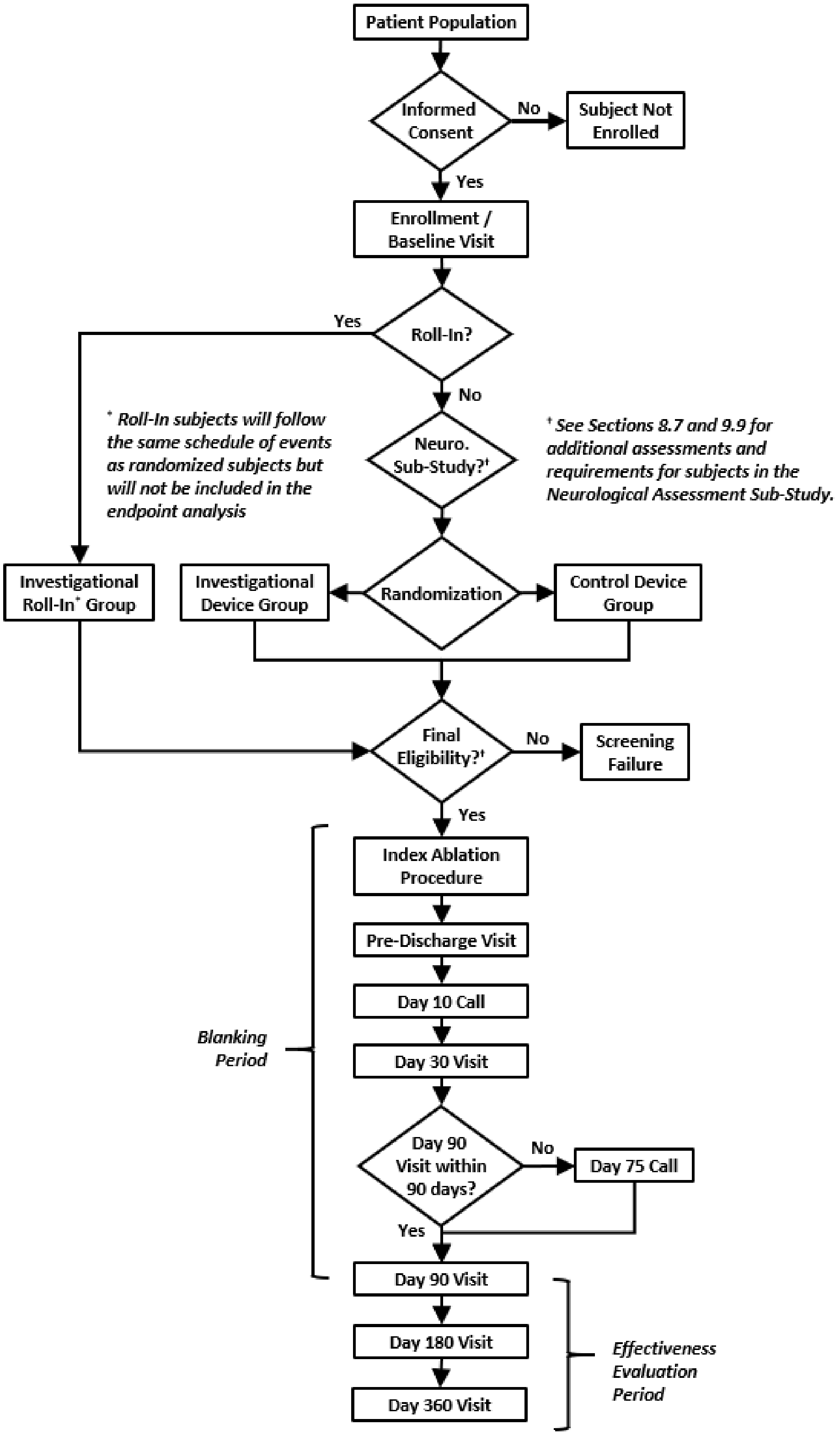

Figure 3. Study procedures flowchart.

10 ADVERSE EVENTS AND DEVICE DEFICIENCIES

10.1 DEFINITIONS

10.1.1 ADVERSE EVENTS

An adverse event (AE) is any untoward medical occurrence, unintended disease or injury, or untoward clinical signs (including abnormal laboratory findings) in subjects, users or other persons, whether or not related to the investigational medical device. This definition includes events related to the investigational device or the control device. This definition includes events related to the ablation procedure. For users or other persons, this definition is restricted to events related to the investigational medical device.

Conditions or diseases that are chronic but stable should not be recorded as an AE. For example, recurrence of AF or of a pre-existing atrial flutter (AFL) or atrial tachycardia (AT) and associated treatment (including repeat ablation or pharmacological or electrical cardioversion) are considered progression of pre-existing disease and do not meet the definition of an AE. Changes in a chronic condition or disease that are consistent with natural disease progression are not considered Aes, but these medical conditions should be adequately documented in the subject's medical history. An underlying disease or pre-existing condition will not be reported as an AE unless it significantly worsens in severity or frequency in a manner that is not attributed to natural progression.

10.1.2 SERIOUS ADVERSE EVENTS

A serious adverse event (SAE) is an adverse event that:

- Led to death
- Led to serious deterioration in the health of the subject, that either resulted in:
  - A life-threatening illness or injury, or
  - A permanent impairment of a body structure or body function, or
  - In-patient or prolonged hospitalization (> 24 hours), or
  - Medical or surgical intervention to prevent life-threatening illness or injury, or permanent impairment to a body structure or body function, or
  - Chronic disease
- Led to fetal distress, fetal death, or a congenital abnormality or birth defect

Hospitalization for a pre-existing condition or for a procedure that is required as part of this study, without a serious deterioration in health, is not an SAE.

10.1.3 ADVERSE DEVICE EFFECTS

An adverse device effect (ADE) is an AE related to the use of an investigational medical device. This definition includes AEs resulting from insufficient or inadequate instructions for use, deployment, installation, or operation, or any malfunction of the device. This definition includes any event resulting from use error or from intentional misuse of the investigational device.

A serious adverse device effect (SADE) is an ADE that that resulted in any of the consequences characteristic of a SAE.

An unanticipated serious adverse device effect (USADE) is an SADE which by its nature, incidence, severity, or outcome has not been identified in the current risk assessment. An anticipated serious adverse device effect (ASADE) is an effect which by its nature, incidence, severity, or outcome has been identified in the risk analysis report.

An unanticipated adverse device effect (UADE) is any serious adverse effect on health or safety or any life-threatening problem or death caused by, or associated with, a device, if that effect, problem, or death was not previously identified in nature, severity, or degree of incidence in the investigational plan or application (including a supplementary plan or application), or any other unanticipated serious problem associated with a device that relates to the rights, safety, or welfare of subjects.

10.1.4 Device Deficiency

A device deficiency is an inadequacy of a medical device with respect to its identity, quality, durability, reliability, safety, or performance. Device deficiencies include malfunctions, use errors, and inadequate labeling.

10.2 Assessment of Adverse Events

For the purposes of this study, the following AEs will be collected from the time informed consent is provided until the subject exits the study:

- All device or procedure related AEs, including AEs related to either the investigational or control device, and including possible relationship to the device or procedure
- All cardiovascular related AEs
- All SAEs, regardless of relationship to the device or procedure

Adverse events will be recorded, reported, and followed regardless of which device (investigational or control) was used to treat the subject.

AEs will be reported to the Sponsor using an electronic case report form (eCRF). The following information should be obtained for each AE:

- A description of the event including diagnosis based on signs, symptoms, and other supporting data
- Date of onset
- Date of awareness of the site
- Action taken including treatment
- Outcome including date of resolution
- Severity (see Table 8)
- Seriousness
- Expectedness (see Table 9)
- Relationship to the device or procedure (see Table 10)

- Relationship to the cardiovascular system, i.e., whether the AE is specifically related to the heart and the blood vessels or the circulation (cardiovascular related)
- Relationship to the respiratory system, i.e., whether the AE is specifically related to the lungs or respiration (pulmonary related)

Additional documentation may be requested by the Sponsor or designee including an event narrative detailing the clinical course of the event, correspondence with the IRB/EC, and hospital records.

The investigator is responsible for providing appropriate treatment for each adverse event and for following the event until resolution. If changes are identified in the assessment of a previously reported AE, an update to the original AE eCRF must be provided. Every effort should be made by the investigator to monitor all AEs until they are adequately resolved, stabilized (no further changes anticipated), or explained by another cause and the investigator believes that further evaluation is not warranted. At the time of study exit, any unresolved AEs should be reviewed to determine outcome.

In case of subject death, additional information should be collected if allowed by local law including date of death, cause of death, classification as cardiac or non-cardiac death, death certificate, and autopsy report. If a subject death occurs at a remote center, it is the site's responsibility to request information about the death from the remote center.

Table 8. Adverse event severity definitions.

**Mild**	Minimal transient impairment of body function or damage to body structure; and/or does not require intervention beyond monitoring.
**Moderate**	Moderate transient impairment of body function or damage to body structure; or requires intervention (e.g. medication) to prevent permanent impairment of body function or damage to body structure.
**Severe**	Life-threatening; results in permanent impairment of a body function or damage to a body structure; or requires significant intervention (e.g. surgery) to prevent permanent impairment of a body function or damage to a body structure.

Table 9. Adverse event expectedness definitions.

**Unanticipated**	Nature or severity of the event is not consistent with information about the condition under study or intervention in the protocol, consent form, instructions for use, or Investigator Brochure.
**Anticipated**	Event is known to be associated with the intervention or condition under study. Anticipated AEs include but are not limited to those listed in Table 12, as well as any anticipated AEs identified in the protocol for the PRECEPT study [22].

Table 10. Adverse event relationship to the device or procedure definitions.

**Not Related**	No temporal association between the device/procedure and the event; the cause of the event has been identified and is not related to the device/procedure; or the device/procedure cannot be implicated.
**Possibly Device-Related**	The study device (investigational or control) may have caused or contributed to the event.
**Device-Related**	The study device (investigational or control) directly caused or contributed to the event.
**Possibly Procedure-Related**	The event may be associated by timing and/or pathophysiology with the ablation procedure described in this protocol.
**Procedure-Related**	The event is directly associated by timing and/or pathophysiology with the ablation procedure described in this protocol.

10.3 Reporting Requirements

The investigator is responsible for reporting AEs according to the timelines in Table 11 and in accordance with local/regional laws and regulations. AEs are reported to the Sponsor using an Adverse Event eCRF.

Table 11. Adverse event reporting timelines.

Type of Event	Reporting Requirement
• **Serious Adverse Event (SAE)**<br>• **Serious Adverse Device Effect (SADE)**<br>• **Device Deficiency that could have led to an SADE**	Report to the Sponsor immediately upon awareness and no later than 72 hours after site awareness of the event. New or supporting information must be provided to the Sponsor in a timely manner as it becomes available. Submit to IRB/EC and regulatory authority according to local reporting requirements.
• **Unanticipated Adverse Device Effect (UADE)**<br>• **Unanticipated Serious Device Effect (USADE)**	Report to the Sponsor immediately upon awareness and no later than 72 hours after site awareness of the event. In the US, UADEs must be reported to the IRB as soon as possible but in no event later than 10 working days after the investigator first learns of the effect. Submit to IRB/EC and regulatory authority according to local reporting requirements
**Other Reportable AEs**	Report to the Sponsor in a timely manner, no later than 14 days after site awareness of the event.

An unavoidable adverse event is an AE inherent to the ablation procedure that, in the investigator's opinion, is expected to occur in all subjects for a limited period after the ablation procedure. These are not reportable AEs unless they occur after or last longer than the specified period. However, any adverse event classified as serious must be reported. Unavoidable adverse events include but are not limited to those listed below [43]:

- Anesthesia related nausea / vomiting for up to 24 hours
- Low-grade fever (less than 100°F or 37.8°C) for up to 48 hours
- Mild to moderate bruising / ecchymosis or pain in the groin area for up to 168 hours
- Sleep problems for up to 72 hours
- Back pain (related to laying on the procedure table) for up to 72 hours

Minor pericarditis limited to pleuritic chest discomfort with or without pericardial rub and ECG changes is generally not considered an AE in the context of AF ablation and is not reportable. A trace, trivial, or minor pericardial effusion that does not require intervention is generally not considered an AE as is not reportable unless it requires extended hospitalization [1, 21].

All device deficiencies related to the investigational device must be reported to the Sponsor through a Device Deficiency eCRF immediately upon awareness and no later than 72 hours after site awareness. Device deficiencies related to the control device will be reported according to the standard complaint handling procedure at the site.

Table 12. Anticipated adverse events.

- Acute respiratory distress syndrome
- Air embolism
- Allergic reaction, including anaphylaxis
- Anemia
- Anesthesia reaction, including nausea
- Aneurysm
- Angina / chest pain / discomfort
- Apnea – sedation induced
- Arrhythmia, including palpitations, bradycardia, tachycardia, proarrhythmia, and ECG changes
- Arteriovenous fistula
- Aspiration pneumonia
- Asthmatic attack
- Atelectasis
- Atrial fibrillation
- Atypical atrial flutter
- Bleeding complications
- Bronchial injury
- Bruise at puncture site
- Cardiac perforation
- Cardiac thromboembolism
- Cardiac or respiratory arrest
- Catheter entrapment
- Cerebrovascular accident (CVA) / stroke
- Complete heart block, temporary or permanent
- Conduction block
- Congestive heart failure (CHF), or exacerbation of existing CHF due to fluid overload
- Coronary artery dissection
- Coronary artery spasm
- Coronary artery thrombosis / occlusion
- Damage to ICD or implantable pacemaker
- Death
- Deep vein thrombosis
- Diaphragmatic paralysis
- Dislodgement of ICD or permanent pacing leads
- Disseminated intravascular coagulation
- Dyspnea / shortness of breath
- Endocarditis
- Esophageal ulceration / erythema
- Exacerbation of pre-existing arrhythmia
- Myocardial infarction
- Epistaxis, pharyngitis, or soft palate injury due to esophageal temperature probe
- Nausea
- Neck, back, or groin pain
- Neurological disorders including tremor, poor coordination, and headache
- Obstruction / perforation / damage to the vascular system
- Pericardial effusion, with or without tamponade
- Pericarditis
- Peripheral embolus or thromboembolism
- Peripheral nerve injury
- Persistent atrial septal defect
- Phlebitis
- Phrenic nerve damage
- Pleural effusion
- Pneumonia
- Pneumothorax
- Pseudoaneurysm
- Pulmonary edema
- Pulmonary embolism
- Pulmonary hemorrhage
- Pulmonary hypertension
- Pulmonary toxicity
- Pulmonary vein damage / dissection
- Pulmonary vein thrombus
- Pulmonary vein stenosis
- Radiation injury to skin, muscle, and/or organ
- Radiation-related cancers
- Renal failure
- Respiratory depression / failure
- Retroperitoneal hematoma
- Rhabdomyolysis
- Seizure
- Sepsis
- Skin burns
- Skin discoloration
- Skin irritation or rash
- Severe PV stenosis or complete occlusion
- Stiff left atrial syndrome
- Syncope / dizziness / fainting
- Tamponade
- Temperature elevation

• Expressive aphasia<br>• Fatigue<br>• Gastric reflux<br>• Gastroparesis / gastric hypomotility<br>• Headache<br>• Heart failure<br>• Hemoptysis<br>• Hemorrhage<br>• Hematoma (local) / ecchymosis<br>• Hemothorax<br>• Hypotension<br>• Hypoxia<br>• Increased creatinine phosphokinase (CPK) level<br>• Infection, localized or systemic<br>• Injury to skin, muscle, connective tissue due to body position or electrical cardioversion<br>• Laceration<br>• Leakage of air or blood into the lungs or other organs due to perforation<br>• Left atrial-esophageal fistula<br>• Liver toxicity<br>• Major bleeding, requiring surgery or transfusion	• Temporary AV, sinus node, or other heart block/damage<br>• Thrombocytopenia<br>• Thromboembolism<br>• Thrombosis<br>• Thyroid disorders<br>• Transient extremity numbness<br>• Transient ischemic attack (TIA)<br>• Unintended complete or incomplete AV, sinus node, or other heart block/damage<br>• Urinary retention<br>• Urinary tract injury or infection related to the urinary catheter<br>• Valvular damage / insufficiency<br>• Vascular bleeding / local hematomas / ecchymosis<br>• Vasovagal reactions<br>• Vessel wall damage<br>• Vision changes / abnormal vision<br>• Volume overload<br>• Vomiting<br>• Worsening obstructive, restrictive, or other pulmonary disease

Table 13. Primary adverse event definitions. Except as specified below, adverse events occurring more than 7 days after the index ablation procedure are excluded.

**Death**	Death during or after the ablation procedure that is directly related to the device or procedure.
**Myocardial infarction (MI)**	Myocardial infarction related to the ablation procedure in the presence of any one of the following criteria:<br>• ECG change indicative of new ischemia (ST segment elevation or depression) that persists for > 1 hour<br>• Development of new pathological Q waves on ECG<br>• Imaging evidence of new loss of viable myocardium or new regional wall motion abnormality
**Phrenic nerve paralysis**	Absence of phrenic nerve function after the ablation procedure as assessed by a sniff test or inhalation-exhalation fluoroscopy, not due to a pulmonary process such as atelectasis, that persists through the Day 30 visit. Phrenic nerve paralysis is considered permanent if it persists at least 12 months after the ablation procedure.
**Stroke / Cerebrovascular accident (CVA)**	Stroke diagnostic criteria<br>• Rapid onset of a focal or global neurological deficit with at least one of the following: change in level of consciousness, hemiplegia, hemiparesis, numbness or sensory loss affecting one side of the body, dysphasia or aphasia, hemianopia, amaurosis fugax, or other neurological signs or symptoms consistent with stroke<br>• Duration of a focal or global neurological deficit ≥24 hours; or <24 hours if therapeutic intervention(s) were performed (e.g., thrombolytic therapy or intracranial angioplasty); or available neuroimaging documents a new hemorrhage or infarct; or the neurological deficit results in death<br>• No other readily identifiable non-stroke cause for the clinical presentation (e.g., brain tumor, trauma, infection, hypoglycemia, peripheral lesion, pharmacological influences). Patients with nonfocal global encephalopathy will not be reported as a stroke without unequivocal evidence based on neuroimaging studies.<br>• Confirmation of the diagnosis by at least one of the following: neurology or neurosurgical specialist; neuroimaging procedure (MRI or CT scan or cerebral angiography); lumbar puncture (i.e., spinal fluid analysis diagnostic of intracranial hemorrhage)<br>Stroke will be determined by the consulting neurologist using the diagnostic criteria above and subsequent neuroimaging procedure (MRI, CT scan, or cerebral angiography).
**Transient ischemic attack (TIA)**	Acute episode of temporary (<24 hours) and focal loss of cerebral function of vascular (occlusive) origin as determined by the consulting neurologist and not meeting the definition of stroke/CVA.
**Thromboembolism**	Formation in a blood vessel of a clot (thrombus) that breaks loose and is carried by the blood from the site of origin to plug another vessel, resulting in deep vein thrombosis or pulmonary embolism.

**Cardiac tamponade / perforation**	Significant pericardial effusion of sufficient size to cause hemodynamic compromise and requiring pericardiocentesis based on hypotension, echocardiographic findings, or other clinical factors. Includes occurrence through 30 days after the ablation procedure.
**Major vascular access complications / bleeding**	Vascular access complication requiring blood transfusion greater than 2 units of packed red blood cells (PRBCs).
**Heart block**	New, persistent 2nd or 3rd degree AV block due to iatrogenic cause, not attributable to a vasovagal reaction, medication effect, or preexisting conduction disease, and requiring permanent pacing.
**Gastroparesis**	Gastroparesis that medically requires new hospitalization greater than 48 hours or prolongation of hospitalization by more than 48 hours.
**Severe pericarditis**	Pericarditis that results in pericardial effusion that is of sufficient size to cause hemodynamic compromise or requiring drainage based on hypotension, echocardiographic findings, or other clinical factors.
**Hospitalization (initial and prolonged) due to cardiovascular or pulmonary AE**	Cardiovascular or pulmonary adverse event that medically requires new hospitalization greater than 48 hours or prolongation of hospitalization by more than 48 hours. Excludes hospitalization due to AF/AFL/AT recurrence. Does not include hospitalization due to vascular access complications (see definition above for major vascular access complications/bleeding).
**Pulmonary vein (PV) stenosis**	Severe PV stenosis verified by imaging (chest CT or MRI), defined as ≥70% reduction in diameter of PV compared to the reference diameter (maximal diameter of the PV just distal to the stenosis). Includes occurrence through the Day 180 visit.
**Atrio-esophageal fistula**	Creation of a direct communication between the left atrium and esophagus necessitating surgical intervention or resulting in permanent impairment. Includes occurrence through the Day 90 visit.

11 Statistical Methods

Statistical analysis methods for the study are briefly outlined below. A comprehensive description of the statistical methods and rationale is provided in Statistical Analysis Plan CA-00034.

See Table 14 for subject cohort definitions.

Table 14. Subject cohorts for statistical analysis.

**Intention to Treat (ITT) Cohort**	Subjects who provide informed consent and are randomized.
**Primary Analysis Cohort (PAC)**	Subjects who provide informed consent, are randomized, and undergo insertion of the assigned study device (investigational or control), defined as the device emerging from the sheath into the bloodstream.
**Roll-In Cohort**	Subjects who provide informed consent, are assigned to Roll-In (not randomized), and undergo insertion of the investigational device, defined as the device emerging from the sheath into the bloodstream.
**Neurological Assessment Cohort**	Subjects who are assigned to and provide informed consent for the Neurological Assessment Sub-Study, are randomized, undergo ablation with the assigned study device (investigational or control), and undergo the required pre- and post-procedure assessments.

11.1 Primary Analyses

This study aims to demonstrate non-inferiority of both safety and effectiveness of the investigational device compared to the control device. The non-inferiority margin (NIM) for evaluating Primary Safety is 8%. The NIM for evaluating Primary Effectiveness is 15%. The Primary Analyses will be conducted using the Primary Analysis Cohort (PAC; see Table 14).

The study will be declared a success if the null hypothesis for both the primary safety and primary effectiveness endpoints are successfully rejected.

<u>Primary Safety Analysis</u>

The null hypothesis (H_0) for the Primary Safety Analysis is that the true rate of primary safety events for the investigational device (Q_I) is equal to or greater than the true rate for the control device (Q_C) plus a NIM of 0.08. The alternative hypothesis (H_A) is that the rate of primary safety events for the investigational arm (Q_I) is less than the rate of primary safety events for the control arm (Q_C) plus the NIM of 0.08.

H_0: $Q_I \geq Q_C + 0.08$
H_A: $Q_I < Q_C + 0.08$

The Primary Safety Analysis will be performed at a one-sided Type I error rate of α = 0.05 using the Farrington-Manning method. If the upper 95% confidence bound is less than 0.08, the study will be considered to have demonstrated safety of the investigational device.

Primary Effectiveness Analysis

The null hypothesis (H_0) is that the true rate of Primary Effectiveness Endpoint success (no failures through Day 360) for the investigational device (P_I) is less than or equal to the true rate for the control device (P_C) minus the NIM of 0.15. The alternative hypothesis (H_A) is that the success rate for the investigational arm (P_I) is greater than the success rate for the control device (P_C) minus the NIM of 0.15.

H_0: $P_I \leq P_C - 0.15$
H_A: $P_I > P_C - 0.15$

The Primary Effectiveness Analysis will be performed at a one-sided Type I error rate of α = 0.025 using the Farrington-Manning method. If the lower 97.5% confidence bound is greater than −0.15, the study will be considered to have demonstrated effectiveness of the investigational device.

11.1.1 Poolability across Sites

Data from different sites are expected to be poolable based on (i) consistent site selection criteria based on the Sponsor's standard operating procedures (SOPs), (ii) the use of a consistent clinical investigation plan with well-defined inclusion/exclusion criteria, and (iii) site monitoring to ensure compliance. To minimize the influence of any individual site, each site should not enroll more than 20% of the total enrollment.

A regression approach will be used to evaluate homogeneity of treatment effect (Primary Safety and Primary Effectiveness) across sites using the PAC. This will use a logistic regression model with fixed terms for randomized treatment group, site, and the interaction of treatment group and site. If necessary, Firth's adjustment will be used to handle sparse data. If heterogeneity across sites is found to be potentially significant ($p < 0.1$), additional analyses will be conducted to investigate sources of the apparent differences across sites. If data from different sites are not found to be poolable, a random-effects model will be fit to examine the impact of site heterogeneity on the primary endpoints.

11.1.2 Sensitivity Analysis

Multiple imputation of the primary effectiveness endpoint will be employed to address missing data. This will be based on a fully conditional specification logistic regression approach with 100 imputed data sets. Imputation will be performed separately by treatment group. The imputation model will include the following covariates: age, sex, and LA size. Details will be provided in the statistical analysis plan.

Tipping point analyses will also be conducted where the hazard of failure for those with missing outcomes are increased (and decreased) to explore the impact of potential missing not at random on the primary effectiveness analysis.

Reasons for missing data within the follow-up period will be reported. TTM compliance will be calculated for each subject in the PAC as the number of TTM recordings during a given period divided by the expected number of TTM recordings during that period. Expected transmissions will be estimated based on a 7-day week and a 31-day month.

11.1.3 Subgroup Analyses

Subgroup analyses will be performed to evaluate the primary safety and effectiveness endpoints within subgroups of subjects. Subgroup analysis will be based on at least the following demographic and baseline variables:

- Age
- Sex
- LA size

The primary endpoints will be assessed separately within each patient subgroup.

11.1.4 Sample Size

A sample size of 350 evaluable subjects is planned for the PAC. Power calculations were performed using the Farrington-Manning method [44]. A sample size of 350 subjects in the PAC (175 in each arm) provides 82.2% power for Primary Effectiveness and 84.0% power for Primary Safety in the Primary Analysis.

To ensure that investigators have adequate experience with the investigational device, each site will be permitted to use the investigational device in up to two enrolled non-randomized subjects (Roll-In Cohort). Subjects in the Roll-In Cohort will be analyzed separately from the PAC.

It is estimated that the attrition rate will be approximately 15%, leading to a planned randomized total of 410 subjects. The maximum number of Roll-In subjects will be 70. As a result, an overall maximum of 480 subjects will be enrolled in the study.

11.2 Secondary Analyses

If the Primary Analysis demonstrates safety and effectiveness of the investigational device, Secondary Analyses will be performed to test the following secondary hypotheses using the PAC:

1. Total Energy Application Time Superiority: The null hypothesis (H_0) is that the mean total energy application time during the ablation procedure for the investigational device (μ_{ETI}) is greater than or equal to the mean time for the control device (μ_{ETC}). The alternative hypothesis (H_A) is that the mean total energy application time for the investigational device is less.

H_0: $\mu_{ETI} \geq \mu_{ETC}$
H_A: $\mu_{ETI} < \mu_{ETC}$

2. Treatment Time Superiority: The null hypothesis (H_0) is that the mean treatment time for the investigational device (μ_{TTI}) is greater than or equal to the mean treatment time for the control device (μ_{TTC}). The alternative hypothesis (H_A) is that the mean treatment time for the investigational device is less.

   H_0: $\mu_{TTI} \geq \mu_{TTC}$
   H_A: $\mu_{TTI} < \mu_{TTC}$

3. Procedure Time Superiority: The null hypothesis (H_0) is that the mean procedure time for the investigational device (μ_{PTI}) is greater than or equal to the mean procedure time for the control device (μ_{PTC}). The alternative hypothesis (H_A) is that the mean procedure time for the investigational device is less.

   H_0: $\mu_{PTI} \geq \mu_{PTC}$
   H_A: $\mu_{PTI} < \mu_{PTC}$

4. Primary Effectiveness Superiority: The null hypothesis (H_0) is that the true rate of the Primary Effectiveness Endpoint for the investigational device (P_I) is less than or equal to the true rate for the control device (P_C). The alternative hypothesis (H_A) is that the rate for the investigational device is greater.

   H_0: $P_I \leq P_C$
   H_A: $P_I > P_C$

Sequential gate-keeping for the secondary endpoints will be used to maintain a one-sided Type I error rate $\alpha \leq 0.025$. The Secondary Analysis is described in greater detail in Statistical Analysis Plan CA-00034.

11.3 Additional Analyses

No formal statistical hypothesis will be formulated for any additional analyses. Inferential statistics for these analyses will not be included in the device label. Description of additional statistical analyses is provided in Statistical Analysis Plan CA-00034.

11.4 Neurological Assessment Sub-Study

No formal statistical hypotheses will be formulated for the Neurological Assessment Sub-Study endpoints. A sample size of 30 subjects from each arm is sufficient to allow qualitative comparison to published rates. Description of statistical analyses for the Neurological Assessment Sub-Study is provided in Statistical Analysis Plan CA-00034.

12 Risk Analysis

12.1 Potential Benefits

Available clinical data for the Sphere-9 Catheter and the Affera Mapping and Ablation System [32, 33, 34] suggest the following potential benefits of the investigational device:

- Improved durability of ablation lesions
- Reduced procedural and energy delivery time
- Safety benefits associated with PF ablation including reduced risk of thermal injury to the esophagus

There may be other benefits that are unforeseen at this time.

12.2 Potential Risks

Risks to subjects enrolled in this investigation include all risks currently associated with percutaneous electrophysiology procedures and RF catheter ablation procedures, which are well understood [1, 22, 36]. A brief summary of published complication rates can be found in Section 3.1.4. Anticipated adverse events are listed in Table 12, and some more common adverse events are listed in Section 10.3. Serious adverse events (SAEs) have also been reported for the PRECEPT pivotal IDE study of the THERMOCOOL SMARTTOUCH® SF catheter for treatment of persistent AF (NCT02817776) [21]. Specific risks are described in more detail below based on published literature and study results for catheter ablation for AF.

Some pain and/or ecchymosis at the groin access sites is common. The incidence of major vascular access complications has been reported between 0.2% and 1.5%. Access site complications can include hemorrhage or hematoma at the groin access sites after the ablation procedure [1]. The results of the PRECEPT study included hemorrhage reported as an SAE in 0.6% [21]. Access site complications can also include AV fistula or pseudoaneurysm. Ultrasound should be performed if AV fistula or pseudoaneurysm is suspected [1].

The ablation procedure may cause cardiac perforation leading to pericardial effusion and potentially tamponade. The incidence of tamponade has been reported between 0.2% to 5% [1]. Tamponade was reported as an SAE in 1.4% in the PRECEPT study [21]. Cardiac tamponade has been reported as the most frequent cause or periprocedural death [45]. Echocardiography should be performed if tamponade or significant pericardial effusion is suspected [1].

The reported incidence of cardiac valve damage is low (≤0.5%) [1, 20, 18]. Cardiac valve damage can be caused by catheter entrapment and should be confirmed with echocardiography when suspected [1].

The reported incidence of PV stenosis is less than 1%. PV stenosis can potentially lead to chest pain, shortness of breath, coughing, hemoptysis, or other pneumonia-like symptoms. There is also a risk of pulmonary hemorrhage, which has been reported in cases of pulmonary venous occlusion [46]. Chest CT or MR imaging should be performed if PV stenosis is suspected [1].

The reported incidence of coronary artery stenosis or occlusion is low (less than 0.1%) [1]; however, the incidence of myocardial infarction has been reported up to 0.4% in the literature [18], and myocardial infarction was reported as an SAE in 0.6% in the PRECEPT study [21].

There is a small risk of cardiac arrest (less than 0.1%) [1]. Heart block was reported as an SAE in approximately 0.6% in both the PRECEPT study and the SMART-AF study of the THERMOCOOL SMARTTOUCH® catheter for treatment of symptomatic paroxysmal AF (NCT01385202) [21, 26]. Arrhythmias are common after the ablation procedure [26] and may include atrial or ventricular fibrillation, tachycardia or bradycardia, possibly requiring cardioversion or pacemaker implantation.

Hypotension has been reported in previous studies of RF ablation for paroxysmal AF in up to 1.2% of subjects, and syncope has been reported at a similar rate [26, 23]. Unexplained hypotension should be evaluated promptly with echocardiography to rule out pericardial effusion or cardiac tamponade [1].

The incidence of death reported in the literature is 0.1% to 0.4% [1]. Two subject deaths (0.5%) were reported in the PRECEPT study [21].

Pleuritic chest pain with or without a “trace” pericardial effusion due to pericarditis is very common after catheter ablation for AF [1]. There is also a risk of pulmonary edema (1.4% reported as SAEs in the PRECEPT study) [21] or pneumothorax (reported between 0.1% to 0.4% in the literature) [20, 18]. There may be a risk of bronchial injury, particularly when ablating near the superior pulmonary veins [47, 48].

RF ablation near the posterior wall of the left atrium may cause esophagus injury, in rare cases leading to atrio-esophageal fistula, which is often fatal [1]. While esophageal injury is relatively common (15%) [49]; the incidence of atrio-esophageal fistula has been reported between 0.02% - 0.11% [1], with 0.2% reported in one single-center study [50]. When ablating near the esophagus with the control device in this study, reduced RF power is recommended to reduce the risk of thermal injury [1]. When ablating with the investigational device, only PF energy delivery is permitted on the posterior wall of the left atrium. PF ablation has not been shown to injure the esophagus [16].

RF ablation near the phrenic nerve can lead to temporary or permanent injury (diaphragmatic paralysis). The incidence of permanent phrenic nerve paralysis has been reported between 0% and 0.4 [1]. Diaphragmatic paralysis was reported as an SAE in one subject (0.3%) in the PRECEPT study [21]. Pacing maneuvers should be used to evaluate the location of the right phrenic nerve prior to any RF ablation near the right superior PV [1].

RF ablation on the posterior wall of the left atrium can injure the vagal nerves, which can lead to pyloric spasm and gastric hypomotility (gastroparesis). Gastric hypomotility has been reported with an incidence of up to 17%, and asymptomatic functional impairment has been reported in

up to 74% of patients. The risk can be reduced using the same techniques used to avoid esophageal injury [1], which are described above.

There is a risk of thromboembolism or air embolism leading to stroke or transient ischemic attack, asymptomatic cerebral emboli, or occlusion of other vessels (e.g., deep vein thrombosis or pulmonary embolism). The incidence of stroke or transient ischemic attack has been reported between 0% and 2%, and air embolism has been reported with an incidence less than 1% [1]. One stroke (0.3%) and two pulmonary embolisms (0.6%) were reported in the PRECEPT study [29].

Asymptomatic cerebral ischemia is often observed on cerebral MRI following catheter ablation for atrial fibrillation but has not been linked to decline in neurocognitive function [1]. A 2015 review defined a silent cerebral event (SCE) as an "acute new MRI-detected brain lesion typical to cerebral ischemia in a patient without clinically apparent neurological deficit", which is detected based on new DWI hyperintensity with corresponding ADC reduction. A silent cerebral lesion (SCL) is an SCE with corresponding FLAIR hyperintensity. This 2015 review found reported incidence of SCE following RF catheter ablation for AF (paroxysmal or persistent) between 6.8% and 24%, with reported incidence of SCL between 7.4% and 8.3% [51]. Publications since that time have reported widely varying incidence of post-ablation asymptomatic cerebral ischemia. A 2020 report of 285 AF patients undergoing catheter ablation with either RF or cryoballoon, targeting only PVI, found SCE in 23% [41]. Another 2020 report of 55 AF patients undergoing RF catheter ablation, including PVI and optional substrate ablation, found SCE in 42 – 67% depending on MRI parameters [52]. Meanwhile, a 2019 report of 97 paroxysmal AF patients undergoing ablation with a novel gold-tip RF ablation catheter found ischemia in 9.3%, which the authors called SCL but defined as being independent of FLAIR [40].

The risk of thromboembolism can be reduced by careful attention to anticoagulation before, during, and after the ablation procedure [1]. Continuous, uninterrupted anticoagulation is required in this study before, during, and after the ablation procedure, and the target activated clotting time (ACT) must be maintained while the ablation catheter is in the body.

Air embolism is most often caused by introduction of air through the transseptal sheath, including during catheter exchanges or associated with long apnea. Sheaths with access to the left atrium should be carefully managed according to the catheter instructions for use and training in order to minimize this risk. However, air embolism can also be caused by atrio-esophageal fistula, which should be excluded if air embolism occurs post-procedure [1].

Nausea or lightheadedness/fainting is common in procedures involving anesthesia. The subject may have an allergic reaction to anesthesia, sedatives, contrast dye, heparin, protamine, or other agents administered during the ablation procedure.

There is also a small risk of radiation injury, potentially increasing the lifetime risk of fatal malignancy (estimated at ≤0.1% for 60 minutes of fluoroscopy) [53].

While PF ablation may potentially reduce risks associated with RF catheter ablation such as damage to the phrenic nerves, vagal nerves, and esophagus, theoretical risks of PF ablation include arrhythmia induction, transient phrenic nerve injury, and asymptomatic cerebral emboli due to microbubble formation [15, 17].

The assessments included in this investigation are considered standard of care at the participating sites. Additional follow-up required as part of this investigation is not expected to increase risk to subjects.

12.3 Minimization of Risks

Risk analysis of the investigational device has been conducted in accordance with ISO 14971. Mitigating steps to address hazardous situations, use errors, and potential device malfunctions have been implemented to reduce the risks as low as possible. Risk mitigations have been confirmed in nonclinical bench, laboratory, and animal testing.

This investigation plan has been designed to minimize risk to participating subjects in several ways:

- Eligibility criteria have been designed to exclude subjects who may be at increased risk for complications from – or who are less likely to benefit from – catheter ablation for PerAF.
- Subjects will be screened to ensure compliance with inclusion and exclusion criteria.
- Investigators and sites will be selected for participation based on demonstrated experience with similar mapping and ablation procedures using the control device for the treatment of PerAF, and personnel will be trained on the use of the investigational devices prior to participation.
- Ablation settings available on the Affera Ablation System will be limited based on data obtained from animal and clinical studies.
- Affera personnel will be present for ablation procedures involving the investigational device and will monitor the use of the investigational device.
- Data monitoring will be conducted regularly to identify and address protocol deviations.
- A Data and Safety Monitoring Board will review data from the clinical investigation on an ongoing basis and advise the Sponsor regarding the safety and well-being of subjects any changes to the risk-benefit analysis.

The degree of risk to each subject relative to potential benefits of participation in the investigation should be evaluated by a qualified physician.

13 Study Conduct

13.1 Ethics

This investigation will be conducted in compliance with this clinical investigation plan, the signed clinical study agreement, the ethical principles stated in the latest version of the Declaration of Helsinki, 21 CFR Parts 11, 50, 54, 56, and 812, ISO 14155:2011 (Clinical Investigation of Medical Devices for Human Subjects – Good Clinical Practice), EU 2017/745 (Medical Device Regulations), and applicable local and international regulations in order to provide the greatest protection for the subject. Any deviations from this investigation plan that may affect the safety and welfare of study participants will be immediately reported to the Sponsor and to the Institutional Review Board (IRB) or Ethics Committee (EC) per each site's guidelines. Additional requirements imposed by the local IRB/EC or regulatory authority will be followed where appropriate.

13.2 Institutional Review Board / Ethics Committee

Prior to the initiation of the study, the clinical investigation plan and the patient informed consent form will be submitted to the Institutional Review Board (IRB) or Ethic Committee (EC) for approval. By signing the clinical study agreement, the investigator is assuring that an IRB/EC will be responsible for initial and continuing review of the proposed clinical study.

A copy of the IRB/EC approval letter for the protocol, the informed consent, and the protocol signature page must be submitted to the Sponsor or its designee prior to subject enrollment. The approval letter must refer to the specific protocol and the informed consent form. Any investigator who is also a member of the IRB/EC is not to participate in the protocol approval decision. This non-participation must be noted in the approval letter. The study site must maintain an accurate and complete record of all reports, documents, and other submissions made to the IRB/EC concerning this protocol.

The investigator must report any withdrawal of IRB/EC approval to the Sponsor or its designee within five (5) working days.

13.3 US FDA and Local Competent Authority

The study will be reviewed by the US FDA and other applicable regulatory authority as needed. The Sponsor or its designee is responsible for obtaining regulatory approval for the study. No subjects may be enrolled at a study site until written notification of the relevant local regulatory approval has been given by the Sponsor.

13.4 Informed Consent

It is the responsibility of the investigator to obtain informed consent from a subject prior to participation in this study. One original informed consent form is to be retained by the study site, and a signed copy is to be given to the subject. The informed consent process must be documented in the subject's source/medical record.

Any changes made to the informed consent must first be approved by the Sponsor or its designee. After approval by the Sponsor or its designee, the informed consent must be submitted to and approved by the reviewing IRB/EC. The informed consent form may be translated as appropriate.

If new information becomes available that can significantly affect a subject's future health and medical care, the information will be provided to the subjects in written form. The Sponsor is responsible for producing the written information and providing it to investigators who will provide it to the subjects. If applicable, all affected subjects will be asked to confirm their continued, informed consent in writing.

Any failure to obtain informed consent prior to use of the investigational device will be reported to the Sponsor and the reviewing IRB/EC within five (5) working days after the use.

13.5 Confidentiality and Data Protection

All information concerning the subjects in the study will be considered confidential by the Sponsor. Only authorized Sponsor representatives, the participating IRB/EC, local government authorities, or the FDA will have access to this information. All data used in analysis and reporting of this investigation will be performed using pseudonymized (key coded) data unless it is impossible to do so or involves disproportionate effort (e.g., if the subject's name is included as part of an image). Subjects will be identified on electronic case report forms (eCRFs) using a unique identifier (code). Any information that is obtained in connection with this study that can be identified with the subjects will remain confidential and will be made accessible only to authorized persons. Any data that may be published in scientific journals will not reveal the identity of the study participants.

All Sponsor representatives conducting this investigation in the US will have undergone training for HIPAA regulations and will comply with HIPAA including Protected Health Information (PHI), contact with subjects, medical records, copying of data. Patient informed consent will include authorization for access to PHI by Sponsor representatives and reflect applicable local regulations (e.g., GDPR in the European Union) as appropriate.

The investigator acknowledges that any and all information acquired from the Sponsor or its designee or developed or acquired in connection with the study are strictly confidential. The investigator will not disclose any confidential information to any third party nor use confidential information for any purpose without first obtaining the consent of Sponsor in writing. Such consent shall be deemed to have been given for disclosure to any person for whom the investigator is responsible at his/her center, but only so far as required for the purposes of the study, and, in the case of disclosures to staff, only if such staff are bound by obligations of confidentiality no less strict than those set out herein.

13.6 Insurance

The Sponsor will provide proof of appropriate insurance coverage for this clinical investigation as required by applicable local laws.

13.7 Data Quality Assurance

13.7.1 Site Selection

Potential investigational sites will be evaluated according to the Sponsor's SOPs to ensure that each site has appropriate facilities and personnel to conduct the study in compliance with the investigational plan. Investigator and site selection criteria will include demonstrated experience with similar mapping and ablation procedures using the control device for the treatment of PerAF.

13.7.2 Training

Prior to initiating enrollment at an investigational site, Sponsor representatives will provide appropriate device and protocol training in accordance with the documented study training plan. Investigators performing ablation procedures will be trained on the use of the Sphere-9 Catheter and the Affera Mapping and Ablation System. Site personnel training will include the clinical investigation plan, inclusion/exclusion criteria, case report forms (eCRFs), follow-up schedules, and regulatory requirements. In some cases, training of additional site staff may be performed by site staff who have been trained by Sponsor representatives.

13.7.3 Site Initiation

Prior to performing study related activities, requirements including but not limited to the following must be completed:

- IRB/EC approval
- Regulatory authority approval as required by local law
- Signed clinical study agreement and Investigator Agreement
- Financial disclosure forms
- Curriculum vitae (CV) for each investigator, lab director (if applicable), and any dedicated clinical research staff
- Documentation of delegated tasks
- Documented training, including investigational device training for all investigators performing ablation procedures and protocol training for all site staff performing study procedures

13.7.4 Case Report Forms

Electronic case report forms (eCRFs) will be used for this investigation, which are part of an Electronic Data Capture (EDC) system that meets 21 CFR Part 11 requirements.

Good Clinical Practice Guidelines require that Investigators maintain information in the study subject's medical records that corroborate data collected on the eCRFs. In order to comply with these regulatory requirements, the following information should be maintained:

- Medical history/physical condition of the study subject before involvement in the study sufficient to verify protocol entry criteria.

- Dated and signed notes on the day of entry into the study including the study investigator, study name, subject number assigned and a statement that consent was obtained.
- Dated and signed notes from each study subject visit with reference to the eCRFs for further information, if appropriate (for specific results of procedures and exams).
- Information related to adverse events.
- Study subject's condition upon completion of or withdrawal from the study.
- Discharge summaries/procedure reports.

The investigator is responsible for the accuracy and completeness of eCRFs. The investigator will electronically sign the eCRF to verify that he or she has reviewed the eCRF and attests to the validity of the entered data. eCRFs will be completed by the investigator within 14 days from the corresponding visit or bimonthly. Queries regarding eCRF entries must be answered within 14 days.

13.7.5 DOCUMENTATION

Data entered into eCRFs will be based on source documentation. Source documents are a subject's original documents, data, or records from before and during the study, related to the medical treatment and history of the patient, and from which study data are obtained. Source documents may include, for example, the subject's medical records, hospital charts, clinic charts, questionnaires, study files, and results of diagnostic tests such as laboratory tests, electrocardiograms, and imaging. If no standard source document exists to capture data required by the eCRF, a worksheet may be developed to record this data. Worksheets signed and dated by the investigator may serve as source documents. Data recorded by the mapping and ablation system during the ablation procedure may be transferred to the EDC system by site staff, and the EDC data will be considered the source. The eCRF may be considered the source document if no relevant source data exist (e.g., investigator classification of adverse events, date of awareness, reasons for study deviations, or patient ID).

13.7.6 DATA MONITORING

Sponsor representatives will periodically conduct monitoring, both in person and via telephone, written, and/or electronic communication, to ensure compliance with the investigation plan and applicable regulations and guidelines. A study specific monitoring plan will be developed to ensure compliance with the protocol and with applicable regulatory requirements. Monitoring visits may include, but will not be limited to, site qualification visits, site initiation visits, interim monitoring visits, and close-out visits.

Data monitoring activities may include, but will not be limited to:

- Ensuring compliance with the study protocol
- Verification of IRB/EC and regulatory approval
- Review of eCRFs for completeness and accuracy, including verification of eCRFs against source documentation
- Verification of study logs including training, screening, enrollment, and device accountability

- Verification of informed consent for enrolled subjects
- Queries and identification of corrective actions to resolve issues

The investigator agrees to provide access for these monitoring activities and to assist Sponsor representatives as needed during monitoring visits. The investigator also agrees to provide access to regulatory agencies if requested. Data monitoring will comply with local privacy regulations.

13.7.7 Device Accountability

The Sponsor will maintain documentation of all investigational devices shipped to the site. Investigational device labels will include the text "Investigational device" in a prominent location.

The investigator or a designee will maintain documentation of receipt, use, and return of all investigational devices (by lot or serial number) in a device accountability log until the conclusion of the study. Investigational devices are to be used only in accordance with this clinical investigation plan and under supervision of the investigator or a duly designated person. It is the investigator's responsibility to ensure that all investigational devices are kept in a secure location, with access limited to individuals authorized by the investigator.

All Sphere-9 Catheters (used and unused) and Affera Mapping and Ablation Systems will be returned to the Sponsor. The Catheter Extension Cable should also be returned to the Sponsor if possible. Returned single-use devices should be labeled with the subject identification number, date of use, and indication of any device deficiency or adverse event as appropriate. Used devices must be decontaminated per hospital policy prior to return. Tracking information will be retained by the site for shipped devices. Specific instructions for returning investigational devices will be provided to the sites by the Sponsor.

13.7.8 Data Management

Data will be collected using an Electronic Data Capture (EDC) system for clinical studies. eCRF data will be stored in a secure database with password-protected access and will be backed up on a regular schedule. Data validation will include programmed and manual data checks.

13.7.9 Core Labs

12-lead ECG, TTM, and Holter Monitor recordings during the effectiveness evaluation period will be sent to a core lab for analysis. All events will be analyzed for symptomatic or asymptomatic arrhythmia recurrence according to the definitions in this protocol. Evaluations of arrhythmia recurrence will be reviewed by a qualified physician. In case of disagreement between the core lab and the investigator regarding the rhythm analysis, a third-party cardiologist will review the recording to make a final rhythm determination.

Cerebral MR images will be transmitted to a core lab for analysis. Images will be analyzed for ischemia according to the definitions in this protocol. Evaluations will be reviewed by a qualified physician.

Any PV anatomy images (chest CT or MRI) collected in patients who experience symptoms suggestive of PV stenosis will be transmitted to a core lab for analysis. Images will be analyzed to assess PV stenosis according to the definitions in this protocol. Evaluations will be reviewed by a qualified physician.

13.8 Protocol Deviations

A protocol deviation is a failure to comply with the requirements specified within this clinical study protocol. An investigator is not allowed to deviate from the protocol without prior approval by the Sponsor. Protocol deviations that affect subjects' rights, safety, or well-being or the scientific integrity of the clinical investigation require prior review and documented approval from the IRB/EC. In the US, these protocol deviations require prior approval from the FDA.

Under emergency circumstances, deviations from the clinical investigation plan to protect the life, rights, safety or well-being of human subjects may proceed without prior approval of the Sponsor and the IRB/EC. These deviations will be reported to the Sponsor and the IRB/EC as soon as possible after site awareness. In the US, emergency protocol deviations to protect the life or physical well-being of a subject must be reported to the local IRB/EC within 5 working days after the emergency occurred. Emergency protocol deviations must be reported to regulatory bodies as required by local regulations.

Deviations must be documented on the appropriate Protocol Deviation eCRF. If a Clinical Monitor becomes aware that an Investigator is not complying with the signed Investigator's Agreement, the Investigational Plan, the requirements of ISO 14155:2011 or other applicable regulations, or any conditions of approval imposed by the reviewing EC, the Sponsor or designee will discuss with the site corrective and preventive actions for avoiding future deviations, including repeat training as deemed necessary.

Repeated serious protocol deviations will be closely monitored. If excessive deviations or a failure to reduce deviations is noted, the Sponsor reserves the right to suspend study enrollment or terminate the site from the study until a sufficient system is in place at the site to reduce further deviations. If disqualified, site is required to return all investigational devices unless this action would jeopardize the rights, safety, or welfare of a subject. The decision to resume study enrollment and treatment at a site will be made by the Sponsor in coordination with the investigator.

Protocol deviations will be analyzed by the Sponsor for impact to the overall integrity of the study.

13.9 Study Audits

Audits of the study may be conducted by the Sponsor, regulatory agencies, or a third party designated by the Sponsor to evaluate compliance with the protocol, Investigator Agreement, and written procedures. These audits may cover all involved parties, systems, and facilities supporting the conduct of the study and are independent of, and separate from, routine monitoring functions.

In the event that an Investigator is contacted by a regulatory agency regarding this study, the Investigator will notify the Sponsor or its designee immediately. The Investigator and research coordinator must be available to respond to reasonable requests and queries made during the inspection process. The Investigator must provide the Sponsor or designee with copies of all correspondence that may affect review of the current study. The Sponsor may provide needed assistance in responding to regulatory audits.

13.10 Record Keeping

Records to be maintained by the investigator include:

- Clinical investigation plan and amendments
- Signed clinical study agreement
- IRB/EC approval letters including informed consent form
- IRB/EC membership
- Curriculum vitae for each investigator, lab director (if applicable), and any dedicated clinical research staff
- Investigator's medical license
- Study logs including:
  - Study personnel/delegation log
  - Training log
  - Screening log
  - Enrollment log
  - Site visit/monitoring log
  - Device accountability log
- Subject records including:
  - Informed consent
  - eCRFs (stored electronically)
  - Source documentation to support eCRFs
  - Procedure reports, procedure nursing notes, and results of interventional procedures while enrolled in the study
  - Record of any complications, AES, and device problems/malfunctions, with supporting documentation
  - Records pertaining to subject deaths throughout the course of the study (including death records, death certificate, and autopsy report, if performed)
- Lab certification and lab test normal ranges
- Study correspondence
- Site training records
- Protocol deviations (if applicable)
- Financial disclosure forms
- Study reports including final report and periodic reports required by the IRB/EC
- Copy of all IRB/EC approved subject-related materials and/or study advertising materials
- Investigator Brochure, if required by local regulations
- Insurance certificate, if required by local regulations

The Sponsor reserves the right to secure additional medical documentation as needed for data clarification regarding subjects enrolled in the study.

13.10.1 Data Retention

Data generated for the study should be stored in a limited-access file area and be accessible only to representatives of the study site, the Sponsor and its representatives, and relevant health authorities/regulatory agencies. All reports and communications relating to study subjects will identify subjects only by initials and/or subject identification number. Complete subject identification will be kept at the study site.

Essential records that support the data collected in the study must be retained in compliance with GCP guidelines and local regulations. All site study records will be retained at the site for at least 2 years (or longer as required by local regulations) after the records are no longer required for purposes of supporting a pre-market approval application or until 2 years following the date of study closure as determined by the Sponsor. These documents may be retained for a longer period by agreement with the Sponsor or in compliance with local regulations. No records may be destroyed without the written consent of the Sponsor. It is the responsibility of the Sponsor to notify the investigator when records no longer need to be retained. The Investigator will take measures to ensure that these records are not accidentally damaged or destroyed.

If the investigator moves from the current investigational site, the Sponsor must be notified of the name of the person who will assume responsibility for maintenance of the records at the investigational site or the new address at which the records will be stored.

13.11 Reports

The Sponsor will prepare progress reports and a final study report in accordance with requirements established by local regulatory authorities. The sponsor will submit progress reports to all US IRBs/ECs and to the FDA at least yearly. The final study report will be submitted within six (6) months of closing the database at the end of the study.

Data collected as part of the Neurological Assessment Sub-Study will be reported separately from the main study.

13.11.1 Investigator Reports

The investigator will prepare a final report as well as periodic reports as required by the reviewing IRB/EC. In the US, investigators must submit progress reports to the Sponsor and the IRB/EC no less often than yearly.

The investigator shall provide the IRB/EC and the Sponsor with an accurate final report within 3 months after completion, termination, or discontinuation of the study or the investigator's part of the study. The final report may not precede final data submission which has not been monitored.

13.11.2 PUBLICATION

Affera is committed to the publication and dissemination of study results regardless of outcomes. This study will be registered at www.clinicaltrials.gov before enrollment begins.

Upon the prior written consent of Sponsor, investigators shall have the right to publish papers related to the Study as outlined in the clinical study agreement. Any publication or presentation of study results will acknowledge the financial support of the Sponsor.

14 Study Oversight

14.1 Study Advisory Committee

A Study Advisory Committee will be established that will be responsible for:

- Review of the clinical investigation plan
- Ensuring investigator compliance with the investigation plan
- Advising investigators regarding the investigational devices, ablation procedure, roll-in procedures, and medical concerns
- Advising the Sponsor regarding medical concerns identified during the study
- Review and interpretation of study results
- Providing strategy and consultation regarding publication of study results

14.2 Investigator Responsibilities

The clinical investigators will be responsible for:

- Obtaining approval (and renewals as appropriate) from the IRB/EC for the institution at which procedures will be performed
- Providing the Sponsor with the following prior to enrollment:
    - IRB/EC approval letters
    - Informed consent form approved by the IRB/EC
    - Signed clinical study agreement
    - Signed financial disclosure form
    - Curriculum vitae for each investigator
- Maintaining accurate and complete logs including study personnel/delegation and training, subject screening and enrollment, and site visit/monitoring
- Completing training, and ensuring training of research personnel, prior to enrollment
- Supervising research personnel
- Assuring that all subjects enrolled in the study conform to the subject selection criteria.
- Obtaining informed consent (including HIPAA language as appropriate) from all subjects before enrollment as described in 9.2.
- Maintaining accurate records of receipt, use, and return of investigational devices as described in 13.7.7
- Performing the study procedures according to this investigation plan
- Completing eCRFs accurately, and reviewing and signing eCRFs, as described in 13.7.4
- Maintaining sufficient source data to support eCRFs
- Making every effort to maintain contact with all enrolled subjects as described in 8.8
- Retaining study records as described in this protocol
- Preparing a final report and periodic reports required by the IRB/EC

14.3 Sponsor Responsibilities

The Sponsor will be responsible for:

- Ensuring adherence to Sponsor responsibilities under 21 CFR 812 and ISO 14155:2011

- Preparing study documents including the clinical investigation plan, eCRFs, study logs, and a draft informed consent form
- Obtaining IDE approval from FDA and communicating with FDA
- Preparing and obtaining approval for IDE supplements as necessary
- Informing investigators of their responsibilities
- Informing investigators of any new information about the study that may affect the health, safety, or welfare of subjects or may influence their decision to participate in the study
- Site initiation assessments and approvals
- Training of investigators and site research personnel
- Providing investigational devices to the site
- Data monitoring
- Data management
- Providing resources for statistical analysis and study report writing necessary to complete reporting of study results

14.4 Data and Safety Monitoring Board

The study will utilize a Data and Safety Monitoring Board (DSMB) including a statistician and three (3) individuals with relevant experience who are not participating investigators in this study. The DSMB will meet at regular intervals to review data from this clinical investigation and advise the Sponsor regarding the safety and well-being of subjects, any changes to the risk-benefit analysis, and the scientific validity of the study results.

If the DSMB determines the study should be suspended, discontinued, or otherwise modified because of safety concerns, the DSMB will prepare formal written recommendations to the Sponsor to consider final action. The Sponsor will notify the FDA and other regulatory authorities, as appropriate, of any such recommendation. Moreover, any safety concerns that the DSMB identifies will be verbally communicated to both the Sponsor and investigators as soon as possible, prior to written documentation.

A charter for the DSMB will be established that will include:

- Description of responsibilities
- Identification of members, including qualifications
- Declaration of possible conflicts of interest
- Frequency and format of meetings
- Description of communication procedures, including data flow

14.5 Clinical Events Committee

The study will utilize an independent Clinical Events Committee (CEC) including three (3) physicians who are not participating investigators in this study. The CEC will meet at frequent intervals to adjudicate endpoint-related adverse events, classify adverse events according to severity and association with the device or procedure, and adjudicate death classifications

(including primary cause and cardiac classification). The CEC may request additional information and assessments from the investigator to support adjudication.

A charter for the CEC will be established that will include:

- Description of responsibilities
- Identification of members, including qualifications
- Declaration of possible conflicts of interest
- Frequency and format of meetings
- Description of communication procedures, including data flow

14.6 PROTOCOL AMENDMENTS

The Sponsor will document any modifications to the protocol in the form of a written amendment and obtain IRB/EC approval. Substantial changes may also require approval from the FDA and/or Competent Authority prior to implementation.

14.7 SUSPENSION OR DISCONTINUATION OF THE STUDY

The Sponsor has the right to suspend or discontinue the study at any or all investigational sites for any safety, ethical, or administrative reason at any time. The Sponsor may stop inclusion of subjects at a specific site if no subjects have been enrolled for a period of three (3) months or if the site or investigators meet the criteria for disqualification described in Section 13.8.

An investigator or IRB/EC may discontinue participation or withdraw approval for the study, respectively, with suitable written notice to the Sponsor, the IRB/EC, and regulatory authorities as appropriate. If an investigator discontinues participation in the study, the investigator's responsibilities will be transferred to another investigator if possible. If an investigator's participation in the study ends for any reason, the investigator is required to return all documents and investigational devices to the Sponsor unless this action would jeopardize the rights, safety, or welfare of a subject.

In case of suspension or discontinuation, enrolled subjects who have already undergone treatment with the investigational devices will continue to be followed according to the investigation plan if possible. Plans for continued follow-up will be provided by the Sponsor.

15 References

[1] H. Calkins, G. Hindricks, R. Cappato, Y. Kim, E. Saad, L. Aguinaga, J. Akar, V. Badhwar, J. Brugada, J. Camm and P. Chen, "2017 HRS/EHRA/ECAS/APHRS/SOLAECE expert consensus statement on catheter and surgical ablation of atrial fibrillation," *Heart Rhythm,* vol. 14, no. 10, pp. e275-e444, 2017.

[2] G. Lippi, F. Sanchis-Gomar and G. Cervellin, "Global epidemiology of atrial fibrillation: An increasing epidemic and public health challenge," *International Journal of Stroke,* vol. 16, no. 2, pp. 217-21, 2021.

[3] D. M. Lloyd-Jones, T. J. Wang, E. P. Leip, M. G. Larson, D. Levy, R. S. Vasan, R. B. D'Agostino, J. M. Massaro, A. Beiser, P. A. Wolf and E. J. Benjamin, "Lifetime Risk for Development of Atrial Fibrillation: The Framingham Heart Study," *Circulation,* vol. 110, no. 9, pp. 1042-6, 2004.

[4] A. S. Go, E. M. Hylek, K. A. Phillips, Y. Chang, L. E. Henault, J. V. Selby and D. E. Singer, "Prevalence of Diagnosed Atrial Fibrillation in Adults," *Journal of the American Medical Association,* vol. 285, no. 18, pp. 2370-5, 2001.

[5] A. Alonso, S. K. Agarwal, E. Z. Soliman, M. Ambrose, A. M. Chamberlain, R. J. Prineas and A. R. Folsom, "Incidence of atrial fibrillation in whites and African-Americans: the Atherosclerosis Risk in Communities (ARIC) Study," *American Heart Journal,* vol. 158, no. 1, pp. 111-7, 2009.

[6] A. M. Wendelboe, G. E. Raskob, P. Angchaisuksiri, A. N. Blanco, H. Büller, H. Ddungu, J. D. Dvorak, B. J. Hunt, E. M. Hylek, A. Kakkar, S. V. Konstantinides, M. McCumber, C. McLintock, T. Urano and J. I. Weitz, "Global public awareness about atrial fibrillation," *Research and practice in thrombosis and haemostasis,* vol. 2, no. 1, pp. 49-57, 2018.

[7] J. F. Meschia, P. Merrill, E. Z. Soliman, V. J. Howard, K. M. Barrett, N. A. Zakai, D. Kleindorfer, M. Safford and G. Howard, "Racial Disparities in Awareness and Treatment of Atrial Fibrillation: The REasons for Geographic and Racial Differences in Stroke (REGARDS) Study," *Stroke,* vol. 41, no. 4, pp. 581-7, 2010.

[8] J. Frewen, C. Finucane, H. Cronin, C. Rice, P. M. Kearney, J. Harbison and R. A. Kenny, "Factors that influence awareness and treatment of atrial fibrillation in older adults," *QJM: An International Journal of Medicine,* vol. 106, no. 5, pp. 415-24, 2013.

[9] Biosense Webster, Inc, "P030031/S100 Summary of Safety and Effectiveness Data (SSED)," 30 September 2020. [Online]. Available: http://www.accessdata.fda.gov/cdrh_docs/pdf3/P030031S100B.pdf. [Accessed 11 October 2021].

[10] Medtronic Inc., "P100010/S098 Summary of Safety and Effectiveness Data (SSED)," 23 June 2020. [Online]. Available: http://www.accessdata.fda.gov/cdrh_docs/pdf10/P100010S098B.pdf. [Accessed 12 October 2021].

[11] R. L. Page, J. A. Joglar, M. A. Caldwell, H. Calkins, J. B. Conti, B. J. Deal, N. M. Estes III, M. E. Field, Z. D. Goldberger, S. C. Hamill and J. H. Indik, "2015 ACC/AHA/HRS guideline for the management of adult patients with supraventricular tachycardia: a report

of the American College of Cardiology/American Heart Association Task Force on Clinical Practice Guidelines and the Heart Rhythm Society," *Heart Rhythm,* vol. 13, no. 4, pp. e136-e221, 2016.

[12] D. L. Packer, D. B. Mark, R. A. Robb, K. H. Monahan, T. D. Bahnson, J. E. Poole, P. A. Noseworthy, Y. D. Rosenberg, N. Jeffries, B. Mitchell, G. C. Flaker, E. Pokushalov, A. Romanov, T. J. Bunch, G. Noelker, A. Ardashev, A. Revishvili, D. J. Wilber, R. Cappato, K. H. Kuck, G. Hindricks, D. W. Davies, P. R. Kowey, G. V. Naccarelli, J. A. Reiffel, J. P. Piccini, A. P. Silverstein, H. R. Al-Khalidi and K. L. Lee, "Effect of Catheter Ablation vs Antiarrhythmic Drug Therapy on Mortality, Stroke, Bleeding, and Cardiac Arrest Among Patients With Atrial Fibrillation: The CABANA Randomized Clinical Trial," *Journal of the American Medical Association,* vol. 321, no. 13, pp. 1261-74, 2019.

[13] D. Callans, E. Gerstenfeld, S. Dixit, E. Zado, M. Vanderhoff, J. REN and F. Marchlinski, "Efficacy of repeat pulmonary vein isolation procedures in patients with recurrent atrial fibrillation," *Journal of cardiovascular electrophysiology,* vol. 15, no. 9, pp. 1050-1055, 2004.

[14] V. Y. Reddy, P. Neuzil, J. S. Koruth, J. Petru, M. Funosako, H. Cochet, L. Sediva, M. Chovanec, S. R. Dukkipati and P. Jais, "Pulsed field ablation for pulmonary vein isolation in atrial fibrillation," *Journal of the American College of Cardiology,* vol. 74, no. 3, pp. 315-26, 2019.

[15] F. H. Wittkampf, R. van Es and K. Neven, "Electroporation and its Relevance for Cardiac Catheter Ablation," *Journal of the American College of Cardiology: Clinical Electrophysiology,* vol. 4, no. 11, pp. 1481-2, 2018.

[16] E. Maor, A. Sugrue, C. Witt, V. R. Vaidya, C. V. DeSimone, S. J. Asirvatham and S. Kapa, "Pulsed electric fields for cardiac ablation and beyond: A state-of-the-art review," *Heart Rhythm,* vol. 16, no. 7, pp. 1112-20, 2019.

[17] D. E. Haines and C. J. Bradley, "The Promise of Pulsed Field Ablation," *EP Lab Digest,* vol. 19, no. 12, 2019.

[18] A. Deshmukh, N. J. Patel, S. Pant, N. Shah, A. Chothani, K. Mehta, P. Grover, V. Singh, S. Vallurupalli, G. T. Savani, A. Badheka, T. Tuliani, K. Dabhadkar, G. Dibu, Y. M. Reddy, A. Sewani, M. Kowalski, R. Mitrani, H. Paydak and J. F. Viles-Gonzalez, "In-hospital complications associated with catheter ablation of atrial fibrillation in the United States between 2000 and 2010: analysis of 93,801 procedures," *Circulation,* vol. 128, no. 19, pp. 2104-12, 2013.

[19] R. Cappato, H. Calkins, S.-A. Chen, W. Davies, Y. Iesaka, J. Kalman, Y.-H. Kim, G. Klein, A. Natale, D. Packer, A. Skanes, F. Ambrogi and E. Biganzoli, "Updated Worldwide Survey on the Methods, Efficacy, and Safety of Catheter Ablation for Human Atrial Fibrillation," *Circulation: Arrhythmia and Electrophysiology,* vol. 3, no. 1, pp. 32-8, 2010.

[20] E. Arbelo, J. Brugada, C. Blomstrom-Lundqvist, C. Laroche, J. Kautzner, E. Pokushalov, P. Raatikainen, M. Efremidis, G. Hindricks, A. Barrera, A. Maggioni, L. Tavazzi and N. Dagres, "Contemporary management of patients undergoing atrial fibrillation ablation: in-hospital and 1-year follow-up findings from the ESC-EHRA atrial fibrillation ablation long-term registry," *European Heart Journal,* vol. 38, no. 17, pp. 1303-16, 2017.

[21] "Safety and Effectiveness of STSF Catheter Evaluated for Treating Symptomatic Persistent Atrial Fibrillation (PsAF) (PRECEPT)," U.S. National Library of Medicine, 12 January 2021. [Online]. Available: https://clinicaltrials.gov/ct2/show/NCT02817776. [Accessed 1 April 2021].

[22] Biosense Webster, Inc., "Prospective Review of the Safety and Effectiveness of the THERMOCOOL SMARTTOUCH® SF Catheter Evaluated for Treating Symptomatic PersistenT AF (PRECEPT)," 1 May 2018. [Online]. Available: https://clinicaltrials.gov/ProvidedDocs/76/NCT02817776/Prot_000.pdf. [Accessed 3 May 2021].

[23] "DiamondTemp™ Ablation System for the Treatment of Paroxysmal Atrial Fibrillation (DIAMOND-AF)," U.S. National Library of Medicine, 3 January 2021. [Online]. Available: https://clinicaltrials.gov/ct2/show/NCT03334630. [Accessed 1 April 2021].

[24] "SMART-SF Radiofrequency Ablation Safety Study," U.S. National Library of Medicine, 20 August 2018. [Online]. Available: https://clinicaltrials.gov/ct2/show/NCT02359890. [Accessed 1 April 2021].

[25] L. A. Chinitz, D. P. Melby, F. E. Marchlinski, C. Delaughter, R. S. Fishel, G. Monir, A. M. Patel, D. N. Gibson, C. A. Athill, L. M. Boo, R. Stagg and A. Natale, "Safety and efficiency of porous-tip contact-force catheter for drug-refractory symptomatic paroxysmal atrial fibrillation ablation: results from the SMART SF trial," *EP Europace,* vol. 20, pp. 392-400, 2018.

[26] "THERMOCOOL® SMARTTOUCH™ Catheter for the Treatment of Symptomatic Paroxysmal Atrial Fibrillation," U.S. National Library of Medicine, 26 January 2015. [Online]. Available: https://clinicaltrials.gov/ct2/show/NCT01385202. [Accessed 1 April 2021].

[27] A. Natale, V. Y. Reddy, G. Monir, D. J. Wilber, B. D. Lindsay, H. T. McElderry, C. Kantipudi, M. C. Mansour, D. P. Melby, D. L. Packer, H. Nakagawa, B. Zhang, R. B. Stagg, L. M. Boo and F. E. Marchlinski, "Paroxysmal AF Catheter Ablation With a Contact Force Sensing Catheter Results of the Prospective, Multicenter SMART-AF Trial," *Journal of the American College of Cardiology,* vol. 64, no. 7, pp. 647-56, 2014.

[28] J. A. Clarnette, A. G. Brooks, R. Mahajan, A. D. Elliott, D. J. Twomey, R. K. Pathak, S. Kumar, D. A. Munawar, G. D. Young, J. M. Kalman, D. H. Lau and P. Sanders, "Outcomes of persistent and long-standing persistent atrial fibrillation ablation: a systematic review and meta-analysis," *EP Europace,* vol. 20, no. FI_3, pp. f366-76, 2018.

[29] M. Mansour, H. Calkins, J. Osorio, S. J. Pollak, D. Melby, F. E. Marchlinski, C. A. Athill, C. Delaughter, A. M. Patel, P. J. Gentlesk, B. DeVille, L. Macle, K. A. Ellenbogen, S. R. Dukkipati, V. Y. Reddy and A. Natale, "Persistent Atrial Fibrillation Ablation With Contact Force–Sensing Catheter: The Prospective Multicenter PRECEPT Trial," *JACC: Clinical Electrophysiology,* vol. 6, no. 8, 2020.

[30] M. Rodgers, C. McKenna, S. Palmer, D. Chambers, S. Van Hout, S. Golder, C. Pepper, D. Todd and N. Woolacott, "Curative catheter ablation in atrial fibrillation and typical atrial flutter: systematic review and economic evaluation," NIHR Journals Library, 2008.

[31] J. Kautzner, P. Neuzil, H. Lambert, P. Peichl, J. Petru, R. Chihak, J. Skoda, D. Wichterle, E. Wissner, A. Yulzari and K. H. Kuck, "EFFICAS II: optimization of catheter contact force improves outcome of pulmonary vein isolation for paroxysmal atrial fibrillation," *EP Europace,* vol. 17, no. 8, pp. 1229-35, 2015.

[32] E. Anter, P. Neuzil, G. Rackauskas, P. Peichl, A. Aidietis, J. Kautzner, H. Nakagawa, W. M. Jackman, A. Natale and V. Y. Reddy, "A Lattice-Tip Temperature-Controlled Radiofrequency Ablation Catheter for Wide Thermal Lesions: First-in-Human Experience with Atrial Fibrillation," *JACC: Clinical Electrophysiology,* 2020.

[33] V. Y. Reddy, P. Neuzil, P. Peichl, G. Rackauska, E. Anter, J. Petru, M. Funasako, K. Minami, A. Aidietis, G. Marinskis, A. Natale, H. Nakagawa, W. M. Jackman and J. Kautzner, "A Lattice-Tip Temperature-Controlled Radiofrequency Ablation Catheter: Durability of Pulmonary Vein Isolation and Linear Lesion Block," *JACC: Clinical Electrophysiology,* 2020.

[34] V. Y. Reddy, E. Anter, G. Rackauskas, P. Peichl, J. S. Koruth, J. Petru, M. Funasako, K. Minami, A. Natale, P. Jais, H. Nakagawa, G. Marinskis, A. Aidietis, J. Kautzner and P. Neuzil, "A Lattice-Tip Focal Ablation Catheter that Toggles Between Radiofrequency and Pulsed Field Energy to Treat Atrial Fibrillation: A First-in-Human Trial," *Circulation: Arrythmia and Electrophysiology,* 2020.

[35] C. T. January, L. S. Wann, H. Calkins, M. E. Field, L. Y. Chen, K. L. Furie, J. E. Cigarroa, P. A. Heidenreich, J. C. Cleveland, K. T. Murray, P. T. Ellinor, J. B. Shea, M. D. Ezekowitz, C. M. Tracy and C. W. Yancy, "2019 AHA/ACC/HRS focused update of the 2014 AHA/ACC/HRS guideline for the management of patients with atrial fibrillation: a report of the ACC/AHA Task Force on Clinical Practice Guidelines and the HRS," *Journal of the American College of Cardiology,* vol. 71, no. 1, pp. 104-32, 2019.

[36] G. Hindricks, T. Potpara, N. Dagres, E. Arbelo, J. J. Bax, C. Blomström-Lundqvist, G. Boriani, M. Castella, G.-A. Dan, P. E. Dilaveris, L. Fauchier, G. Filippatos, J. M. Kalman and M. La Meir, "2020 ESC Guidelines for the diagnosis and management of atrial fibrillation developed in collaboration with the European Association for Cardio-Thoracic Surgery (EACTS)," *European Heart Journal,* vol. 42, no. 5, pp. 373-498, 2021.

[37] F. Gaita, D. Caponi, M. Pianelli, M. Scaglione, E. Toso, F. Cesarani, C. Boffano, G. Gandini, M. C. Valentini, R. De Ponti, F. Halimi and J. F. Leclercq, "Radiofrequency Catheter Ablation of Atrial Fibrillation: A Cause of Silent Thromboembolism?," *Circulation,* vol. 122, no. 17, pp. 1667-73, 2010.

[38] M. Grimaldi, V. Swarup, B. DeVille, J. Sussman, P. Jais, F. Gaita, M. Duytschaever, G. A. Ng, E. Daoud, D. Lakkireddy, R. Horton, A. Wickliffe, C. Ellis and L. Geller, "Importance of anticoagulation and postablation silent cerebral lesions: Subanalyses of REVOLUTION and reMARQable studies," *Pacing and Clinical Electrophysiology,* vol. 40, no. 12, pp. 1432-9, 2017.

[39] S. Miyazaki, T. Watanabe, T. Kajiyama, J. Iwasawa, S. Ichijo, H. Nakamura, H. Taniguchi, K. Hirao and Y. Iesaka, "Thromboembolic Risks of the Procedural Process in Second-Generation Cryoballoon Ablation Procedures," *Circulation: Arrhythmia and Electrophysiology,* vol. 10, no. 12, p. e005612, 2017.

[40] B. Schmidt, G. Szeplaki, B. Merkely, J. Kautzner, V. van Driel, F. Bourier, M. Kuniss, A. Bulava, G. Nolker, M. Khan, T. Lewalter, N. Klein, B. Wenzel, J. Chun and D. Shah, "Silent cerebral lesions and cognitive function after pulmonary vein isolation with an irrigated gold-tip catheter: REDUCE-TE Pilot study," *Journal of Cardiovascular Electrophysiology,* vol. 30, no. 6, pp. 877-85, 2019.

[41] M. Harada, Y. Motoike, Y. Nomura, A. Nishimura, M. Koshikawa, K. Murayama, Y. Ohno, E. Watanabe, Y. Ozaki and H. Izawa, "Factors Associated with Silent Cerebral Events During Atrial Fibrillation Ablation in Patients on Uninterrupted Oral Anticoagulation," *Journal of Cardiovascular Electrophysiology,* vol. 31, no. 11, pp. 2889-97, 2020.

[42] C. T. January, L. S. Wann, J. S. Alpert, H. Calkins, J. E. Cigarroa, J. B. Conti, P. T. Ellinor, M. D. Ezekowitz, M. E. Field, K. T. Murray and R. L. Sacco, "2014 AHA/ACC/HRS guideline for the management of patients with atrial fibrillation: a report of the American College of Cardiology/American Heart Association Task Force on Practice Guidelines and the Heart Rhythm Society," *Journal of the American College of Cardiology,* vol. 64, no. 21, pp. e1-76, 2014.

[43] Medtronic, Inc., "STOP Persistent AF Clinical Investigation Plan," 1 December 2017. [Online]. Available: https://clinicaltrials.gov/ProvidedDocs/41/NCT03012841/Prot_003.pdf. [Accessed 3 May 2021].

[44] C. P. Farrington and G. Manning, "Test statistics and sample size formulae for comparative binomial trials with null hypothesis of non-zero risk difference or non-unity relative risk," *Statistics in Medicine,* vol. 9, no. 12, pp. 1447-54, 1990.

[45] R. Cappato, H. Calkins, S.-A. Chen, W. Davies, Y. Iesaka, J. Kalman, Y.-H. Kim, G. Klein, A. Natale, D. Packer and A. Skanes, "Prevalence and Causes of Fatal Outcome in Catheter Ablation of Atrial Fibrillation," *Journal of the American College of Cardiology,* vol. 53, no. 19, pp. 1798-803, 2009.

[46] M. Hayashi, T. Minezawa, K. Imaizumi, Y. Sobue, E. Watanabe, Y. Ozaki and M. Okazawa, "Pulmonary Hemorrhage due to Pulmonary Venous Occlusion after Radiofrequency Catheter Ablation for Atrial Fibrillation," *Respiration,* vol. 86, no. 3, pp. 252-3, 2013.

[47] Y.-G. Li, M. Yang, Y. Li, Q. Wang, L. Yi and J. Sun, "Spatial relationship between left atrial roof or superior pulmonary veins and bronchi or pulmonary arteries by dual-source computed tomography: implication for preventing injury of bronchi and pulmonary arteries during atrial fibrillation ablation," *EP Europace,* vol. 13, no. 6, pp. 809-14, 2011.

[48] A. Sparks, J. Kozinn, A. Wimmer and J. R. Stewart, "Distal Left Main Bronchial Injury after Catheter Ablation for Atrial Fibrillation: Report of Two Cases," *Clinics in Surgery,* vol. 2, pp. 1-3, 2017.

[49] B. Yarlagadda, T. Deneke, M. Turagam, T. Dar, S. Paleti, V. Parikh, L. DiBiase, P. Halfbass, P. Santangeli, S. Mahapatra, J. Cheng, A. Russo, J. Edgerton, M. Mansour, J. Ruskin, S. Dukkipati, D. Wilber, V. Reddy, D. Packer, A. Natale and D. Lakkireddy, "Temporal relationships between esophageal injury type and progression in patients

undergoing atrial fibrillation catheter ablation," *Heart Rhythm,* vol. 16, no. 2, pp. 204-12, 2019.

[50] N. Dagres, G. Hindricks, H. Kottkamp, P. Sommer, T. Gaspar, K. Bode, A. Arya, D. Husser, L. S. Rallidis, D. T. Kremastinos and C. Piorkowski, "Complications of Atrial Fibrillation Ablation in a High-Volume Center in 1,000 Procedures: Still Cause for Concern?," *Journal of Cardiovascular Electrophysiology,* vol. 20, no. 9, pp. 1014-9, 2009.

[51] T. Deneke, P. Jais, M. Scaglione, R. Schmitt, L. Di Biase, G. Christopoulos, A. Schade, A. Mügge, M. Bansmann, K. Nentwich, P. Müller, J. Krug, M. Roos, P. Halbfass, A. Natale, F. Gaita and D. Haines, "Silent Cerebral Events/Lesions Related to Atrial Fibrillation Ablation: A Clinical Review," *Journal of Cardiovascular Electrophysiology,* vol. 26, no. 4, pp. 455-63, 2015.

[52] Y. Yu, X. Wang, X. Li, X. Zhou, S. Liao, W. Yang, J. Yu, F. Zhang, W. Ju, H. Chen, G. Yang, M. Li, K. Gu, L. Tang, Y. Xu, J. Y.-S. Chan, P. Kojodjojo, K. Cao, J. Fan, B. Yang and M. Chen, "Higher Incidence of Asymptomatic Cerebral Emboli After Atrial Fibrillation Ablation Found With High-Resolution Diffusion-Weighted Magnetic Resonance Imaging," *Circulation: Arryhthmia and Electrophysiology,* vol. 13, no. 1, p. e007548, 2020.

[53] L. Lickfett, M. Mahesh, C. Vasamreddy, D. Bradley, V. Jayam, Z. Eldadah, T. Dickfeld, D. Kearney, D. Dalal, B. Lüderitz, R. Berger and H. Calkins, "Radiation Exposure During Catheter Ablation of Atrial Fibrillation," *Circulation,* vol. 110, no. 19, pp. 3003-10, 2004.

TREATMENT OF PERSISTENT ATRIAL FIBRILLATION WITH THE SPHERE-9 MAPPING AND ABLATION CATHETER AND THE AFFERA MAPPING AND ABLATION SYSTEM

STATISTICAL ANALYSIS PLAN

Document Number	CA-00034
Version and Date	Revision C<br>03 June, 2022
Clinical Investigation Plan	CP-00009
Investigational Device	Sphere-9™ Mapping and Ablation Catheter with the Affera Mapping and Ablation System
Description	Prospective, multicenter, randomized interventional study to evaluate the safety and effectiveness of the Sphere-9 Mapping and Ablation Catheter with the Affera Mapping and Ablation System for the treatment of symptomatic persistent atrial fibrillation (PerAF) refractory or intolerant to drugs using radiofrequency (RF) and pulsed field (PF) ablation
Sponsor and Investigational Device Manufacturer	Affera, Inc.<br>320 Nevada St, Suite 401<br>Newton, MA 02460<br>USA

DISCLOSURE STATEMENT

This document contains information that is confidential and proprietary to Affera, Inc. This information is being provided to you solely for the purpose of evaluating or conducting a clinical study for Affera. You may disclose the contents of this document only to study personnel under your supervision who need to know the contents for this purpose. Otherwise, the contents of this document may not be disclosed without the prior authorization from Affera. The foregoing shall not apply to disclosure required by governmental regulations or laws. Any supplemental information that may be added to this document also is confidential and proprietary to Affera and must be kept in confidence in the same manner as the contents of this document.

TABLE OF CONTENTS

1 ABBREVIATIONS 4
2 INTRODUCTION 5
2.1 Background 5
2.1.1 Atrial Fibrillation 5
2.1.2 Demographic Profile of Atrial Fibrillation Patients 5
2.1.3 Ablation of Atrial Fibrillation 6
2.1.4 Safety of AF Ablation 7
2.1.5 Clinical Outcomes of AF Ablation 7
3 STUDY OBJECTIVES 8
3.1 Primary Study Objectives 8
3.2 Neurological Assessment Sub-Study Objective 8
4 STUDY ENDPOINTS 9
4.1 Primary Safety Endpoint 9
4.2 Primary Effectiveness Endpoint 9
4.3 Secondary Endpoints 10
4.4 Neurological Assessment Sub-Study 11
5 STATISTICAL METHODS 12
5.1 General Considerations 12
5.2 Primary Analyses 13
5.2.1 Poolability across Sites 14
5.2.2 Sensitivity Analysis 14
5.2.3 Subgroup Analyses 15
5.2.4 Sample Size 15
5.3 Secondary Analyses 16
5.4 Additional Analyses 18
5.5 Neurological Assessment Sub-Study 20
6 REFERENCES 21

Table of Figures

Figure 1. R code for Primary Effectiveness sample size calculation. 16
Figure 2. R code for Primary Safety sample size calculation. 16

Table of Tables

Table 1. Subject cohorts for statistical analysis. 13

Revision History

Revision	Change Order	Description
C	CO-002554	Record of approval and change history is in the EDCS

1 Abbreviations

**AAD**	Antiarrhythmic Drug
**AE**	Adverse Event
**AF**	Atrial Fibrillation
**CVA**	Cerebrovascular Accident
**MoCA**	Montreal Cognitive Assessment
**NIM**	Non-Inferiority Margin
**PerAF**	Persistent Atrial Fibrillation
**PF**	Pulsed Field
**PV**	Pulmonary Vein
**PVI**	Pulmonary Vein Isolation
**QOL**	Quality of Life
**RF**	Radiofrequency
**SAE**	Serious Adverse Event
**SAP**	Statistical Analysis Plan
**SCE**	Silent Cerebral Event
**SCL**	Silent Cerebral Lesion
**TIA**	Transient Ischemic Attack
**TTE**	Transthoracic Echocardiogram
**TTM**	Transtelephonic Monitoring

2 Introduction

The purpose of this Statistical Analysis Plan (SAP) is to document the methods and rationale for the statistical analysis to be performed as part of clinical investigation plan CP-00009 (Treatment of Persistent Atrial Fibrillation with the Sphere-9 Mapping and Ablation Catheter and the Affera Mapping and Ablation System). This is a prospective, multicenter, randomized clinical evaluation of the Sphere-9 Mapping and Ablation Catheter with the Affera Mapping and Ablation System. Subjects are randomly assigned 1:1 to receive treatment with either the Sphere-9 Mapping and Ablation Catheter and the Affera Mapping and Ablation System (investigational device) or the THERMOCOOL SMARTTOUCH® SF Catheter (control device). Subjects are blinded to treatment assignment.

This SAP includes analysis to be performed for the corresponding Pre-Market Approval (PMA) submission and the study final report. Additional statistical analysis not described in this SAP may also be performed.

2.1 Background

2.1.1 Atrial Fibrillation

Atrial fibrillation (AF) is a supraventricular tachycardia that manifests as a rapid, irregular atrial rhythm with no clearly defined P-wave on the electrocardiogram. AF that terminates within seven days is defined as paroxysmal, while AF lasting longer than seven days is defined as persistent. Long-standing persistent AF is defined as continuous AF persisting for longer than 12 months [1].

AF carries significant risks. AF leads to dramatically increased risk of stroke and mortality, particularly among patients with persistent forms of AF. Furthermore, AF is often symptomatic, leading to fatigue and reduced quality of life (QOL); however, it carries similar risks with or without symptoms [1].

2.1.2 Demographic Profile of Atrial Fibrillation Patients

AF affects an estimated 37 million people worldwide [2]. The lifetime risk of developing AF after age 40 was 26% and 23% for men and women, respectively, in the Framingham Heart Study [3]. AF incidence rises rapidly with advancing age, and approximately one-fourth of adults diagnosed with AF are at least 80 years old [3, 4]. AF incidence is higher among whites than among African Americans [5].

Awareness of atrial fibrillation is poor [6], and disparities in awareness and treatment of AF have been identified. Factors identified as being associated with lack of awareness of AF have included race, lower education, rural location, lower number of general practitioner visits, and lower cognition [7, 8].

In the recent PRECEPT and STOP Persistent AF studies for catheter ablation to treat persistent AF (PerAF), the mean age was 65 years, with a standard deviation of 9 years. Approximately

70% of treated subjects in each study were male. Less than 10% of subjects treated in each study were non-white, and less than 2% were Black or African American [9, 10].

2.1.3 Ablation of Atrial Fibrillation

Thermal ablation using radiofrequency (RF) energy has become a widely accepted treatment for many tachyarrhythmias and is considered first-line therapy in some cases [11, 1]. The success rate of RF catheter ablation for treating atrial fibrillation has been found to be superior to that of antiarrhythmic drugs. For example, the RAAFT-2 trial found that catheter ablation led to a 55% recurrence rate for atrial fibrillation or atrial tachycardia at 2 years follow-up compared to 72% recurrence using antiarrhythmic drugs [1]. Similarly, recurrence of AF, atrial flutter, or atrial tachycardia in the CABANA trial favored catheter ablation over drug therapy with a hazard ratio of 0.53, and analysis based on treatment received suggests a benefit from ablation in terms of death and hospitalization [12].

Catheter ablation is considered a reasonable treatment (Class IIa) for the treatment of symptomatic persistent AF refractory or intolerant to antiarrhythmic drugs. Two ablation catheters have been approved for treatment of drug-refractory persistent AF: the THERMOCOOL SMARTTOUCH® SF Catheter, which is a focal RF ablation catheter; and the Arctic Front Advance™ Cardiac Cryoablation Catheter. Electrical isolation of all pulmonary veins (PVs) is recommended (Class I) as part of all AF ablation procedures. However, PV reconnection is common, and repeat ablation is often necessary [1, 13].

Catheter ablation of AF is often accomplished by delivering focal RF energy in a point-by-point fashion to create circles (e.g. to isolate PVs) and/or lines (e.g. to block an arrhythmogenic channel). RF energy delivery through an electrode at the tip of an ablation catheter causes resistive heating in tissue, which, along with conductive heating, leads to thermal ablation of cardiac tissue. Features such as saline irrigation and temperature feedback can help to avoid the formation of thrombus and char due to blood heating during RF delivery [1].

Pulsed field (PF) ablation, also known as irreversible electroporation, relies on the application of non-thermal electrical pulses to form pores in the cell membranes of target tissue resulting in tissue apoptosis or necrosis. Recently published preclinical and clinical research suggests that PF ablation may be as effective as RF ablation while reducing risks associated with RF ablation such as damage to collateral structures [14, 15, 16, 17].

In the recent PRECEPT pivotal IDE study of the THERMOCOOL SMARTTOUCH® SF catheter for treatment of persistent AF (NCT02817776), only PV isolation was required; linear ablation lines including left atrial roof line, mitral isthmus line, left atrial floor line, and cavotricuspid isthmus line were only required to treat documented macro-reentrant atrial tachycardias. Results of the PRECEPT study showed that a left atrial roof line was delivered in just under half (48.6%) of the per-protocol group, and a cavotricuspid isthmus line was delivered in just over one-third (35.2%) of the per protocol group. A left inferior PV mitral line and other linear lesions were each delivered in less than 10% of patients in the per-protocol group (7.8% and 8.9%, respectively) [9].

2.1.4 Safety of AF Ablation

A review of data from the Nationwide Inpatient Sample between 2000 and 2010 found an overall incidence of complications of 6.3%, with a trend of increasing complications over that period [18]. A 2010 questionnaire-based survey of centers performing catheter ablation for atrial fibrillation reported a major complication rate of 4.5% [19]. A 2017 analysis of prospective data from the ESC-EHRA Atrial Fibrillation Ablation Long-Term Registry reported procedure-related complications in 7.8% [20]. In the recent PRECEPT pivotal IDE study of the THERMOCOOL SMARTTOUCH® SF catheter for treatment of persistent AF (NCT02817776), which used a similar primary safety endpoint to this protocol, the rate of the primary safety endpoint was 4.7% [21]. The PRECEPT study used an objective performance criterion (OPC) of 16%, based on an expected safety event rate of 8% and an 8% region of indifference, for analyzing the primary safety endpoint [22].

Pivotal IDE studies of focal catheter ablation for paroxysmal AF have also used similar primary safety endpoints. The DIAMOND-AF pivotal IDE study of the DiamondTemp ablation system for treatment of paroxysmal AF (NCT03334630) reported a primary safety event in 3.3% of subjects treated with the investigational device and in 6.6% of subjects treated with the control device (TactiCath™ Quartz) [23]. The SMART-SF study (NCT02359890), which evaluated the THERMOCOOL SMARTTOUCH® SF catheter for the treatment of paroxysmal AF, reported a primary adverse event in 2.6% of subjects (95% CI 0.7-6.5%). However, the SMART-SF study only evaluated early safety and acute effectiveness, so the chronic effectiveness of the treatments was not reported [24]. An OPC of 14% was used for analysis of the primary safety endpoint in SMART-SF [25]. The SMART-AF (NCT01385202) study was a pivotal IDE evaluating the THERMOCOOL SMARTTOUCH® catheter for treatment of paroxysmal AF, with primary safety and effectiveness endpoints similar to this protocol. The incidence of primary adverse events in SMART-AF was 9.9% (95% CI 5.8-15.6%) [26]. The OPC used for SMART-AF was not reported [27] but was presumably higher than the upper confidence bound of 15.6% based on the study's success.

2.1.5 Clinical Outcomes of AF Ablation

In a 2018 systematic literature review, single-procedure clinical success in treating persistent AF was estimated at 47% (95% CI 40-54%) across 18 cohorts between 2010 and 2015. Single-procedure clinical success in treating any AF with pulmonary vein isolation (PVI) plus linear ablation was estimated at 44% (95% CI 36-53%) across 10 cohorts over the same time period. Overall, this review found declining clinical success for AF ablation between 2001 and 2015 [28, 28]. The 2017 HRS consensus on AF ablation recommended a minimum acceptable success rate for treatment of persistent AF of 40% [1]. Subsequent single-arm pivotal IDE studies, including the PRECEPT study, used this minimum rate as an objective performance criterion for primary effectiveness. In the recent PRECEPT pivotal IDE study of the THERMOCOOL SMARTTOUCH® SF catheter for treatment of persistent AF (NCT02817776), primary effectiveness success was 59.3% through the end of the nine-month effectiveness evaluation period [21]. However, in contrast to this protocol, PRECEPT used a 6-month blanking and therapy consolidation period, and repeat ablation was allowed during that period (which occurred in 5.7%) [29].

3 Study Objectives

3.1 Primary Study Objectives

The objectives of this study are to evaluate the safety and effectiveness of the Sphere-9 Mapping and Ablation Catheter with the Affera Mapping and Ablation System for the treatment of symptomatic persistent atrial fibrillation (PerAF) refractory or intolerant to drugs using radiofrequency (RF) and pulsed field (PF) ablation. This study aims to demonstrate safety and effectiveness non-inferiority compared to the control device.

3.2 Neurological Assessment Sub-Study Objective

The objective of the Neurological Assessment Sub-Study is to evaluate the comparative incidence of acute cerebral ischemia (symptomatic and asymptomatic) as well as any associated neurological deficits.

4 Study Endpoints

4.1 Primary Safety Endpoint

The primary safety endpoint is the incidence of the following device- or procedure-related serious adverse events (SAEs) following the index ablation procedure:

Within 7 days:

- Death
- Myocardial infarction
- Phrenic nerve paralysis
- Transient ischemic attack (TIA)
- Stroke/cerebrovascular accident (CVA)
- Thromboembolism
- Major vascular access complications / bleeding
- Heart block
- Gastroparesis
- Severe pericarditis
- Hospitalization (initial and prolonged) due to cardiovascular or pulmonary AE‡

Within 30 days:

- Cardiac tamponade / perforation

Within 90 days:

- Atrio-esophageal fistula

Within 180 days:

- Pulmonary vein stenosis

‡ Excludes hospitalization due to AF/AFL/AT recurrence

An objective performance criterion (OPC) of 16% was used for the primary safety endpoint in the PRECEPT study of the THERMOCOOL SMARTTOUCH® SF catheter for treatment of persistent AF (NCT02817776) based on an expected rate of 8% with an 8% region of indifference [22]. The reported rate of the primary safety endpoint in that study was 4.7% [21]. Other recent studies of RF ablation for the treatment of AF (paroxysmal or persistent) have reported primary adverse event rates as high as 9.9% [26]. For this study, a non-inferiority margin (NIM) of 8% between the investigational group and the control group will be used for the primary analysis of the Primary Safety Endpoint.

4.2 Primary Effectiveness Endpoint

The primary effectiveness endpoint is freedom from documented recurrence of AF, atrial tachycardia (AT), or atrial flutter (AFL) based on electrocardiographic data through 12-month follow-up and excluding a 90-day blanking period. The following are considered primary effectiveness endpoint failures:

- Inability to isolate all targeted pulmonary veins during the index procedure.
- Ablation using devices other than the assigned study device for any left atrial ablation during the index procedure. (The assigned study device is the Sphere-9 Catheter for the investigational arm and the THERMOCOOL SMARTTOUCH SF for the control arm.)

- Any repeat ablation, surgical ablation, or arrhythmia surgery for treatment of recurrent AF/AT/AFL after the index procedure.
- Documented AF/AT/AFL recurrence after the 90-day blanking period.
- Class I or III AAD dose increase from the historic maximum ineffective dose (prior to the index procedure) or initiation of a new Class I or III AAD for treatment of AF/AT/AFL after the 90-day blanking period.
- DC cardioversion for AF/AT/AFL after the 90-day blanking period.

The blanking period is the first 90 days after the index ablation procedure. The effectiveness evaluation period will be from Day 91 through Day 360. AAD dose changes made at the Day 90 visit, within the follow-up window, will be considered to have occurred within the 90-day blanking period.

As discussed in Section 2.1.5, an objective performance criterion (OPC) of 40% was used for the primary effectiveness endpoint in the PRECEPT study of the THERMOCOOL SMARTTOUCH® SF catheter for treatment of persistent AF (NCT02817776) [22]. An OPC of 40% was proposed as the minimum chronic acceptable success rate in the 2017 HRS consensus but is somewhat conservative compared to the range of success rates reported in a more recent meta-analysis [1, 28]. The reported rate of the primary effectiveness endpoint in the PRECEPT study was 59.3%, nearly 20% greater than an OPC of 40% [21]. For this study, a non-inferiority margin (NIM) of 15% between the investigational group and the control group will be used for the primary analysis of the Primary Effectiveness Endpoint.

4.3 Secondary Endpoints

Secondary Safety Endpoints:

- Incidence of early onset (within 7 days of the index ablation procedure) serious adverse events (SAEs)
- Incidence of peri-procedural (>7 and ≤30 days) SAEs
- Incidence of late onset (>30 days) SAEs

Secondary Effectiveness Endpoints:

- Changes in Quality of Life (QOL) using the Atrial Fibrillation Effect on Quality of Life (AFEQT) Questionnaire and the SF-12v2 Health Survey
- Rate of acute procedural success, defined as confirmation of entrance block in all targeted pulmonary veins
- Rate of pulmonary vein isolation on a per-vein basis
- Rate of acute block across linear ablations
- Use of antiarrhythmic drugs (AADs) during the effectiveness evaluation period
- Freedom from documented recurrence of symptomatic AF/AFL/AT through 12-month follow-up and excluding the 90-day blanking period
- Freedom from documented recurrence of symptomatic AF/AFL/AT off antiarrhythmic drug therapy through 12-month follow-up and excluding the 90-day blanking period

Secondary Performance Endpoints:

- Investigator scoring of electroanatomical mapping
- Total fluoroscopy time during the ablation procedure
- Procedure time, defined as the time from elapsed from first venous access to last sheath removal
- Treatment time, defined as the time from the start of the first ablation delivery to the end of the last ablation delivery
- Total energy application time during the ablation procedure
- Ablation lesion sets delivered (ablation strategy)
- Ablation parameters per application
- Ablation energy type (RF or PF) used for each ablation lesion set

4.4 Neurological Assessment Sub-Study

The endpoints for the Neurological Assessment Sub-Study will be as follows:

- Incidence of silent cerebral events (SCE) and silent cerebral lesions (SCL) observed post-ablation
- Number, location, and size of observed SCE and SCL post-ablation, and during follow-up
- Change from baseline NIH Stroke Scale post-ablation and during follow-up
- Change from baseline Montreal Cognitive Assessment (MoCA) post-ablation and during follow-up

No acceptance criteria will be specified for the Neurological Assessment Sub-Study endpoints.

5 Statistical Methods

The objectives of this study are to evaluate the safety and effectiveness of the Sphere-9 Mapping and Ablation Catheter with the Affera Mapping and Ablation System for the treatment of symptomatic persistent atrial fibrillation (PerAF) refractory or intolerant to drugs using radiofrequency (RF) and pulsed field (PF) ablation. Subjects will be randomly assigned using a fixed 1:1 allocation to receive treatment with either the investigational device or the control device. Subjects will be blinded to treatment assignment. Randomization will be blocked and stratified by site and by enrollment in the Neurological Assessment Sub-Study.

5.1 General Considerations

Quantitative variables will be summarized using standard descriptive statistics: number of non-missing observations, mean, standard deviation, median, minimum, maximum, and 95% confidence interval (if appropriate). Number and percent of missing data, if any, will be summarized.

Categorical or semi-quantitative variables will be summarized using classical frequency statistics: number of non-missing observations, frequency, and percentage by category. Number and percent of missing data, if any, will be summarized. In analyzing variables involving a combination of events (e.g., Primary Effectiveness Failures, Primary Safety Events, specific types of adverse events), only one event per subject will be counted. Confidence intervals will be reported using the Wilson score interval (for individual variables) or the Farrington-Manning method (for difference between variables) as appropriate [30].

For survival function analysis, the Kaplan-Meier estimator will be used with the exponential Greenwood formula for confidence intervals. The log-rank test will be used for comparisons between treatment arms. Subjects without failure during the follow-up period will be censored at the date of last study contact recorded (e.g., last study visit, or study exit). In case of failure at the Day 360 visit, within the visit window but beyond study day 360, the date of failure will be set to study day 360 so that the failure is included in the survival analysis.

The study day is calculated as days since the index ablation procedure. The day of the index ablation procedure is study day 0. The blanking period extends from study day 0 through study day 90. The effectiveness evaluation period extends from study day 91 through study day 360.

Subject age will be reported as age at time of screening.

See Table 1 for subject cohort definitions.

Table 1. Subject cohorts for statistical analysis.

**Intention to Treat (ITT) Cohort**	Subjects who provide informed consent and are randomized.
**Primary Analysis Cohort (PAC)**	Subjects who provide informed consent, are randomized, and undergo insertion of the assigned study device (investigational or control), defined as the device emerging from the sheath into the bloodstream.
**Roll-In Cohort**	Subjects who provide informed consent, are assigned to Roll-In (not randomized), and undergo insertion of the investigational device, defined as the device emerging from the sheath into the bloodstream.
**Neurological Assessment Cohort**	Subjects who are assigned to and provide informed consent for the Neurological Assessment Sub-Study, are randomized, undergo ablation with the assigned study device (investigational or control), and undergo the required pre- and post-procedure assessments.

5.2 Primary Analyses

This study aims to demonstrate non-inferiority of both safety and effectiveness of the investigational device compared to the control device. The non-inferiority margin (NIM) for evaluating Primary Safety is 8%. The NIM for evaluating Primary Effectiveness is 15%. The Primary Analyses will be conducted using the PAC (see Table 1).

The study will be declared a success if the null hypothesis for both the primary safety and primary effectiveness endpoints are successfully rejected.

Primary Safety Analysis

The null hypothesis (H_0) for the Primary Safety Analysis is that the true rate of primary safety events for the investigational device (Q_I) is equal to or greater than the true rate for the control device (Q_C) plus a NIM of 0.08. The alternative hypothesis (H_A) is that the rate of primary safety events for the investigational arm (Q_I) is less than the rate of primary safety events for the control arm (Q_C) plus the NIM of 0.08.

$$H_0: Q_I \geq Q_C + 0.08$$
$$H_A: Q_I < Q_C + 0.08$$

The Primary Safety Analysis will be performed at a one-sided Type I error rate of $\alpha = 0.05$. The Farrington-Manning method will be used to calculate the upper 95% confidence bound for the difference ($Q_I - Q_C$) between the rate of the Primary Safety Endpoint in the investigational arm (Q_I) and the rate of the Primary Safety Endpoint in the control arm (Q_C). If the upper confidence

bound is less than 0.08, the study will be considered to have demonstrated safety of the investigational device. The *p*-value for the Farrington-Manning test will be reported.

<u>Primary Effectiveness Analysis</u>
The null hypothesis (H_0) is that the true rate of Primary Effectiveness Endpoint success (no failures through Day 360) for the investigational device (P_I) is less than or equal to the true rate for the control device (P_C) minus the NIM of 0.15. The alternative hypothesis (H_A) is that the success rate for the investigational arm (P_I) is greater than the success rate for the control device (P_C) minus the NIM of 0.15.

H_0: $P_I \leq P_C - 0.15$
H_A: $P_I > P_C - 0.15$

The Primary Effectiveness Analysis will be performed at a one-sided Type I error rate of $\alpha = 0.025$. The Farrington-Manning method will be used to calculate the lower 97.5% confidence bound for the difference ($P_I - P_C$) between the rate of the Primary Effectiveness Endpoint in the investigational arm (P_I) and the rate of the Primary Effectiveness Endpoint in the control arm (P_C). If the lower confidence bound is greater than −0.15, the study will be considered to have demonstrated effectiveness of the investigational device. The *p*-value for the Farrington-Manning test will be reported.

If the Primary Effectiveness Endpoint rate is determined to be less than 40% for either the investigational arm or the control arm, the Primary Effectiveness analysis will be difficult to interpret, and additional analyses will be necessary to explain the poor device performance.

5.2.1 Poolability across Sites

Data from different sites are expected to be poolable based on (i) consistent site selection criteria based on the Sponsor's standard operating procedures (SOPs), (ii) the use of a consistent clinical investigation plan with well-defined inclusion/exclusion criteria, and (iii) site monitoring to ensure compliance. To minimize the influence of any individual site, each site should not enroll more than 20% of the total enrollment.

A regression approach will be used to evaluate homogeneity of treatment effect (Primary Safety and Primary Effectiveness) across sites using the PAC. This will use a logistic regression model with fixed terms for randomized treatment group, site, and the interaction of treatment group and site. If necessary, Firth's adjustment will be used to handle sparse data. If heterogeneity across sites is found to be potentially significant ($p < 0.1$), additional analyses will be conducted to investigate sources of the apparent differences across sites. If data from different sites are not found to be poolable, a random-effects model will be fit to examine the impact of site heterogeneity on the primary endpoints.

5.2.2 Sensitivity Analysis

Multiple imputation of the primary effectiveness endpoint will be employed to address missing data. This will be based on a fully conditional specification logistic regression approach with 100 imputed data sets. Imputation will be performed separately by treatment group. The imputation

model will include the following covariates: age, sex, and LA size. Covariates will be included in the model in the order listed above. Results of the imputation will be summarized with the confidence interval for the difference between treatment groups for the primary effectiveness endpoint, as well as the corresponding non-inferiority *p*-value.

Tipping point analyses will also be conducted where the hazard of failure for those with missing outcomes are increased (and decreased) to explore the impact of potential missing not at random on the primary effectiveness analysis.

Reasons for missing data within the follow-up period will be reported. Transtelephonic monitoring (TTM) compliance will be calculated for each subject in the PAC as the number of TTM recordings during a given period divided by the expected number of TTM recordings during that period. Expected transmissions will be estimated based on a 7-day week and a 31-day month.

5.2.3 Subgroup Analyses

Subgroup analyses will be performed to evaluate the primary safety and effectiveness endpoints within subgroups of subjects. Subgroup analysis will be based on at least the following demographic and baseline variables:

- Age (<65 years and ≥65 years)
- Sex
- LA size (<45mm and ≥45mm)

The primary endpoints will be assessed separately within each patient subgroup. Results for each subgroup will be summarized by the endpoint rate for each treatment group, difference between treatment groups, and the corresponding nominal 95% confidence intervals. A regression approach will be used to assess heterogeneity of the treatment effect by subgroup, by including terms for treatment group, subgroup, and the interaction of treatment and subgroup. A *p*-value less than 0.1 for the interaction term will indicate evidence of heterogeneity and trigger additional analyses to explore and quantify the interaction.

5.2.4 Sample Size

A sample size of 350 evaluable subjects is planned for the PAC. Power calculations were performed using the Farrington-Manning method [30]. Results can be reproduced using `nBinomial` in the R package `gsDesign` as illustrated below [31].

The underlying rate of the Primary Effectiveness Endpoint is assumed to be 0.60 for both the investigational device and the control device for the purpose of sample size calculation. Assuming a one-sided Type I error rate of 0.025, a sample size of 175 subjects in each arm of the PAC provides 82.2% power to reject the null hypothesis and demonstrate that the Primary Effectiveness of the investigational device is non-inferior to that of the control device.

```
> nBinomial(p1 = 0.6, p2 = 0.6, alpha = 0.025, beta = 0.2,
+ delta0 = -0.15, sided = 1, outtype = 3, scale = "Difference", n = 350)
    n  n1  n2 alpha sided      beta     Power    sigma0    sigma1  p1  p2 delta0       p10       p20
1 350 175 175 0.025     1 0.1778555 0.8221445 0.9700864 0.9797959 0.6 0.6  -0.15 0.5204364 0.6704364
```

Figure 1. R code for Primary Effectiveness sample size calculation.

The underlying rate of the Primary Safety Endpoint is assumed to be 0.08 for both the investigational device and the control device for the purpose of sample size calculation. Assuming a one-sided Type I error rate of 0.05, a sample size of 175 subjects in each arm of the PAC provides 84.0% power to reject the null hypothesis and demonstrate that the true rate of the Primary Safety Endpoint for the investigational device is non-inferior to that of the control device.

```
> nBinomial(0.08, 0.08, alpha = 0.05, beta = 0.2,
+ delta0 = -0.08, sided = 1, outtype = 3, scale = "Difference", n = 350)
    n  n1  n2 alpha sided      beta     Power    sigma0    sigma1   p1   p2 delta0        p10       p20
1 350 175 175  0.05     1 0.1599471 0.8400529 0.5817939 0.5425864 0.08 0.08  -0.08 0.05530385 0.1353039
```

Figure 2. R code for Primary Safety sample size calculation.

To ensure that investigators have adequate experience with the investigational device, each site will be permitted to use the investigational device in up to two enrolled non-randomized subjects (Roll-In Cohort). Subjects in the Roll-In Cohort will be analyzed separately from the PAC.

It is estimated that the attrition rate will be approximately 15%, leading to a planned randomized total of 410 subjects. The maximum number of Roll-In subjects will be 70. As a result, an overall maximum of 480 subjects will be enrolled in the study.

5.3 SECONDARY ANALYSES

If the Primary Analysis demonstrates safety and effectiveness of the investigational device (study success), Secondary Analyses will be performed to test the following secondary hypotheses using the PAC:

1. Total Energy Application Time Superiority: The null hypothesis ($H_0^{(1)}$) is that the mean total energy application time during the ablation procedure for the investigational device (μ_{ETI}) is greater than or equal to the mean time for the control device (μ_{ETC}). The alternative hypothesis ($H_A^{(1)}$) is that the mean total energy application time for the investigational device is less.

$$H_0^{(1)}: \mu_{\mathrm{ETI}} \geq \mu_{\mathrm{ETC}}$$
$$H_A^{(1)}: \mu_{\mathrm{ETI}} < \mu_{\mathrm{ETC}}$$

2. Treatment Time Superiority: The null hypothesis ($H_0^{(2)}$) is that the mean treatment time for the investigational device (μ_{TTI}) is greater than or equal to the mean treatment time for

the control device (μ_{TTC}). The alternative hypothesis ($H_A^{(2)}$) is that the mean treatment time for the investigational device is less.

$$H_0^{(2)}: \mu_{TTI} \geq \mu_{TTC}$$
$$H_A^{(2)}: \mu_{TTI} < \mu_{TTC}$$

3. Procedure Time Superiority: The null hypothesis ($H_0^{(3)}$) is that the mean procedure time for the investigational device (μ_{PTI}) is greater than or equal to the mean procedure time for the control device (μ_{PTC}). The alternative hypothesis ($H_A^{(3)}$) is that the mean procedure time for the investigational device is less.

$$H_0^{(3)}: \mu_{PTI} \geq \mu_{PTC}$$
$$H_A^{(3)}: \mu_{PTI} < \mu_{PTC}$$

4. Primary Effectiveness Superiority: The null hypothesis ($H_0^{(4)}$) is that the true rate of the Primary Effectiveness Endpoint for the investigational device (P_I) is less than or equal to the true rate for the control device (P_C). The alternative hypothesis ($H_A^{(4)}$) is that the rate for the investigational device is greater.

$$H_0^{(4)}: P_I \leq P_C$$
$$H_A^{(4)}: P_I > P_C$$

Sequential gate-keeping will be used to maintain a one-sided Type I error rate $\alpha \leq 0.025$. The secondary hypotheses above will only be tested if the Primary Analysis is successful (i.e., both safety and effectiveness of the investigational device have been demonstrated). Testing of the secondary hypotheses will proceed sequentially in the order listed above. The α level for each hypothesis test will be determined based on the "fallback" method of Wiens [32, 33] as follows:

1. $H_0^{(1)}$ will be tested at $\alpha_1 = \alpha_1' = 0.005$.
2. $H_0^{(2)}$ will only be tested if $H_0^{(1)}$ was rejected. In that case, $H_0^{(2)}$ will be tested at the same α level used for testing the previous hypothesis ($\alpha_2 = \alpha_1 = 0.005$).
3. $H_0^{(3)}$ will only be tested if *both* $H_0^{(1)}$ and $H_0^{(2)}$ were tested and rejected. In that case, $H_0^{(3)}$ will be tested at the same α level used for testing both previous hypotheses ($\alpha_3 = \alpha_2 = 0.005$).
4. $H_0^{(4)}$ will be tested regardless of the outcome of the preceding hypothesis tests. The α level for testing $H_0^{(4)}$ will be chosen as follows:
   a. If *any* of $H_0^{(1)}$, $H_0^{(2)}$, or $H_0^{(3)}$ was not rejected (i.e., failed), $H_0^{(4)}$ will be tested at $\alpha_4 = \alpha_4' = 0.020$.
   b. However, if *all* of $H_0^{(1)}$, $H_0^{(2)}$, and $H_0^{(3)}$ were tested and rejected (i.e., succeeded), $H_0^{(4)}$ will be tested at $\alpha_4 = \alpha_4' + \alpha_3 = 0.020 + 0.005 = 0.025$.

Any other *p*-values presented will be nominal, without adjustment for multiple comparisons.

5.4 Additional Analyses

No formal statistical hypothesis will be formulated for any additional analyses. Inferential statistics for these analyses will not be included in the device label.

Demographic and baseline characteristics of subjects in each analysis cohort will be reported, including but not limited to age, gender, race, ethnicity, baseline medications (including AADs and anticoagulation), medical history (including CHA_2DS_2-VASc and NYHA), arrhythmia history, height, weight, body mass index (BMI), and TTE measurements (including left atrial diameter).

Additional analyses for the primary endpoints will be performed using the PAC. The 95% confidence interval will be reported for the rate of the Primary Effectiveness Endpoint for both the investigational arm and the control arm, as well as the 95% confidence interval for the difference in rates between the two arms. Kaplan-Meier analysis and log-rank test will be performed.

As an additional analysis, the Primary Effectiveness analyses will be repeated, and results reported, with the following additional Primary Effectiveness failure modes:

- Amiodarone dose increase from the historic maximum ineffective dose during the 90-day blanking period.
- Initiation of amiodarone during the blanking period if the subject has never taken amiodarone.

The 90% confidence interval will be reported for the rate of the Primary Safety Endpoint for the both the investigational arm and the control arm, as well as the 90% confidence interval for the difference in rates between the two arms.

Secondary Safety Endpoints will be evaluated across subjects randomized to each arm in the ITT Cohort and the PAC as well as subjects in the Roll-In Cohort. SAEs will summarized by causality and severity using descriptive statistics. Non-serious AEs will be evaluated across three time frames for subjects randomized to each arm (ITT Cohort and PAC) as well as subjects in the Roll-In Cohort:

- Non-serious AEs within 7 days of the ablation procedure
- Non-serious AEs > 7 days and ≤ 30 days from the ablation procedure
- Non-serious AEs > 30 days from the ablation procedure

Analysis of secondary effective endpoints will be performed using the PAC. For each of the following secondary effectiveness endpoints, descriptive statistics will be reported as well as the 95% confidence interval for the difference between the investigational and control arm:

- Changes in Quality of Life (QOL) using the Atrial Fibrillation Effect on Quality of Life (AFEQT) Questionnaire

- Changes in QOL using the SF-12v2 Health Survey (including the Physical Component Summary and the Mental Component Summary)
- Rate of acute procedural success, defined as confirmation of entrance block in all targeted PVs, evaluated across subjects in whom PVI was attempted
- Rate of PVI (per targeted PV)
- Rate of acute block across linear ablations, evaluated across the number of linear ablations attempted, both across all linear ablations and separately for each type of linear ablation including:
    - Cavotricuspid isthmus line
    - Left atrial roof, posterior, and/or inferior line
    - Mitral isthmus line
- Use of antiarrhythmic drugs (AADs)

For each of the following secondary effectiveness endpoints, the 95% confidence interval will be reported for both the investigational arm and the control arm, as well as the 95% confidence interval for the difference in rates between the two arms.

- Freedom from documented recurrence of symptomatic AF/AFL/AT through 12-month follow-up and excluding the 90-day blanking period
- Freedom from documented recurrence of symptomatic AF/AFL/AT off antiarrhythmic drug therapy through 12-month follow-up and excluding the 90-day blanking period

Kaplan-Meier analysis and log-rank tests will be performed for these endpoints.

Secondary Performance Endpoints will be evaluated for both the investigational and control arm using the PAC.

For investigator scoring of electroanatomical mapping, rate of scores of each value will be reported. The rate of scores of 3 or better and 4 or better will be reported. The 95% confidence interval for the difference in rates between the investigational and control arm will be reported separately for (i) the rate of scores of 3 or better and (ii) the rate of scores of 4 or better.

For each of the following secondary performance endpoints, descriptive statistics will be reported as well as the 95% confidence interval for the difference between the investigational and control arm:

- Total fluoroscopy time during the ablation procedure
- Procedure time
- Treatment time
- Total energy application time during the ablation procedure
- Ablation lesion sets delivered
- Ablation energy type (RF or PF) used for each ablation lesion set

Descriptive statistics will be reported for ablation parameters per application for the investigational device only.

For the investigational device, descriptive statistics related to energy delivery will be reported separately across (i) all energy deliveries, (ii) only RF deliveries, and (iii) only PF deliveries. The

proportion of lesion sets applied with RF, PF, and both RF and PF energy will be reported. Ablation lesion sets will include PVI, cavo-tricuspid isthmus line, LA roof/posterior/inferior ablation, and mitral isthmus line.

5.5 Neurological Assessment Sub-Study

An initial cohort of at least 60 randomized subjects (at least 30 from each arm) will be enrolled in the Neurological Assessment Sub-Study. No formal statistical hypotheses will be formulated for the Neurological Assessment Sub-Study endpoints. A sample size of 30 subjects from each arm is sufficient to allow qualitative comparison to published rates.

Statistical analysis for the Neurological Assessment Sub-Study will be performed with the Neurological Assessment Cohort. Summary statistics will be presented for incidence of silent cerebral events (SCE) and silent cerebral ischemia (SCL) [34] post-ablation by treatment group. Number and size of SCE and SCL will be summarized by treatment group using appropriate categories. Number of lesions will be assumed to be zero if imaging is not performed at a follow-up visit due to absence or resolution at a prior visit. Subgroup analysis may be performed to explore the associations with variables such as type of oral anticoagulation, ACT during the procedure, catheter exchanges, and transseptal crossings. Change from baseline NIH Stroke Scale and MoCA will be summarized by treatment group using appropriate categories.

6 REFERENCES

[1] H. Calkins, G. Hindricks, R. Cappato, Y. Kim, E. Saad, L. Aguinaga, J. Akar, V. Badhwar, J. Brugada, J. Camm and P. Chen, "2017 HRS/EHRA/ECAS/APHRS/SOLAECE expert consensus statement on catheter and surgical ablation of atrial fibrillation," *Heart Rhythm,* vol. 14, no. 10, pp. e275-e444, 2017.

[2] G. Lippi, F. Sanchis-Gomar and G. Cervellin, "Global epidemiology of atrial fibrillation: An increasing epidemic and public health challenge," *International Journal of Stroke,* vol. 16, no. 2, pp. 217-21, 2021.

[3] D. M. Lloyd-Jones, T. J. Wang, E. P. Leip, M. G. Larson, D. Levy, R. S. Vasan, R. B. D'Agostino, J. M. Massaro, A. Beiser, P. A. Wolf and E. J. Benjamin, "Lifetime Risk for Development of Atrial Fibrillation: The Framingham Heart Study," *Circulation,* vol. 110, no. 9, pp. 1042-6, 2004.

[4] A. S. Go, E. M. Hylek, K. A. Phillips, Y. Chang, L. E. Henault, J. V. Selby and D. E. Singer, "Prevalence of Diagnosed Atrial Fibrillation in Adults," *Journal of the American Medical Association,* vol. 285, no. 18, pp. 2370-5, 2001.

[5] A. Alonso, S. K. Agarwal, E. Z. Soliman, M. Ambrose, A. M. Chamberlain, R. J. Prineas and A. R. Folsom, "Incidence of atrial fibrillation in whites and African-Americans: the Atherosclerosis Risk in Communities (ARIC) Study," *American Heart Journal,* vol. 158, no. 1, pp. 111-7, 2009.

[6] A. M. Wendelboe, G. E. Raskob, P. Angchaisuksiri, A. N. Blanco, H. Büller, H. Ddungu, J. D. Dvorak, B. J. Hunt, E. M. Hylek, A. Kakkar, S. V. Konstantinides, M. McCumber, C. McLintock, T. Urano and J. I. Weitz, "Global public awareness about atrial fibrillation," *Research and practice in thrombosis and haemostasis,* vol. 2, no. 1, pp. 49-57, 2018.

[7] J. F. Meschia, P. Merrill, E. Z. Soliman, V. J. Howard, K. M. Barrett, N. A. Zakai, D. Kleindorfer, M. Safford and G. Howard, "Racial Disparities in Awareness and Treatment of Atrial Fibrillation: The REasons for Geographic and Racial Differences in Stroke (REGARDS) Study," *Stroke,* vol. 41, no. 4, pp. 581-7, 2010.

[8] J. Frewen, C. Finucane, H. Cronin, C. Rice, P. M. Kearney, J. Harbison and R. A. Kenny, "Factors that influence awareness and treatment of atrial fibrillation in older adults," *QJM: An International Journal of Medicine,* vol. 106, no. 5, pp. 415-24, 2013.

[9] Biosense Webster, Inc, "P030031/S100 Summary of Safety and Effectiveness Data (SSED)," 30 September 2020. [Online]. Available: http://www.accessdata.fda.gov/cdrh_docs/pdf3/P030031S100B.pdf. [Accessed 11 October 2021].

[10] Medtronic Inc., "P100010/S098 Summary of Safety and Effectiveness Data (SSED)," 23 June 2020. [Online]. Available: http://www.accessdata.fda.gov/cdrh_docs/pdf10/P100010S098B.pdf. [Accessed 12 October 2021].

[11] R. L. Page, J. A. Joglar, M. A. Caldwell, H. Calkins, J. B. Conti, B. J. Deal, N. M. Estes III, M. E. Field, Z. D. Goldberger, S. C. Hamill and J. H. Indik, "2015 ACC/AHA/HRS guideline for the management of adult patients with supraventricular tachycardia: a report

of the American College of Cardiology/American Heart Association Task Force on Clinical Practice Guidelines and the Heart Rhythm Society," *Heart Rhythm,* vol. 13, no. 4, pp. e136-e221, 2016.

[12] D. L. Packer, D. B. Mark, R. A. Robb, K. H. Monahan, T. D. Bahnson, J. E. Poole, P. A. Noseworthy, Y. D. Rosenberg, N. Jeffries, B. Mitchell, G. C. Flaker, E. Pokushalov, A. Romanov, T. J. Bunch, G. Noelker, A. Ardashev, A. Revishvili, D. J. Wilber, R. Cappato, K. H. Kuck, G. Hindricks, D. W. Davies, P. R. Kowey, G. V. Naccarelli, J. A. Reiffel, J. P. Piccini, A. P. Silverstein, H. R. Al-Khalidi and K. L. Lee, "Effect of Catheter Ablation vs Antiarrhythmic Drug Therapy on Mortality, Stroke, Bleeding, and Cardiac Arrest Among Patients With Atrial Fibrillation: The CABANA Randomized Clinical Trial," *Journal of the American Medical Association,* vol. 321, no. 13, pp. 1261-74, 2019.

[13] D. Callans, E. Gerstenfeld, S. Dixit, E. Zado, M. Vanderhoff, J. REN and F. Marchlinski, "Efficacy of repeat pulmonary vein isolation procedures in patients with recurrent atrial fibrillation," *Journal of cardiovascular electrophysiology,* vol. 15, no. 9, pp. 1050-1055, 2004.

[14] V. Y. Reddy, P. Neuzil, J. S. Koruth, J. Petru, M. Funosako, H. Cochet, L. Sediva, M. Chovanec, S. R. Dukkipati and P. Jais, "Pulsed field ablation for pulmonary vein isolation in atrial fibrillation," *Journal of the American College of Cardiology,* vol. 74, no. 3, pp. 315-26, 2019.

[15] F. H. Wittkampf, R. van Es and K. Neven, "Electroporation and its Relevance for Cardiac Catheter Ablation," *Journal of the American College of Cardiology: Clinical Electrophysiology,* vol. 4, no. 11, pp. 1481-2, 2018.

[16] E. Maor, A. Sugrue, C. Witt, V. R. Vaidya, C. V. DeSimone, S. J. Asirvatham and S. Kapa, "Pulsed electric fields for cardiac ablation and beyond: A state-of-the-art review," *Heart Rhythm,* vol. 16, no. 7, pp. 1112-20, 2019.

[17] D. E. Haines and C. J. Bradley, "The Promise of Pulsed Field Ablation," *EP Lab Digest,* vol. 19, no. 12, 2019.

[18] A. Deshmukh, N. J. Patel, S. Pant, N. Shah, A. Chothani, K. Mehta, P. Grover, V. Singh, S. Vallurupalli, G. T. Savani, A. Badheka, T. Tuliani, K. Dabhadkar, G. Dibu, Y. M. Reddy, A. Sewani, M. Kowalski, R. Mitrani, H. Paydak and J. F. Viles-Gonzalez, "In-hospital complications associated with catheter ablation of atrial fibrillation in the United States between 2000 and 2010: analysis of 93,801 procedures," *Circulation,* vol. 128, no. 19, pp. 2104-12, 2013.

[19] R. Cappato, H. Calkins, S.-A. Chen, W. Davies, Y. Iesaka, J. Kalman, Y.-H. Kim, G. Klein, A. Natale, D. Packer, A. Skanes, F. Ambrogi and E. Biganzoli, "Updated Worldwide Survey on the Methods, Efficacy, and Safety of Catheter Ablation for Human Atrial Fibrillation," *Circulation: Arrhythmia and Electrophysiology,* vol. 3, no. 1, pp. 32-8, 2010.

[20] E. Arbelo, J. Brugada, C. Blomstrom-Lundqvist, C. Laroche, J. Kautzner, E. Pokushalov, P. Raatikainen, M. Efremidis, G. Hindricks, A. Barrera, A. Maggioni, L. Tavazzi and N. Dagres, "Contemporary management of patients undergoing atrial fibrillation ablation: in-hospital and 1-year follow-up findings from the ESC-EHRA atrial fibrillation ablation long-term registry," *European Heart Journal,* vol. 38, no. 17, pp. 1303-16, 2017.

[21] "Safety and Effectiveness of STSF Catheter Evaluated for Treating Symptomatic Persistent Atrial Fibrillation (PsAF) (PRECEPT)," U.S. National Library of Medicine, 12 January 2021. [Online]. Available: https://clinicaltrials.gov/ct2/show/NCT02817776. [Accessed 1 April 2021].

[22] Biosense Webster, Inc., "Prospective Review of the Safety and Effectiveness of the THERMOCOOL SMARTTOUCH® SF Catheter Evaluated for Treating Symptomatic PersistenT AF (PRECEPT)," 1 May 2018. [Online]. Available: https://clinicaltrials.gov/ProvidedDocs/76/NCT02817776/Prot_000.pdf. [Accessed 3 May 2021].

[23] "DiamondTemp™ Ablation System for the Treatment of Paroxysmal Atrial Fibrillation (DIAMOND-AF)," U.S. National Library of Medicine, 3 January 2021. [Online]. Available: https://clinicaltrials.gov/ct2/show/NCT03334630. [Accessed 1 April 2021].

[24] "SMART-SF Radiofrequency Ablation Safety Study," U.S. National Library of Medicine, 20 August 2018. [Online]. Available: https://clinicaltrials.gov/ct2/show/NCT02359890. [Accessed 1 April 2021].

[25] L. A. Chinitz, D. P. Melby, F. E. Marchlinski, C. Delaughter, R. S. Fishel, G. Monir, A. M. Patel, D. N. Gibson, C. A. Athill, L. M. Boo, R. Stagg and A. Natale, "Safety and efficiency of porous-tip contact-force catheter for drug-refractory symptomatic paroxysmal atrial fibrillation ablation: results from the SMART SF trial," *EP Europace,* vol. 20, pp. 392-400, 2018.

[26] "THERMOCOOL® SMARTTOUCH™ Catheter for the Treatment of Symptomatic Paroxysmal Atrial Fibrillation," U.S. National Library of Medicine, 26 January 2015. [Online]. Available: https://clinicaltrials.gov/ct2/show/NCT01385202. [Accessed 1 April 2021].

[27] A. Natale, V. Y. Reddy, G. Monir, D. J. Wilber, B. D. Lindsay, H. T. McElderry, C. Kantipudi, M. C. Mansour, D. P. Melby, D. L. Packer, H. Nakagawa, B. Zhang, R. B. Stagg, L. M. Boo and F. E. Marchlinski, "Paroxysmal AF Catheter Ablation With a Contact Force Sensing Catheter Results of the Prospective, Multicenter SMART-AF Trial," *Journal of the American College of Cardiology,* vol. 64, no. 7, pp. 647-56, 2014.

[28] J. A. Clarnette, A. G. Brooks, R. Mahajan, A. D. Elliott, D. J. Twomey, R. K. Pathak, S. Kumar, D. A. Munawar, G. D. Young, J. M. Kalman, D. H. Lau and P. Sanders, "Outcomes of persistent and long-standing persistent atrial fibrillation ablation: a systematic review and meta-analysis," *EP Europace,* vol. 20, no. FI_3, pp. f366-76, 2018.

[29] M. Mansour, H. Calkins, J. Osorio, S. J. Pollak, D. Melby, F. E. Marchlinski, C. A. Athill, C. Delaughter, A. M. Patel, P. J. Gentlesk, B. DeVille, L. Macle, K. A. Ellenbogen, S. R. Dukkipati, V. Y. Reddy and A. Natale, "Persistent Atrial Fibrillation Ablation With Contact Force–Sensing Catheter: The Prospective Multicenter PRECEPT Trial," *JACC: Clinical Electrophysiology,* vol. 6, no. 8, 2020.

[30] C. P. Farrington and G. Manning, "Test statistics and sample size formulae for comparative binomial trials with null hypothesis of non-zero risk difference or non-unity relative risk," *Statistics in Medicine,* vol. 9, no. 12, pp. 1447-54, 1990.

[31] K. Anderson, "gsDesign package - RDocumentation," 13 March 2021. [Online]. Available: https://www.rdocumentation.org/packages/gsDesign/versions/3.2.0. [Accessed 28 April 2021].

[32] M. Huque and S. Mushti, "Alpha-recycling for the analyses of primary and secondary endpoints of clinical trials," 4 November 2015. [Online]. Available: https://www.bassconference.org/tutorials/BASS%202015%20Huque%20Mushti.pdf. [Accessed 10 November 2021].

[33] B. L. Wiens, "A fixed sequence Bonferroni procedure for testing multiple endpoints," *Pharmaceutical Statistics,* vol. 2, no. 3, pp. 211-5, 2003.

[34] T. Deneke, P. Jais, M. Scaglione, R. Schmitt, L. Di Biase, G. Christopoulos, A. Schade, A. Mügge, M. Bansmann, K. Nentwich, P. Müller, J. Krug, M. Roos, P. Halbfass, A. Natale, F. Gaita and D. Haines, "Silent Cerebral Events/Lesions Related to Atrial Fibrillation Ablation: A Clinical Review," *Journal of Cardiovascular Electrophysiology,* vol. 26, no. 4, pp. 455-63, 2015.
