# Supplementary material for: Dual-energy lattice-tip ablation system for persistent atrial fibrillation: a randomized trial
Source: Nat Med. 2024 May 17;30(8):2303–10. doi: 10.1038/s41591-024-03022-6 (PMC11333282; doi:10.1038/s41591-024-03022-6)
Supplement: Supplementary file 2 — Reporting Summary [file 41591_2024_3022_MOESM2_ESM.pdf]

nature portfolio

Corresponding author(s): Elad Anter

Last updated by author(s): Apr 25, 2024

Reporting Summary

Nature Portfolio wishes to improve the reproducibility of the work that we publish. This form provides structure for consistency and transparency in reporting. For further information on Nature Portfolio policies, see our Editorial Policies and the Editorial Policy Checklist.

Statistics

For all statistical analyses, confirm that the following items are present in the figure legend, table legend, main text, or Methods section.

n/a	Confirmed	
☐	☒	The exact sample size (*n*) for each experimental group/condition, given as a discrete number and unit of measurement
☐	☒	A statement on whether measurements were taken from distinct samples or whether the same sample was measured repeatedly
☐	☒	The statistical test(s) used AND whether they are one- or two-sided<br>*Only common tests should be described solely by name; describe more complex techniques in the Methods section.*
☒	☐	A description of all covariates tested
☐	☒	A description of any assumptions or corrections, such as tests of normality and adjustment for multiple comparisons
☐	☒	A full description of the statistical parameters including central tendency (e.g. means) or other basic estimates (e.g. regression coefficient) AND variation (e.g. standard deviation) or associated estimates of uncertainty (e.g. confidence intervals)
☐	☒	For null hypothesis testing, the test statistic (e.g. F, t, r) with confidence intervals, effect sizes, degrees of freedom and P value noted<br>*Give P values as exact values whenever suitable.*
☒	☐	For Bayesian analysis, information on the choice of priors and Markov chain Monte Carlo settings
☒	☐	For hierarchical and complex designs, identification of the appropriate level for tests and full reporting of outcomes
☒	☐	Estimates of effect sizes (e.g. Cohen's *d*, Pearson's *r*), indicating how they were calculated

Our web collection on statistics for biologists contains articles on many of the points above.

Software and code

Policy information about availability of computer code

Data collection	Medrio Electronic Data Capture (EDC) Software versions R41-R42 was used to collect study data.
Data analysis	Data analysis was performed using the SAS v9.4 statistical software.

For manuscripts utilizing custom algorithms or software that are central to the research but not yet described in published literature, software must be made available to editors and reviewers. We strongly encourage code deposition in a community repository (e.g. GitHub). See the Nature Portfolio guidelines for submitting code & software for further information.

Data

Policy information about availability of data

All manuscripts must include a data availability statement. This statement should provide the following information, where applicable:

- Accession codes, unique identifiers, or web links for publicly available datasets
- A description of any restrictions on data availability
- For clinical datasets or third party data, please ensure that the statement adheres to our policy

All supporting data are available within the article and supplement material(s). Source data will not be shared due to patient privacy and informed consents, including the potential for release of protected health information.

Research involving human participants, their data, or biological material

Policy information about studies with human participants or human data. See also policy information about sex, gender (identity/presentation), and sexual orientation and race, ethnicity and racism.

Reporting on sex and gender	Baseline patient characteristics, including sex, are provided in the manuscript. Findings do not apply to one sex or gender, and sex and gender were not prespecified in the study design. Self-reporting (e.g. discussion between patient and treating physician) was used to determine patient sex. Informed consent was obtained from all patients prior to enrollment in the study. Sex- or gender- based analyses are not provided, as it was not the main purpose of the clinical study.
Reporting on race, ethnicity, or other socially relevant groupings	All baseline patient characteristics (age, sex, comorbidities, years of diagnosis of persistent AF) are provided in Table 1 of the manuscript.
Population characteristics	All baseline patient characteristics (age, sex, comorbidities, years of diagnosis of persistent AF) are provided in Table 1 of the manuscript.
Recruitment	Participants were recruited by participating institutions in the clinical study based on direct conversations between healthcare providers and patients. All participants were required to meet inclusion/exclusion criteria prior to undergoing their index procedure. Randomization between the investigational and control arms serves as a method of experimental control for human clinical trials to reduce selection bias introduced by the sampling methods. All subjects were blinded to their treatment assignment.
Ethics oversight	FDA, and all IRB's/ ethics committees assigned to local hospitals in United States, Israel and Czech Republic

Note that full information on the approval of the study protocol must also be provided in the manuscript.

Field-specific reporting

Please select the one below that is the best fit for your research. If you are not sure, read the appropriate sections before making your selection.

☒ Life sciences ☐ Behavioural & social sciences ☐ Ecological, evolutionary & environmental sciences

For a reference copy of the document with all sections, see nature.com/documents/nr-reporting-summary-flat.pdf

Life sciences study design

All studies must disclose on these points even when the disclosure is negative.

Sample size	All study measurements were taken from distinct samples, with each trial participant as an independent sample. To achieve power >80% for testing each primary endpoint using the Farrington-Manning method, a sample size of 350 evaluable subjects (175 per arm) was required for the primary analysis cohort (i.e. randomized and treated subjects), with assumed underlying rate of 8%, noninferiority margin of 8% and one-sided alpha of 0.05 for the primary safety endpoint, and assumed underlying rate of 60%, noninferiority margin of 15% and one-sided alpha of 0.025 for the primary effectiveness endpoint. A total of 410 randomized subjects were planned based on a conservative 15% attrition estimate.
Data exclusions	No data was excluded from the analysis.
Replication	All study data entered into the clinical study database was 100% source data verified by clinical study monitors. Study data was monitored against source documentation, and queried for accuracy of data collection. An independent statistician reproduced analyses of the primary and several secondary endpoints reported in the manuscript, and all other data points were verified by a peer reviewer. An independent core lab adjudicated all arrhythmia monitoring transmissions, and an independent clinical events committee adjudicated all reported adverse events. All study measurements were taken from distinct samples, with each trial participant as an independent sample.
Randomization	Randomization was completed via an electronic data capture system, where randomization was blocked and stratified by site and by enrollment in a neurological sub-study. Randomized patients were blinded to their procedural assignment. Trial participants who met study eligibility criteria were randomized 1:1 to either the investigational arm or the control arm.
Blinding	Investigator blinding was not possible, as they were responsible for treatment of the patient with either the control or investigational device. An autonomous board responsible for data and safety monitoring supervised the participants' safety and the execution of the trial, while an independent clinical events committee that was blinded to the randomization, evaluated all outcomes of clinical significance. An independent core lab that was blinded to randomization adjudicated all arrhythmia transmissions. Randomized subjects were blinded to their treatment assignment.

Reporting for specific materials, systems and methods

We require information from authors about some types of materials, experimental systems and methods used in many studies. Here, indicate whether each material, system or method listed is relevant to your study. If you are not sure if a list item applies to your research, read the appropriate section before selecting a response.

Materials & experimental systems

n/a	Involved in the study
☒	☐ Antibodies
☒	☐ Eukaryotic cell lines
☒	☐ Palaeontology and archaeology
☒	☐ Animals and other organisms
☐	☒ Clinical data
☒	☐ Dual use research of concern
☒	☐ Plants

Methods

n/a	Involved in the study
☒	☐ ChIP-seq
☒	☐ Flow cytometry
☐	☒ MRI-based neuroimaging

Clinical data

Policy information about clinical studies

All manuscripts should comply with the ICMJE guidelines for publication of clinical research and a completed CONSORT checklist must be included with all submissions.

Clinical trial registration

NCT05120193

Study protocol

The full trial protocol is available in the Supplementary Material.

Data collection

Patients were treated between December 2021-December 2022, and followed for one year at their respective hospital institutions until December 2023.

Outcomes

The primary effectiveness endpoint was freedom from a composite of multiple failure modes, including: failure to acutely isolate all targeted PVs and complete all left atrial ablation with the assigned study device during the index procedure; repeat ablation at any time after the index procedure; and after a 3-month blanking period, documented occurrence of atrial tachyarrhythmia, escalation or initiation of Class I or III antiarrhythmic drugs, or cardioversion. Documented recurrence of atrial fibrillation, atrial tachycardia, or atrial flutter was based on either: (a) an episode ≥ thirty seconds in duration documented by ECG, TTM, or Holter monitor or (b) an episode covering an entire twelve-lead electrocardiogram recording lasting at least ten seconds. The study protocol included additional superiority testing contingent upon noninferiority of the primary endpoints. Energy application time, elapsed treatment time, total procedure time, and primary effectiveness were sequentially tested for superiority of the investigational device compared to the control device.

The primary safety endpoint was a composite of prespecified device- or procedure-related serious adverse events including death, atrio-esophageal fistula, stroke, myocardial infarction, cardiac tamponade/perforation, PV stenosis, phrenic nerve paralysis, transient ischemic attack, thromboembolism, major vascular access complications/bleeding, heart block, gastroparesis, severe pericarditis, or new or extended hospitalization for a cardiovascular or pulmonary adverse event. Adverse events were determined as serious if they (1) lead to death, (2) lead to serious deterioration in the health of the subject (including life-threatening illness or injury, permanent impairment of a body structure or function, >24-hour hospitalization, chronic disease, or medical or surgical intervention to prevent injury or permanent impairment of a body structure or function), (3) lead to fetal distress/ death, or a congenital abnormality or birth defect. Hospitalizations for pre-existing conditions or procedures without serious deterioration in health were not defined as serious. All primary adverse events were prespecified and their severity and association with the device or procedure were adjudicated by an independent clinical events committee.

Energy application time, elapsed treatment time, total procedure time, and primary effectiveness were sequentially tested for superiority of the investigational device compared to the control device as a prespecified secondary outcome. Pre-specified secondary effectiveness and performance endpoints included assessment of changes in quality of life, use of anti-arrhythmic drugs during the effectiveness evaluation period, procedure times, fluoroscopy time, and ablation lesion sets delivered. All other endpoints were based on post-hoc analyses.

Plants

Seed stocks

Report on the source of all seed stocks or other plant material used. If applicable, state the seed stock centre and catalogue number. If plant specimens were collected from the field, describe the collection location, date and sampling procedures.

Novel plant genotypes

Describe the methods by which all novel plant genotypes were produced. This includes those generated by transgenic approaches, gene editing, chemical/radiation-based mutagenesis and hybridization. For transgenic lines, describe the transformation method, the number of independent lines analyzed and the generation upon which experiments were performed. For gene-edited lines, describe the editor used, the endogenous sequence targeted for editing, the targeting guide RNA sequence (if applicable) and how the editor was applied.

Authentication

Describe any authentication procedures for each seed stock used or novel genotype generated. Describe any experiments used to assess the effect of a mutation and, where applicable, how potential secondary effects (e.g. second site T-DNA insertions, mosiacism, off-target gene editing) were examined.

Magnetic resonance imaging

Experimental design

Design type

MRI was obtained during a resting state. Subjects were randomized via a block design.

Design specifications

An initial cohort of at least 60 randomized subjects (at least 30 from the investigational or the control arm) underwent the cerebral MRI after the ablation procedure. MRI was performed within 72 hours after their index ablation procedure. Randomization was blocked and stratified by site and by enrollment in the neurological assessment sub-study.

Behavioral performance measures

Behavioral performance during the MRI was not monitored.

Acquisition

Imaging type(s)

Diffusion-weighted imaging (DWI) and fluid-attenuated inversion recovery (FLAIR) sequences

Field strength

1.5 T Scanner

Sequence & imaging parameters

T2-weighted axial FLAIR sequence: Slice thickness: 5 mm; Field of view: set to encompass the entire brain (typically 230mm); Matrix: 256; Repetition time (TR) may vary but is typically 8500 – 9000; Echo time (TE) may vary but is typically 100 – 120ms; Inversion Time (TI): 2500ms; Axial DWI sequence; Slice thickness: 5 mm; Field of view: set to encompass the entire brain (typically 230mm); Matrix: 128; Repetition time (TR) may vary but is typically 3200 – 3500ms; Echo time (TE) may vary but is typically between 80 and 100ms; Two distinct b values, 0 and 1000s/mm2, in three diffusion directions; For each DWI sequence, an apparent diffusion coefficient (ADC) map was obtained. The slices and orientation of the DWI and FLAIR sequences were matched.

Area of acquisition

A whole brain scan was used.

Diffusion MRI ☒ Used ☐ Not used

Parameters: Two distinct b values, 0 and 1000s/mm2, in three diffusion directions.

Preprocessing

Preprocessing software

Standard of care MRI pre-processing software was used in 12 different centers for 37 investigational patients, and 10 different centers for 35 control patients.

Normalization

Standard of care MRI pre-processing software was used in 12 different centers for 37 investigational patients, and 10 different centers for 35 control patients.

Normalization template

Standard of care MRI pre-processing software was used in 12 different centers for 37 investigational patients, and 10 different centers for 35 control patients.

Noise and artifact removal

Standard of care MRI pre-processing software was used in 12 different centers for 37 investigational patients, and 10 different centers for 35 control patients.

Volume censoring

Standard of care MRI pre-processing software was used in 12 different centers for 37 investigational patients, and 10 different centers for 35 control patients.

Statistical modeling & inference

Model type and settings

Statistical modeling was not performed for this analysis.

Effect(s) tested

Statistical modeling was not performed for this analysis.

Specify type of analysis: ☒ Whole brain ☐ ROI-based ☐ Both

Statistic type for inference

(See Eklund et al. 2016)

Statistical modeling was not performed for this analysis.

Correction

Statistical modeling was not performed for this analysis.

Models & analysis

n/a	Involved in the study
☒	☐ Functional and/or effective connectivity
☒	☐ Graph analysis
☒	☐ Multivariate modeling or predictive analysis
